# Are European diets healthy and sustainable? Evidence from nine countries using the planetary health diet framework

**DOI:** 10.1007/s00394-026-03929-5

**Published:** 2026-03-07

**Authors:** Agustin R. Miranda, Joseph M. M. Meunier, Sofia Romagosa Vilarnau, Anant Jani, Eric O. Verger

**Affiliations:** 1 MoISA, Univ Montpellier, CIHEAM-IAMM, CIRAD, INRAE, Institut Agro, IRD, Montpellier, France; 2 EuroHealthNet, Bruxelles, Belgium; 3 Heidelberg Institute of Global Health, University of Heidelberg, Heidelberg, Germany

**Keywords:** Nutrition surveys, Dietary patterns, EAT-Lancet diet, Sustainable nutrition

## Abstract

**Purpose:**

Contemporary food systems pose challenges for both human and planetary health. This study aimed to assess and compare adherence to the Planetary Health Diet (PHD) in nine European countries.

**Methods:**

Nationally representative dietary surveys (post-2013) from Estonia, Finland, France, Hungary, the Netherlands, Portugal, Spain, Switzerland, and the United Kingdom, with ≥ 2 non-consecutive 24-hour recalls, were used (*n* = 16,083 adults). Adherence to the PHD was assessed at two levels: (1) food group compliance, evaluated as the alignment with PHD target values and recommended ranges; (2) overall adherence, captured by three valid dietary indices. Multivariate regression analyses were conducted to identify associations with demographic factors.

**Results:**

Dietary patterns across Europe were characterised by insufficient intake of plant-based foods (whole grains, legumes, nuts, vegetables, and unsaturated oils) relative to PHD targets, alongside excessive consumption of foods to limit (red meat, saturated fats, and added sugars). Spain, Portugal, and the Netherlands showed comparatively better alignment with the PHD, whereas Hungary, the United Kingdom, and Estonia had the lowest scores. Red meat, particularly pork, and added sugars were the primary contributors to low scores across PHD indices. Being female, older, and having a higher level of education were positively associated with PHD adherence.

**Conclusion:**

European diets show systematic deviations from the PHD. Targeted and multilevel policies are needed to promote healthy and sustainable diets.

**Supplementary Information:**

The online version contains supplementary material available at 10.1007/s00394-026-03929-5.

## Introduction

The impact of current food systems on human and planetary health is a growing concern [1], prompting international and local calls for healthier and more sustainable food systems [2, 3]. Agriculture is a key driver of global change, responsible for up to 40% of land use, 85% of freshwater consumption, 90% of nitrogen and phosphorus use, and approximately 30% of greenhouse gas emissions (GHGE), largely from intensive animal production [4, 5]. Additionally, diets high in added sugars, meat, and saturated fats, and low in vegetables, fruits, and whole grains, increase the risk of non-communicable diseases (such as cardiovascular diseases, cancer, and mental health issues) [6–8]. Multiple mechanisms are involved, including poor nutritional quality, microbiota disruption, pro-inflammatory metabolites, impaired metabolism, and harmful preservatives [9, 10].

In 2019, the EAT-Lancet Commission introduced the Planetary Health Diet (PHD) as a global reference model for a healthy and sustainable diet [11]. Designed for an average daily intake of 2500 kcal, the diet emphasizes plant-based foods (including vegetables, fruits, legumes, whole grains, and nuts) as well as seafood, while recommending moderate consumption of eggs, poultry, and dairy, and limiting red meat, tubers, added sugars, and saturated fats [11]. Although the diet has faced critiques for its consumer-centred approach and limited applicability in low-resource contexts, it remains as a major contributor to global efforts toward food system transformation amid climate change [12, 13]. Evidence supports its benefits for environmental sustainability and human health. For example, high adherence to the PHD has been linked to up to 50% lower GHGE, 62% less land use, and potential prevention of 19–63% of deaths and 10–39% of cancers [14].

Translating global dietary guidelines such as the PHD into actionable policies requires understanding regional dietary patterns, as food systems are highly heterogeneous, reflecting diverse environmental, cultural, economic and health contexts across regions [15]. In this scenario, the European Union (EU) has committed to reducing GHGE by at least 55% by 2030 and 90% by 2050 compared to 1990 levels, identifying agriculture as a key emitting sector [16]. Non-EU countries, including Switzerland and the United Kingdom (UK), have adopted similar goals [17, 18]. At the same time, Europe faces rising rates of overnutrition and diet-related non-communicable diseases [19]. Achieving healthier and more sustainable diets therefore require not only individual behavioural change but also coordinated policy action across multiple governance levels [20]. Understanding the variability in dietary patterns across European populations is thus critical for informing targeted policies and interventions for sustainable diet promotion [21]. However, the current evidence base remains heterogeneous and often limited in scope, with many studies focusing on a narrow range of countries or overlooking the diversity of relevant food groups [21]. Some investigations examine only a small number of countries and rely on individual-level dietary records [22], whereas others encompass multiple countries but assess only selected dietary components, such as protein sources or the frequency of fruit and vegetable consumption [23, 24]. Even studies with broader geographic or dietary coverage frequently depend on aggregated mean intake data rather than individual-level consumption patterns [25]. To address this gap, this study utilised recent and harmonised population-based dietary survey data to assess and compare adherence to healthy and sustainable dietary patterns in line with the PHD across nine European countries.

## Methods

### Food consumption data

This study used the most recent nationally representative food consumption surveys from nine European countries: Estonia [26], Finland [27], France [28], Hungary [29], the Netherlands [30], Portugal [31], Spain [32], Switzerland [33], and the UK [34] (Table 1). Countries were selected based on the availability of national dietary survey data collected after 2013 using at least two non-consecutive 24-hour dietary recalls. For each country, data on food consumption (grams per day, g/d), were obtained for the adult population (≥ 18 years old). Data access was obtained from the data owners, European Food Safety Authority, and public repositories. All surveys complied with the Declaration of Helsinki and received ethics committee approval. Sampling flow chart is available in Supplementary Material (Fig. S1).

**Table 1:** Characteristics of the National dietary surveys

Country	Survey name	Year	*N* ^a^	Women	Men	Days^b^
Estonia	RTU	2013–2015	2649	1764	885	2
Finland	FINDIET	2017	1488	780	708	2
France	INCA3	2014–2015	2121	1234	887	3
Hungary	HU-EU-Menu	2018–2020	1056	528	528	2
The Netherlands	DNFCS	2019–2021	1747	867	880	2
Portugal	IAN-AF	2015–2016	3764	1994	1770	2
Spain	ENALIA 2	2014–2015	933	532	401	2
Switzerland	menuCH	2014–2015	2057	1128	929	2
United Kingdom	NDNS	2020	524	303	221	4

^a^Only adults

^b^Maximum number of 24-hour dietary recall days

Food consumption was categorised into key groups based on the PHD framework [11]. Whole grains included rice, wheat, and products containing whole grain components. Vegetables and fruits included fresh, frozen, cooked, canned, and dried forms, excluding juices. Dairy comprised milk (whole or skimmed), cheese, and yoghurt, excluding butter and cream. Red meat included unprocessed and processed meats (e.g., beef, pork, lamb). Fish comprised all fish and shellfish. Eggs and poultry included chickens, ducks, and geese. Legumes encompassed beans, lentils, and soybeans. Nuts included tree nuts and groundnuts (e.g., peanuts). Unsaturated oils comprised plant fats (e.g., olive, rapeseed, sunflower oils and margarines), and saturated fats included dairy fats, tallow, and palm oil. Added sugars in foods and beverages were quantified and defined as all sugars added during processing or preparation, excluding intrinsic sugars naturally present in foods. Sugar-sweetened beverages were considered as sources of added sugars. Detailed definitions are available elsewhere [35].

To estimate the composition of mixed dishes with multiple ingredients, a standardised recipe calculation method was consistently applied across all the national surveys [36]. A harmonised recipe database was developed to ensure cross-country comparison. Rather than disaggregating dishes into individual ingredients, standardised formulations were created at the food group level for commonly consumed foods (e.g., stews, pizzas, sandwiches). Where multiple versions of a dish existed (e.g., fish-, red meat-, poultry-, or vegetable-based), each was represented by a separate, standardised recipe reflecting typical preparation. Recipe information was compiled from recipe databases, food labels, and culinary websites [35]. Using these sources, we estimated the proportion of each food group within the dish; for example, a ham and vegetable sandwich with black bread may contained 20% red meat, 10% vegetables, and 50% whole grains. Each recipe was broken down into food group components according to the PHD categories. To quantify food group intake, the total reported weight of each dish consumed was distributed among the food groups based on standardised proportions, converting dish weight into grams per food group. For instance, if an individual reported consuming 200 g of the ham and vegetable sandwich with black bread, the intake was calculated as 40 g red meat (20%), 20 g vegetables (10%), and 100 g whole grains (50%). A total of 141 standardised recipes were included in the final database (Supplementary Material: Table S1).

Importantly, the allocation of ingredients to food groups was guided by both their presence and nutritional value. Foods considered healthy, such as whole grains, were not included in their corresponding group if consumed as part of an unhealthy preparation. For example, a whole grain breakfast cereal with added sugars was considered only for “added sugars” and not as a source of “whole grains”, to remain consistent with the PHD framework [10].

## Data harmonisation

A standardised harmonisation protocol was implemented to ensure consistency across datasets and enable comparative analysis [37, 38]. Participant and dietary data at the individual level were extracted using a pre-defined codebook and a uniform data collection template. Variables from each dataset were recoded and described according to harmonised definitions to ensure alignment across countries. Each data entry captured the food group intake (g/d, “as consumed”), nutrient intake, and participant data. Individual-level microdata were aggregated into subgroups stratified jointly by age (18–44, 45–64, or ≥ 65 years), sex (women or men), and education level (lower or higher, defined by post-secondary education) [38].

Data quality was systematically monitored throughout the process, and any irregularities (i.e., implausible values or structural inconsistencies) were reviewed and resolved collaboratively by the research team [37]. To account for age- and sex-related differences, food intake was standardised to grams per 2500 kcal [38]. Participants reporting extreme energy intakes (< 800 or > 4200 kcal/day for men; <600 or > 3500 kcal/day for women) on the dietary recalls were excluded (*n* = 256; Supplementary Material: Table S2) [39]. Survey weights were calculated separately for each national dataset. For this, the age- and sex-distribution of the survey sample was compared with the corresponding national population distribution obtained from Eurostat. Each participant was assigned a weight proportional to the under- or over-representation of their age-sex stratum in the survey. For the pooled European analysis, these national weights were applied so that each country contributed to the results in proportion to its national population, resulting in a population-weighted European mean.

## PHD adherence assessment

Adherence to the PHD was assessed using two complementary approaches to provide both a detailed and integrative evaluation of diets: compliance with recommended targets for individual food groups, quantifying the proportion of deviation from target intakes, and PHD-based composite indices, summarizing overall diet quality and capturing variability in adherence across food groups.

First, to evaluate compliance with the PHD, we calculated for each food group the percentage of the recommended target intake achieved, defined as % = daily intake/PHD target × 100 [40]. A value of 100% indicates full adherence, > 100% indicates consumption above the target, and < 100% indicates consumption below the target. This allowed the identification of over- and under-consumed food groups in each country relative to the PHD benchmarks (Supplementary Table S3).

In addition, three composite indices were employed to provide a more integrative evaluation of dietary patterns. These indices differ in terms of scoring systems, energy adjustment, treatment of food categories, and thresholds, offering complementary perspectives [35]:

- The World Index for Sustainability and Health (WISH) assesses diet across 13 food groups classified as neutral, protective, or harmful to human and planetary health [41]. Food groups are scored from 0 (noncompliance) to 10 (full compliance) based on reference intakes (g) reflecting adherence to the PHD. The total score ranges from 0 to 130. Further details on WISH are described in Supplementary Material (Table S4) and elsewhere [41].
- The EAT-Lancet Index (ELI) is composed of 14 food groups divided into two categories: seven positive components or “emphasised foods” and seven negative components or “limited foods” [42]. Each component is scored on a graded scale from 0 (noncompliance) to 3 points (high compliance), based on how closely intake aligns with the targets. Total ELI score ranges from 0 to 42. More details on ELI are available in Supplementary Material (Table S5) and elsewhere [42].
- The EAT-Lancet Diet Index (ELD-I) measures how closely a diet al.igns with the PHD across 14 food groups using proportional scoring adjusted for individual energy intake (2500 kcal reference) [43]. Recommended foods score positively when intake exceeds targets; foods to limit score positively when consumption is below limits. Underconsumption of recommended foods or excess intake of limited foods yields negative scores. The resulting unbounded continuous score (positive or negative) reflects the overall adherence. Further details on ELD-I are provided in Supplementary Material (Table S6) and elsewhere [43].

For each index, the contribution of individual food groups to the total score was calculated, enabling the identification of components driving higher or lower adherence within countries. The reliability and validity of the WISH, ELI, and ELD-I in capturing the nutritional health and environmental impacts of diets have been previously established [35].

### Statistical analysis

Descriptive statistics were used to summarize the sample characteristics and dietary intake. Continuous variables were presented as mean ± standard deviation, and categorical variables as frequencies. Food consumption and adherence to the PHD were described and compared across countries and demographic subgroups. Between-country heterogeneity for food groups and PHD indices was assessed using a random-effects meta-analytic framework, providing I^2^ statistics and corresponding plots. For descriptive purposes, the percentage deviation of each food group from the pooled European mean intake was calculated, and intercept-only models were used to test whether national values deviated significantly from the European mean. Additionally, a general dominance analysis was applied to quantify the relative importance of food groups in explaining variation in the PHD indices by decomposing the model R^2^ into dominance weights derived from partial R^2^ contributions across all subset models [44].

Within-country differences in WISH, ELI, and ELD-I according to demographics were evaluated using multivariate regressions, with results expressed as standardised beta coefficients (β) and 95% confidence intervals. Effect sizes were expressed as eta-squared coefficients (η^2^), indicating the proportion of total score variance attributable to each variable, with cut-off points of 0.01 for a small effect, 0.06 for a moderate effect, and 0.14 for a large effect. To account for variation in total energy intake, food group intakes and indices were standardised to 2500 kcal. This adjustment was applied consistently across descriptive and regression analyses. Analyses were performed using RStudio (vR4.5.0, RStudio Team) and Stata (v18, StataCorp, College Station, TX, USA), accounting for sampling weights and considering *p* < 0.05 (two-sided) as statistically significant.

## Results

### Baseline characteristics

The study included 16,083 adults from nine European countries, with sample sizes ranging from 519 in the UK to 3703 in Portugal. Sociodemographic characteristics varied by country (Table 2). Women represented 56.1% of the sample, with the largest gender imbalance in Estonia. Overall, 39.8% were aged 18–45 years, 35.7% were 45–65 years, and 24.6% were 65–80 years. Young and middle-aged adults predominated across most countries, except in Hungary, where over half of the participants were aged 65–80 years. In total, 59.4% had lower education and 40.7% had higher education, ranging from a predominance of lower education in Portugal and Hungary to higher education in Estonia. Sampling weights were applied to adjust for cross-country differences, with the weighted proportions shown in Supplementary Material (Tables S7 and S8).

**Table 2:** Baseline characteristics of participants

Country		Sex		Age			Education	
	Total	Women	Men	18–44	45–64	65–80	Lower	Higher
Europe	16,083	9025 (56.1)	7058 (43.9)	6397 (39.8)	5738 (35.7)	3948 (24.5)	8155 (59.3)	5586 (40.7)
United Kingdom	519	301 (58.00)	218 (42.00)	193 (37.2)	195 (37.6)	131 (25.2)	n/a	n/a
France	2074	1217 (58.7)	857 (41.3)	761 (36.7)	809 (39.0)	504 (24.3)	1214 (58.5)	860 (41.5)
Spain	929	531 (57.2)	398 (42.8)	449 (48.3)	217 (23.4)	263 (28.3)	547 (58.9)	382 (41.1)
The Netherlands	1733	860 (49.6)	873 (50.4)	433 (25.0)	695 (40.1)	605 (34.9)	949 (55.1)	773 (44.9)
Portugal	3703	1970 (53.2)	1733 (46.8)	1730 (46.7)	1322 (35.7)	651 (17.6)	2832 (76.5)	869 (23.5)
Hungary	1032	521 (50.5)	511 (49.5)	236 (22.9)	272 (26.4)	524 (50.8)	489 (69.0)	220 (31.0)
Switzerland	2013	1115 (55.4)	898 (44.6)	903 (44.9)	776 (38.6)	334 (16.6)	1033 (51.4)	977 (48.6)
Finland	1481	778 (52.5)	703 (47.5)	508 (34.3)	553 (37.3)	420 (28.4)	n/a	n/a
Estonia	2599	1732 (66.6)	867 (33.4)	1184 (45.6)	899 (34.6)	516 (19.9)	1092 (42.1)	1505 (57.9)

Values represent counts, with percentages shown in parentheses. n/a = Data not available in the survey

## Exploring PHD food consumption patterns

In the pooled European sample, the mean daily intakes of plant-based foods standardised to 2500 kcal were: 189.6 ± 24.6 g/d for vegetables and 177.1 ± 47.7 g/d for fruits, followed by 66.2 ± 13.6 g/d for tubers, 31.5 ± 19.5 g/d for whole grains, 27.5 ± 12.8 g/d for legumes, 5.2 ± 3.2 g/d for nuts, and 14.0 ± 7.4 g/d for unsaturated oils. For animal-based foods, mean intakes were 263.8 ± 85.6 g/d for dairy, 82.5 ± 10.5 g/d for total red meat (40.4 g/d from beef and lamb and 42.0 g/d from pork), 49.9 ± 17.8 g/d for poultry, 22.8 ± 7.0 g/d for eggs, 38.6 ± 17.4 g/d for fish and seafood, and 28.2 ± 9.3 g/d for saturated fats. The mean intake of added sugars was 54.6 ± 9.5 g/d. Heterogeneity across countries was high for all food groups, with I² values above 95% (*p* < 0.001), as illustrated in the Supplementary Material (Figs. S2 and S3).

**Fig. 1:**
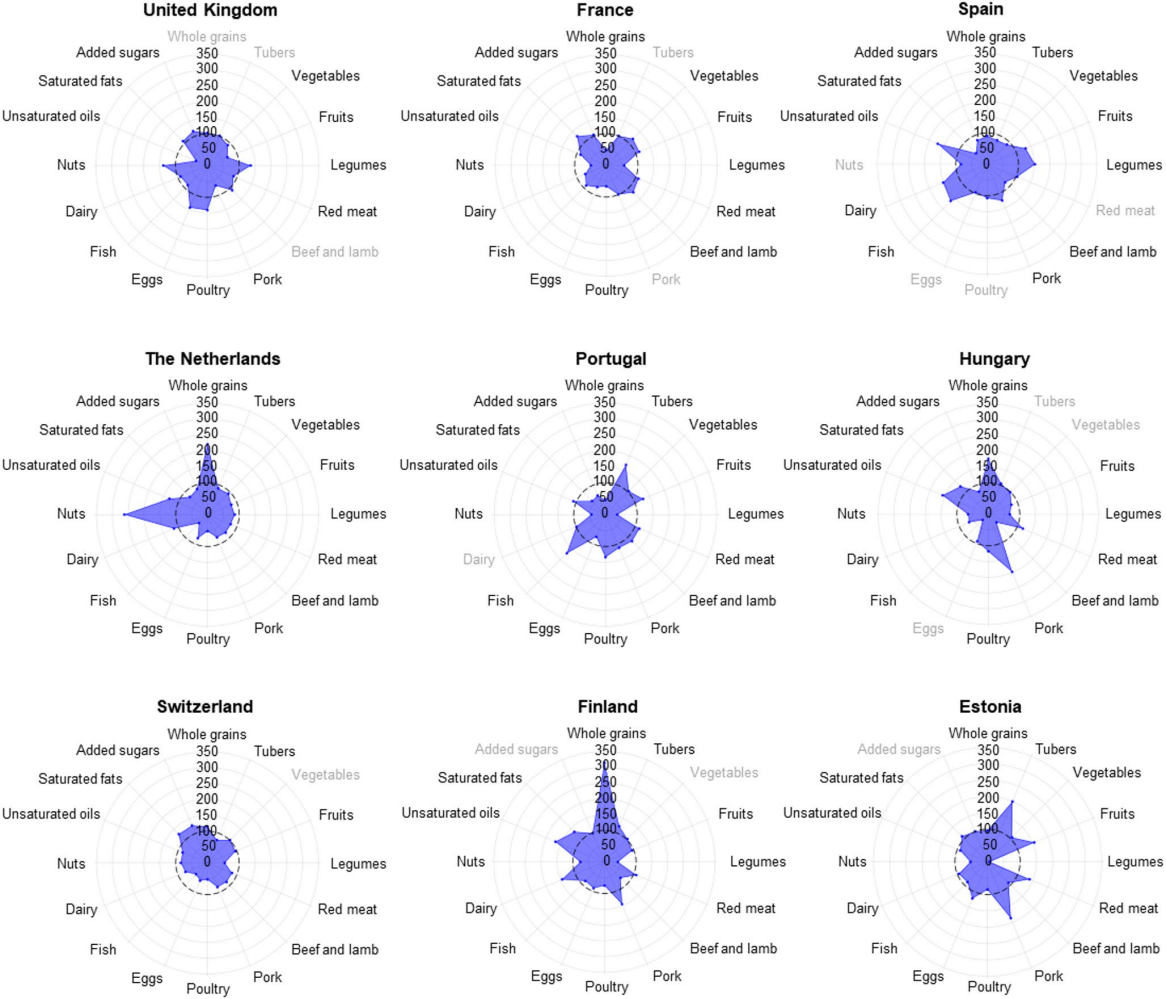
Deviation (%) of the daily individual food consumption from the overall aggregated mean in Europe. The dashed reference circle represents the European average (100%). Labels in grey indicate not significant deviation (*p* > 0.05)

Figure 1 shows country-specific deviations from the European mean for different food groups. Whole-grain consumption was above average in Finland, the Netherlands, and Hungary, but below average in France and Portugal. Nut intake was highest in the Netherlands and lower in France, Portugal, and Estonia. Unsaturated oils were consumed more in Spain, Hungary, and Finland, whereas the UK, France, and Switzerland had lower intake; these countries, in contrast, showed the highest consumption of saturated fats. Fish consumption exceeded the mean in Spain and Portugal, while the Netherlands, Switzerland, and Hungary reported lower values. Legume intake was generally below the mean in Estonia, Switzerland, France, and Portugal, with higher consumption only in the UK and Spain. Estonia also had the highest intake of red meat and tubers relative to the European mean. Switzerland stood out for having added sugar intake above the European mean, whereas Portugal had the lowest consumption. Full deviation magnitudes and significance tests are presented in Fig. 1.

## PHD adherence

**Fig. 2:**
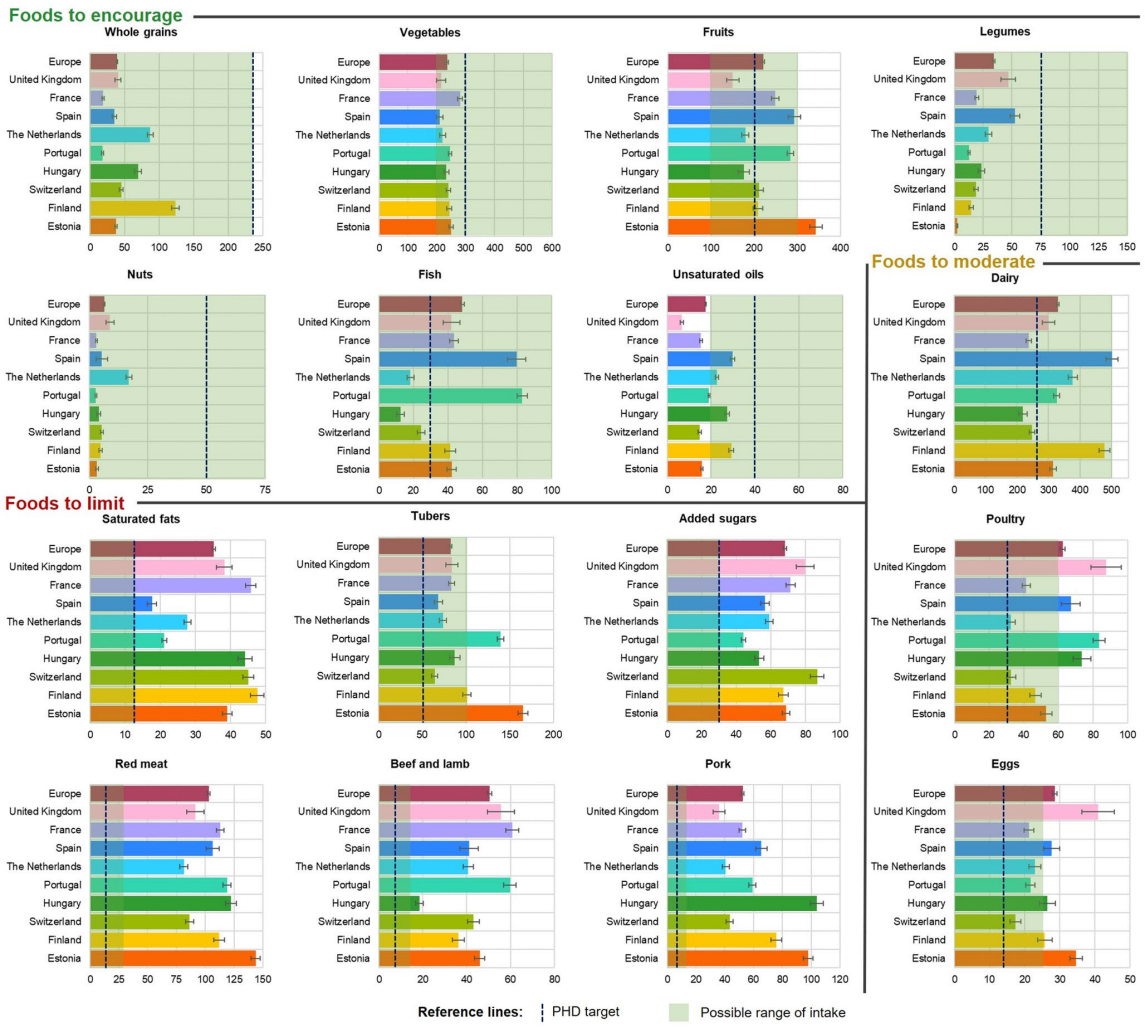
Alignment with the Planetary Health Diet targets across nine European countries. Bars show the average national intake per food group (g/d, standardised to 2500 kcal) with the 95% confidence intervals. The dash line is the PHD target, the green area denotes the possible intake recommended range

Figure 2 illustrates the alignment of mean dietary intakes with the PHD reference values. No country achieved the recommended target for whole grains (17.9–123.8 g/d), nuts (2.86–16.9 g/d), legumes (2.33–52.5 g/d), unsaturated oils (6.7–29.8 g/d), or vegetables (210.4–280.8 g/d). Fruit consumption was also below the target in the UK, the Netherlands, and Hungary (150.8–179.6 g/d), while fish intake was insufficient in the Netherlands, Hungary, and Switzerland (12.4–24.5 g/d). Conversely, poultry consumption exceeded the PHD target in all countries, however it was above the maximum level only in UK, Spain, Portugal and Hungary (67.1–87.6 g/d), with eggs consumption having a similar pattern (exceeding the maximum level in UK, Spain, Hungary, and Portugal: 26.5–40.9 g/d). Red meat consumption exhibited the most significant excess, ranging from 81.4 g/d in the Netherlands to 143.8 g/d in Estonia. In Hungary (103.9 g/d), Estonia (97.9 g/d), Spain (65.5 g/d), Finland (75.9 g/d), and Portugal (59.1 g/d), pork was the primary contributor, whereas beef and lamb were more prominent in France (60.8 g/d vs. 52.1 for pork) and the UK (55.7 vs. 35.9 for pork); Switzerland and the Netherlands reported nearly equal proportions (∼40 g/d). Saturated fat intake ranged from 17.7 g/d in Spain to 47.6 g/d in Finland, while added sugars ranged from 44.1 g/d in Portugal to 86.7 g/d in Switzerland. Tuber consumption was above the target in most countries, but was higher than the maximum recommended level in Estonia (165.1 g/d), Portugal (139.4 g/d), and Finland (101.3 g/d). Further details on food consumption by sex and age can be found in the Supplementary Material (Tables S9 and S10). Additionally, Fig. S4 (Supplementary Material) quantifies these deviations as percentages of the PHD targets, showing that intakes of whole grains, legumes, nuts, vegetables, and unsaturated oils generally reached < 50% of recommended levels, whereas red meat, saturated fats, and added sugars exceeded targets by several-fold in most countries.

The European mean WISH score was 42.03 ± 17.15, highest in Spain (49.79 ± 16.79), the Netherlands (47.68 ± 17.06), and Portugal (45.91 ± 16.83), and lowest in Hungary (39.77 ± 14.99), the UK (37.76 ± 17.86), and Estonia (38.76 ± 14.29). ELI followed a similar pattern (European mean 18.68 ± 5.08), with Spain (20.92 ± 4.58), the Netherlands (19.86 ± 4.74), and Portugal (19.51 ± 4.57) having the highest score, and Estonia (16.46 ± 4.31), the UK (17.06 ± 5.72), and Hungary (18.38 ± 4.18) having the lowest. ELD-I averaged − 14.62 ± 41.15 across Europe; the Netherlands scored positively (3.52 ± 35.70), followed by Spain (− 4.62 ± 37.69) and Portugal (− 6.22 ± 42.15), while the UK (− 27.06 ± 40.91), Finland (− 26.38 ± 42.81), and Hungary (− 23.07 ± 32.34) deviated most (Supplementary Material, Fig. S5). Heterogeneity across countries was high for the three indices, with I² values above 95% (*p* < 0.001), as illustrated in the Supplementary Material (Fig. S6).

The indices were positively correlated across countries (Supplementary Material: Fig. S7). In general, the strongest association was observed between WISH and ELI, with a correlation of 0.80 in the pooled European sample (range across countries: 0.73–0.85) and a quintile agreement of 48% (range: 41–50%). Correlations between ELDI and ELI were moderate (*r* = 0.66; country range: 0.56–0.70) with 40% quintile agreement (range: 34–40%), while WISH and ELDI showed slightly lower correlations (*r* = 0.56; country range: 0.46–0.62) and 36% quintile agreement (range: 32–40%).

**Fig. 3:**
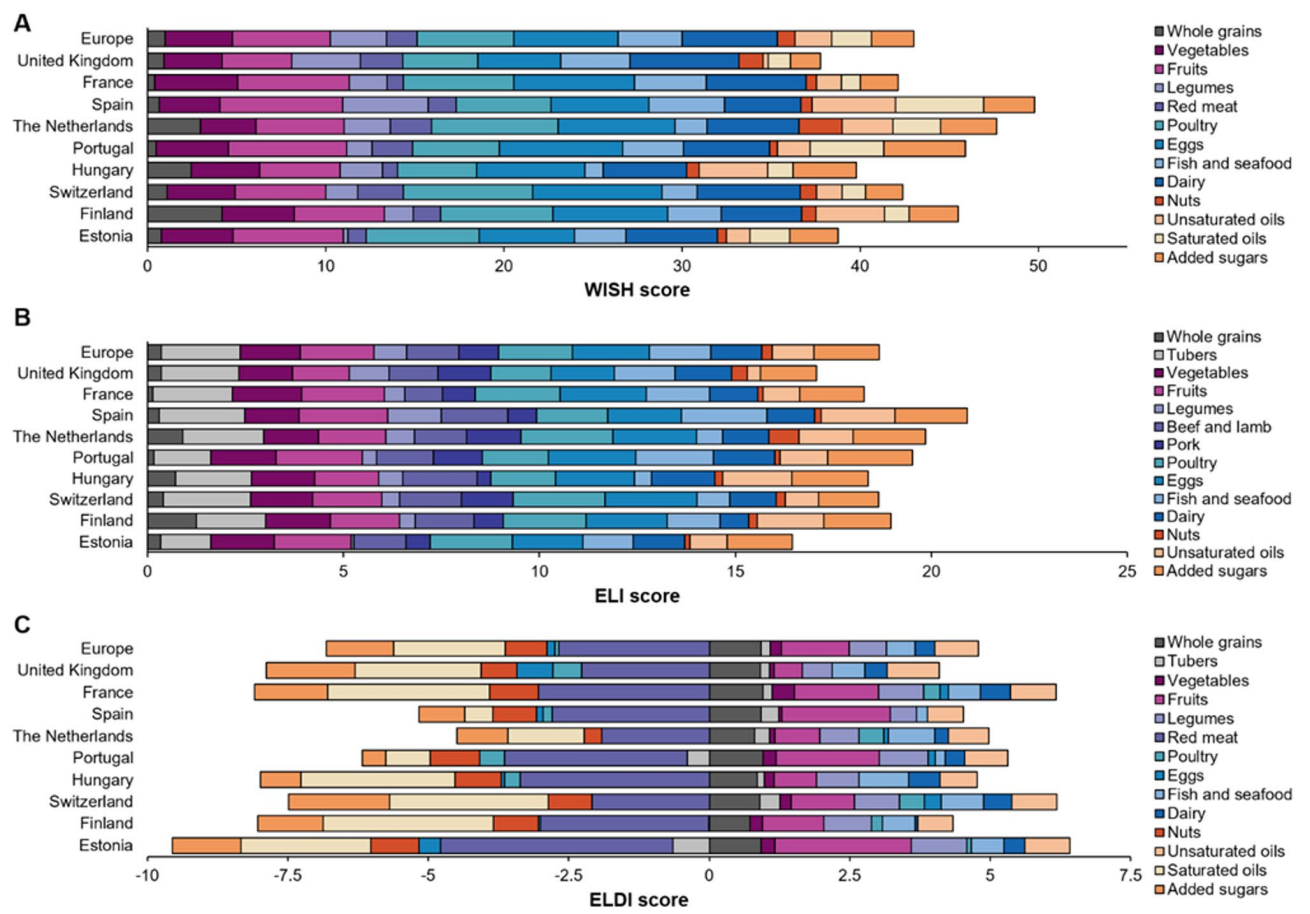
Planetary Health Diet indices in European countries:** A** World Index for Sustainability and Health (WISH),** B** EAT–Lancet Index (ELI),** C** EAT–Lancet Diet Index (ELD-I)

Figure 3 shows the mean food component scores of the dietary indices measuring adherence to PHD. Across Europe, adherence was highest for eggs, dairy, and poultry, particularly in WISH and ELI. Eggs approached the WISH maximum (10 points) in Switzerland (7.25), Portugal (6.94), and France (6.78; European mean 5.86), whilst dairy and poultry averaged 5.35 and 5.43, exceeding 7 points in Switzerland and the Netherlands. In ELI, eggs and poultry scored 1.96 and 1.89 out of 3. Tubers and fruits have similar scores: 2.01 and 1.88, respectively. In ELD-I, fruit contributed most positively (0.51 UK to 2.43 Estonia), along with whole grains, tubers, vegetables, legumes, fish, dairy, and unsaturated oils. Negative ELD-I contributions were obtained for poultry (UK, Spain, Portugal, and Hungary), tubers (Estonia and Portugal), and eggs (UK, Spain, Hungary and Estonia).

The lowest WISH and ELI scores were observed for whole grains (respectively 1.01 and 0.36 in Europe, from 0.38 to 0.14 in France to 4.19 and 1.25 in Finland), legumes (respectively 3.15 and 0.84 in Europe, from 0.27 to 0.07 in Estonia to 4.80 and 1.36 in Spain), and nuts (respectively 0.96 and 0.27 in Europe, from 0.43 to 0.12 in Portugal to 2.43 and 0.78 in the Netherlands). Red meat was the main negative contributor to all indices (WISH: 0.85 in Hungary–2.55 in Switzerland; ELD-I: −1.91 in the Netherlands and − 4.13 in Estonia). In ELI, beef and lamb (1.32) ranged from 0.96 (France) to 1.90 (Hungary), pork (1.01) from 0.61 (Estonia) to 1.38 (the Netherlands). Saturated fats and added sugars also strongly lowered ELD-I. Fig. S8 in Supplementary Material provides a clear visualisation of the food components with the lowest and highest scores for each PHD index across countries. Regarding the dominance analysis (Tables S11–S13 and Figs. S9–S11 in Supplementary Material), across countries, WISH showed a clear dominance of plant-based foods, particularly vegetables and fruits, while animal-based foods and added sugars consistently display low dominance. ELD-I had high dominance for fruits, saturated fats, added sugars, and red meat, reflecting the large weights of these food groups on the score. The ELI presented a mixed pattern, with vegetables, fruits, legumes, and fish showing the highest dominance, and dairy, saturated fats, and most animal-based foods ranking consistently low across countries.

### Sociodemographic characteristics associated with PHD adherence

**Fig. 4:**
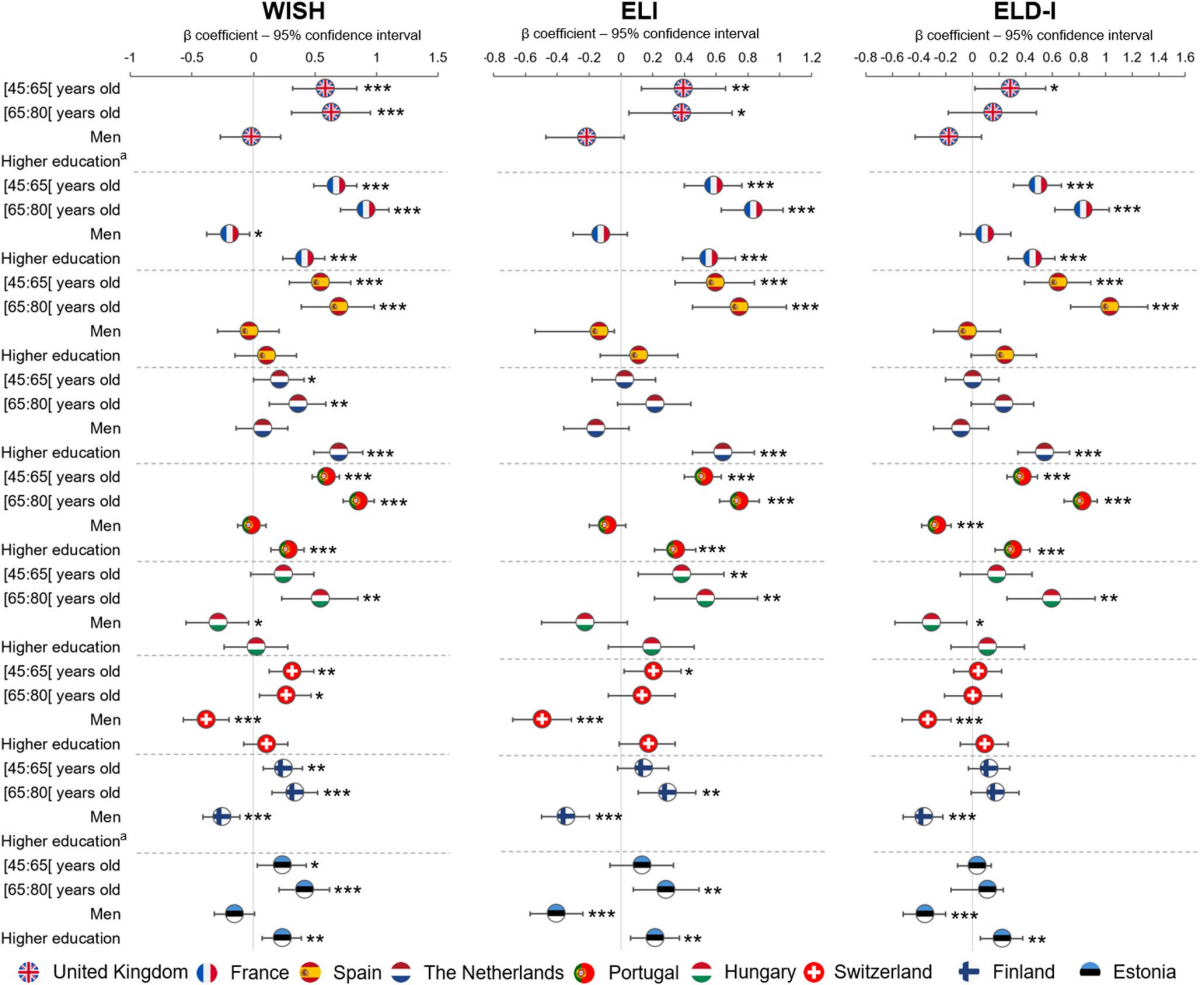
Associations between sociodemographic factors and Planetary Health Diet indices in nine European countries: World Index for Sustainability and Health (WISH), EAT–Lancet Index (ELI), EAT–Lancet Diet Index (ELD-I). Results derive from separate regression models fitted independently for each country. Coefficients (β) and 95% confidence intervals are shown. The reference groups were adults aged 18–44 years, women, and individuals with lower education. ^a^No educational data were available for the United Kingdom or Finland. Indices were energy-standardised to account for differences in total intake. Interactions between sociodemographic factors were included in the regression models. *p < 0.05; **p < 0.001; ***p < 0.0001

Within each country, associations between sociodemographic factors and PHD adherence were generally consistent across the three indices (WISH, ELI, and ELD-I), indicating robust patterns regardless of the specific adherence measure used. Figure 4 presents the associations between PHD indices and sociodemographic factors, with France, Portugal, and Estonia showing significant associations for age, sex, and education.

Age showed the largest and most consistent differences in PHD adherence across countries and indices, with older adults scoring higher than younger adults. For example, in France, individuals aged 65–80 scored 13.6 points higher on WISH (95% CI 10.7, 16.6) and 33.9 points higher on ELD-I (95% CI 25.7, 42.2) compared with those aged 18–44 (*p* < 0.0001, moderate effect sizes). Similar age gradients were observed in Spain and Portugal, whereas age-related differences were smaller in Central and Northern European countries.

Sex-related differences varied across countries and indices. In Switzerland and Finland, men consistently scored lower than women across all indices (*p* < 0.0001, small effect sizes). For instance, in Switzerland, men scored 6.4 points lower on WISH (95% CI −9.5, − 3.4; η^2^ = 0.03), while in Finland they scored 15.9 points lower on ELD-I (95% CI −22.3, − 9.5; η^2^ = 0.04). In Estonia, men scored 1.8 points lower on ELI (95% CI −2.5, − 1.1; η^2^ = 0.03) and 19.0 points lower on ELD-I (95% CI −27.5, − 10.4; η^2^ = 0.05), whereas WISH did not vary. In contrast, sex-related differences were smaller in magnitude and less consistent across indices in the other countries.

Across the countries with harmonised education data, higher education level was associated with higher PHD scores in France, the Netherlands, Portugal, and Estonia (*p* < 0.0001). However, the magnitude of these differences was small (η^2^ < 0.04), corresponding to approximately 3–12 points for WISH, 1–3 points for ELI, and 12–19 points for ELD-I. Conversely, indices were similar across education levels in Spain, Switzerland, and Hungary. Additional information is available in the Supplementary Material (Tables S14–S22 and Figs. S12–S14).

## Discussion

This study provides a cross-national assessment of dietary patterns in relation to the PHD across nine European countries. Our findings show large differences between countries and significant sociodemographic variations, but also point to a convergence toward overall low adherence to PHD targets. While some countries had relatively high intakes of vegetables, dairy, and fish, these were counterbalanced by excessive consumption of red meat, saturated fats, and added sugars, as well as insufficient intake of whole grains, legumes, and nuts. In absolute terms, European mean intakes standardised to 2500 kcal underscore the magnitude of these gaps. Average consumption of whole grains (~ 30 g/d; PHD target = 232 g/d), legumes (~ 30 g/d; PHD target = 75 g/d), nuts (~ 5 g/d; PHD target = 50 g/d), and vegetables (~ 240 g/d; PHD target = 300 g/d) were well below of the PHD recommended average targets. In contrast, intakes of foods to balance and to limit exceeded PHD targets markedly, including red meat (~ 100 g/d; PHD target = 28 g/d), added sugars (~ 55 g/d; PHD target = 31 g/d), and saturated fats (~ 35 g/d; PHD target = 11.8 g/d). Notably, most of the countries failed to meet the PHD targets for both encouraged and limited foods , which is consistent with prior evidence from high-income countries [45, 46].

Marked heterogeneity in European dietary patterns was observed, in line with cultural traditions and established nutritional trends. Northern European countries, such as Finland, reported high whole grain consumption, consistent with traditional rye- and oat-based foods previously associated with improved health outcomes [47, 48], whereas intake was low in France, highlighting persistent gaps despite national efforts [49–51]. Legumes and nuts were underconsumed across all countries, in line with previous studies showing that no European country meets recommended targets [23, 52–54]. Mediterranean countries, including Spain and Portugal, demonstrated higher intakes of fish and unsaturated oils, consistent with traditional dietary patterns [55, 56]. Northern and Central European countries relied more on animal fats and red meat, a pattern that has been attributed in previous studies to entrenched dietary habits and socioeconomic factors [55, 57–60]. Although fruit and vegetable intake exceeded global averages [46], most countries still fell short of PHD targets [61, 62], suggesting that public health efforts should continue to prioritize these food groups. Similarly, added sugars and saturated fats were above recommended levels across Europe [63–65], underscoring the need for integrated policy approaches that combine regulatory measures, education, and agricultural strategies [66–68]. Overall, our results align with previous European evidence and highlight persistent challenges in achieving adherence to healthy and sustainable dietary patterns [69–72]. A detailed country- and food-group-specific discussion is provided in Supplementary Material. Composite indices enhanced the dietary assessment by integrating the PHD thresholds and encompassing the multidimensional structure of the diet. Furthermore, prior research indicates that these indices are differentially associated with nutritional and environmental indicators, underscoring their utility in evaluating complementary aspects of PHD [35]. Specifically, proportional scoring-based indices (e.g., WISH) better capture dietary variability and nutritional quality, whereas indices using graded or binary scoring systems (e.g., ELI) tend to correlate more strongly with environmental impact measures [35]. PHD indices that are scored using an unbounded proportional metric, such as ELD‑I, capture both dimensions [35]. Moreover, the dominance analysis helped to interpret how different PHD indices operationalise dietary components. The predominance of plant-based foods in WISH, the stronger contribution of foods to limit in ELD-I, and the mixed pattern observed for ELI reflect differences in index structure and weighting. However, these dominance patterns represent statistical contributions under correlated dietary components and should not be interpreted as causal influence. Taken together, these methodological differences underscore the value of applying multiple indices to evaluate complementary aspects of adherence to the PHD and provide a more robust assessment of dietary patterns.

In this study, composite indices confirmed that overall adherence to the PHD was low. Spain, the Netherlands, and Portugal exhibited the highest scores, while the UK, Hungary, and Estonia had the lowest scores. These findings are consistent with previous country-specific studies. In Spain, the ENRICA study (*n* = 13,105) reported a mean PHD score of 87 (out of 140) [73], whilst the EPIC-NL study in the Netherlands (*n* = 35,496) found an average score of 73 (out of 140) [74]. By contrast, data from the UK Biobank (*n* = 125,372) showed a median PHD score of 59 (out of 110) [75], and a nutrient-based EAT-Lancet score in a study from Hungary (*n* = 359) yielded a median of 2 (out of 12) [76]. Collectively, despite differences in scoring metrics and study designs, these studies reveal patterns consistent with our findings, reinforcing the evidence that adherence to the PHD is generally low in high-income countries [70].

Low alignment with the PHD was characterised by excess red meat, saturated fats, and added sugars, alongside insufficient whole grains, legumes, nuts, and vegetables, in line with previous research [45, 70]. Eggs, poultry, and dairy intakes were relatively consistent across countries. The Netherlands stood out in terms of whole grain and nut consumption, while Spain led in fish, legume, and unsaturated fat consumption, which may reflect the closer alignment of the Mediterranean diet with the PHD [70, 77]. Previous research, using a different PHD index from those applied in the present study, has shown that modest adherence to PHD dietary patterns in high-income countries was largely driven by excess red meat and added sugars [45]. In Central and Eastern European countries, such as Hungary and Estonia, tuber overconsumption was an additional contributing factor. Over the period 1990–2018, the greatest improvements in this alternative PHD index were observed in high-income countries (+ 6.5 points), largely due to higher component scores for tubers and nuts, with the Netherlands showing the largest gains [45].

Across the nine European countries studied, age was the most consistent predictor of higher adherence to the PHD, with older adults scoring significantly higher than younger adults, particularly in France, Spain, and Portugal. These findings are consistent with previous research showing healthier dietary patterns and lower consumption of ultra-processed food and meat among older adults in Europe, including Spain [78, 79], Switzerland [80, 81], the UK [82], Portugal [83], and Finland [84, 85]. Older age has also been linked to greater vegetable and fruit intakes [79, 86] and lower fast food and (processed) meat consumption [78, 87]. Previous studies have linked these differences to greater cooking skills, more time for food preparation, adherence to traditional diets, generational norms, increased health consciousness, and greater disposable income to spend on healthier foods [79].

Sex differences were more variable across countries, although women tended to score higher on the PHD indices. This aligns with the literature indicating that women report healthier and more plant-forward diets, including lower red and processed meat intake and higher fruit and vegetable consumption [79, 88–90]. Similar sex-related differences have been observed for sustainable dietary indices [88–92] and for specific components, such as vegetable and seafood [85, 93]. This pattern is especially pronounced in Baltic and Nordic regions, such as Estonia, where men’s higher red and processed meat intakes drive the gap [84, 94, 95]. Health consciousness, environmental awareness, and willingness to change dietary habits may explain women’s higher scores, whereas cultural norms and men’s preference for energy-dense foods may contribute to lower scores among men [74, 76, 79, 88–90].

Educational disparities appear to be an important correlate of diet in the European countries where education data were available. Regarding this, higher education was associated with better dietary scores, particularly in France, Portugal, the Netherlands, and Estonia, consistent with previous findings linking education to healthier and more sustainable diets, including higher intakes of fruit, vegetable, whole grain, and fish, and lower intakes of red and processed meat [88–90, 96, 97]. In France, higher education correlates with plant-forward diets and higher multidimensional sustainability of diets (i.e., environmental, nutritional, economic and sociocultural dimensions) [88]. Across Europe, lower education levels are often linked to higher processed meat consumption, lower fruit and vegetable intake, and higher ultra-processed food consumption [79, 80, 88, 98]. However, in Spain, Switzerland, and Hungary, educational differences were smaller, reflecting patterns similar to those in previous research [81, 99]. These variations in dietary patterns by education level have been linked to higher income levels, better nutritional awareness, food literacy, health- and environment-focused attitudes, and enhanced access to quality foods among individuals with higher education levels [79, 98].

This study drew on harmonised data from a large and diverse sample of adults across nine European countries, offering a multidimensional perspective on diet quality through three complementary indices. However, some limitations should be acknowledged. The cross-sectional design limits causal inference, and self-reported intake may introduce recall and social desirability bias, especially for foods such as red meat and added sugars; however, 24-hour recalls are generally more accurate than other dietary assessment methods [100]. Another potential limitation relates to the lack of usual intake modelling. Although such methods can reduce within-person variability in 24-hour recall data, the heterogeneity in the number of recall days and the structure of dietary information across surveys did not allow a harmonised application [101, 102]. Given that several food groups were consistently consumed far below or far above recommended levels, the overall impact on our adherence estimates is likely limited [101]. To further assess the robustness of our findings to differences in survey design, we conducted a sensitivity analysis excluding the UK and France, the two countries with a higher number of recall days (Supplementary Material: Figure S15). Results were very similar, and the overall interpretation of shortfalls and excesses relative to PHD targets remained unchanged. Nevertheless, future research should incorporate harmonised usual intake modelling to improve the estimation of intake distributions and better quantify the proportion of populations meeting or exceeding dietary recommendations.

Another methodological consideration relates to the use of foods “as consumed” in the harmonised datasets. While this approach reflects real-world intake more accurately, it also introduces heterogeneity because the use of conversion factors may differ across original national surveys [103]. This may affect comparability for food groups whose weight change markedly with cooking, particularly legumes, whole grains, or meat. In addition, the PHD specifies reference values for legumes in their dry form, whereas intake in our datasets was captured only as consumed. Thus, although we applied standard harmonisation procedures, these transformations may introduce uncertainty in the estimation of adherence. Future research should aim to harmonize conversion factors across surveys and, where possible, collect both raw and cooked weights to align more precisely with PHD recommendations.

On the other hand, national surveys varied in terms of demographic composition and collection periods (e.g., Estonia 2013–2015 vs. UK 2020), affecting comparability. As a result, the country rankings presented here should be interpreted as time-stamped snapshots rather than stable national characteristics. Stronger temporal harmonisation is required to assess the persistence of these patterns over time. Some datasets lacked key variables, such as education in the UK and Finland, limiting the analyses. In addition, only two education levels were used, which was the most feasible approach to harmonize data due to the heterogeneity of education classifications across surveys, which may restrict the ability to draw strong conclusions. Future research should consider additional factors such as income, employment, and urbanisation [76]. Standardised recipes ensured comparability but may not capture regional differences in preparation, portion size, and ingredient quality, potentially misestimating foods that are difficult to quantify, such as added sugars or fats [104]. We encourage future research to explore more detailed assessments of nutrient composition, for instance fat quality (e.g., the ratio of unsaturated to saturated fats), as more comprehensive nutrient data become available. Although we applied weighting for age and sex within each national survey, the variance estimation did not fully reflect the survey design (stratification and clustering of primary sampling units), and replicate weights were not available, which may slightly underestimate variability.

Additionally, a strict recipe-disaggregation rule was applied whereby foods encouraged by the PHD were not credited when consumed together with substantial amounts of foods to limit (e.g., whole-grain products with added sugars or saturated fats). While this approach may result in lower estimated intakes of encouraged foods and influence cross-country comparisons, it reflects an intentional design choice fully aligned with the PHD framework, which also explicitly discourages ultra-processed foods in the 2.0 version [105]. Accordingly, our estimates capture adherence to the quality of encouraged foods rather than the nominal presence of individual ingredients. To contextualize the potential magnitude of this choice, illustrative benchmarks indicate only small differences under broader classifications. In the UK, where reclassification within our data structure was feasible, mean intake increased from 40 g/d under the strict PHD-aligned definition to 54 g/d when all whole-grain products were included irrespective of added sugars. In the Netherlands, the DNFCS survey reports a mean intake of approximately 100 g/d for whole-grain products [30], compared with 87 g/d under the PHD-aligned classification applied in the current study. Even under these estimates, intake levels remain far below the PHD target of 232 g/d, suggesting that the large observed gaps primarily reflect low adherence rather than the specific disaggregation strategy. Our results should therefore be interpreted in light of this conceptual alignment with PHD principles. Finally, another potential limitation is the use of fixed cut-points for extreme energy intakes due to data availability, highlighting the need for future studies to explore alternative approaches to better assess misreporting.

Shifting toward healthier and more sustainable diets is widely recognised as a complex challenge involving cultural, structural, and behavioural dimensions. Our findings highlight substantial shortfalls in legumes, nuts, and whole grains, alongside excess red-meat intake, underscoring the need for policy levers that directly target these gaps. A range of policy instruments, such as fiscal policies (e.g., targeted taxes and subsidies), public procurement standards, product reformulation, and harmonised front-of-pack labelling, have been proposed in the literature as potential avenues to support alignment with dietary recommendations. Recent initiatives, including the EU Farm-to-Fork Strategy, the UK National Food Strategy and UK Agri-food for Net Zero Roadmap, already point in this direction by promoting plant-based proteins, improving labelling, and reducing overconsumption of animal-source foods [106–108]. However, despite strong evidence and alignment with policy priorities, the first two strategies were undermined by corporate and regulatory capture from powerful multinational food corporations and lobby groups [109, 110], helping to contextualize the shortfalls in plant foods and the continued excess consumption of animal products and added sugars observed in our study. While broader systemic factors will continue to shape food-system transformation, these evidence-based levers offer immediate and actionable pathways for addressing the specific dietary imbalances identified in our study. Example multi-level and -sectoral actions that can promote healthier and more sustainable food choice architecture are provided in Supplementary Material (Table S23).

Our findings provide a comprehensive cross-national perspective on adherence to the PHD. The use of PHD indices enabled a multidimensional evaluation of dietary patterns and underscored the influence of sociodemographic factors. Overall, European diets remain misaligned with PHD targets, with legumes, nuts, whole grains and vegetables underconsumed, while red meat, saturated fats and added sugars are overconsumed. Although certain countries exhibit favourable trends in specific food categories, substantial differences persist across countries and sociodemographic groups, underscoring the need for equity-focused and culturally tailored strategies. Importantly, meaningful progress towards healthier and more sustainable diets require not only individual change but also coordinated structural and policy action across governance levels and food system sectors.

## Supplementary Information

Below is the link to the electronic supplementary material.

Supplementary Material 1

## Data Availability

The INCA3, IAN-AF, and RTU datasets are publicly available at: https://www.data.gouv.fr; https://www.ian-af.up.pt, https://globaldietarydatabase.org. Access to the FINDIET, HU-EU-Menu, and ENALIA 2 datasets was requested through the European Food Safety Authority portal (https://www.efsa.europa.eu). For the DNFCS (https://www.rivm.nl/en/dutch-national-food-consumption-survey), menuCH (https://www.studydata.blv.admin.ch/home, and NDNS (https://beta.ukdataservice.ac.uk/datacatalogue/studies/study? id=8956) datasets, access was obtained by directly contacting the respective national data owners.

## References

[1] Mago A, Dhali A, Kumar H, Maity R, Kumar B (2024) Planetary health and its relevance in the modern era: a topical review. SAGE Open Med. 10.1177/20503121241254231 38774741 10.1177/20503121241254231 PMC11107315

[2] Barbero Vignola G et al (2024) Existing sustainability efforts and policies in the food systems in the EU and worldwide. European commission. Joint Research Centre, Publications Office of the European Union, Luxembourg. 10.2760/1278262

[3] van Dooren C et al (2024) The planet on our plates: approaches to incorporate environmental sustainability within food-based dietary guidelines. Front Nutr 2024. 10.3389/fnut.2024.1223814 10.3389/fnut.2024.1223814 PMC11259098 39036493

[4] de Souza CV et al (2025) Following the food consumption footprints of adults and the elderly around the world: a systematic review. Nutr Rev. 10.1093/nutrit/nuaf061 40591516 10.1093/nutrit/nuaf061

[5] Yang Y et al (2024) Climate change exacerbates the environmental impacts of agriculture. Science. 10.1126/science.adn3747 39236181 10.1126/science.adn3747

[6] Grosso G et al (2022) Total, red and processed meat consumption and human health: an umbrella review of observational studies. Int J Food Sci Nutr. 10.1080/09637486.2022.2050996 35291893 10.1080/09637486.2022.2050996

[7] Gillespie KM, Kemps E, White MJ, Bartlett SE (2023) The impact of free sugar on human Health-A narrative review. Nutrients 10.3390/nu15040889 10.3390/nu15040889 PMC9966020 36839247

[8] Parajára MC et al (2019) Mortality attributable to diets low in fruits, vegetables, and whole grains in Brazil in 2019: evidencing regional health inequalities. Public Health. 10.1016/j.puhe.2023.08.028 10.1016/j.puhe.2023.08.028 37774566

[9] Allen TS et al (2025) Red meat consumption and hypertension: an updated review. Curr Cardiol Rep. 10.1007/s11886-025-02201-2 39928063 10.1007/s11886-025-02201-2 PMC11811473

[10] Bonaccio M et al (2021) Ultra-processed food consumption is associated with increased risk of all-cause and cardiovascular mortality in the Moli-sani study. Am J Clin Nutr. 10.1093/ajcn/nqaa299 33333551 10.1093/ajcn/nqaa299

[11] Willett W et al (2019) Food in the anthropocene: the EAT–Lancet commission on healthy diets from sustainable food systems. Lancet. 10.1016/s0140-6736(18)31788-4 30660336 10.1016/S0140-6736(18)31788-4

[12] Fanzo J, Davis C (2021) Sustainable diets: aligning food systems and the environment. Global food Systems, Diets, and nutrition: linking Science, Economics, and policy. Palgrave Macmillan, Cham, pp 155–168. 10.1007/978-3-030-72763-5_10

[13] Pauw K, Ecker O, Thurlow J, Comstock AR (2023) Measuring changes in diet deprivation: new indicators and methods. Food Policy. 10.1016/j.foodpol.2023.102471

[14] Laine JE et al (2021) Co-benefits from sustainable dietary shifts for population and environmental health: an assessment from a large European cohort study. Lancet Planet Health. 10.1016/S2542-5196(21)00250-3 34688354 10.1016/S2542-5196(21)00250-3 PMC8581185

[15] Drewnowski A, Finley J, Hess JM, Ingram J, Miller G, Peters C (2020) Toward healthy diets from sustainable food systems. Curr Dev Nutr 10.1093/cdn/nzaa083 10.1093/cdn/nzaa083 PMC7288378 32551411

[16] European Commission (2025) EU’s Climate Law presents a new way to get to 2040. https://ec.europa.eu/commission/presscorner/detail/en/ip_25_1687. Accessed 8 July 2025

[17] FOAG FSVO, FOEN (2024) Klimastrategie Landwirtschaft und Ernährung 2050 [Climate Strategy Agriculture and Food 2050]. Bern, Switzerland. https://www.blw.admin.ch/de/klimastrategie-landwirtschaft-und-ernaehrung-2050. Accessed on 8 July 2025

[18] Government of the United Kingdom (2023) Powering Up Britain: Net Zero Growth Plan. Policy Paper; UK Government: London, UK. https://www.gov.uk/government/publications/powering-up-britain/powering-up-britain-net-zero-growth-plan. Accessed on 8 July 2025

[19] Sacramento-Pacheco J, Sánchez-Gómez MB, Duarte-Clíments G, Gómez-Salgado J, Novo-Muñoz MM (2025) Prevalence of cardiovascular risk factors among adults in the European union: a systematic review with Meta-Analysis. J Clin Med. 10.3390/jcm14165752 40869583 10.3390/jcm14165752 PMC12387011

[20] Jani A et al (2022) Transitions to food democracy through multilevel governance. Front Sustain Food Syst. 10.3389/fsufs.2022.1039127

[21] Alves R, Perelman J, Chang K, Millett C (2024) Environmental impact of dietary patterns in 10 European countries; a cross-sectional analysis of nationally representative dietary surveys. Eur J Public Health. 10.1093/eurpub/ckae088 38776529 10.1093/eurpub/ckae088 PMC11430961

[22] Mertens E et al (2021) Improving health and carbon footprints of European diets using a benchmarking approach. Public Health Nutr. 10.1017/S1368980020003341 32962783 10.1017/S1368980020003341 PMC7844616

[23] Daas MC et al (2025) Diversity of dietary protein patterns across Europe - Impact on nutritional quality and environmental sustainability. Curr Res Food Sci. 10.1016/j.crfs.2025.101019 40151663 10.1016/j.crfs.2025.101019 PMC11946498

[24] Işıkgöz ME, Arslan MA (2025) Study on the clustering of daily fruit and vegetable consumption by educational level in European region countries. Food Sci Nutr 10.1002/fsn3.70510 10.1002/fsn3.70510 PMC12203402 40585497

[25] Grant F, Aureli V, Di Veroli JN, Rossi L (2025) Mapping of the adherence to the planetary health diet in 11 European countries: comparison of different diet quality indices as a result of the PLAN’EAT project. Front Nutr. 10.3389/fnut.2025.1645824 41041127 10.3389/fnut.2025.1645824 PMC12486606

[26] National Institute for Health Development (2014) The Estonian National Dietary Survey (RTU) 2014. https://statistika.tai.ee/Resources/PX/Databases/Andmebaas/05Uuringud/09RTU/b_Toidud_nadal/RTUinfo_en.htm

[27] Kaartinen N et al (2020) The Finnish National dietary survey in adults and elderly (FinDiet 2017). EFSA Support Publ 10.2903/sp.efsa.2020.EN-1914

[28] Dubuisson C, Dufour A, Carrillo S, Drouillet-Pinard P, Havard S, Volatier JL (2019) The third French individual and National food consumption (INCA3) survey 2014–2015: method, design and participation rate in the framework of a European harmonization process. Public Health Nutr. 10.1017/S1368980018002896 30394264 10.1017/S1368980018002896 PMC10260437

[29] Csizmadia K, Larnsak L, Pfaff N, Sali J (2020) Hungarian National food consumption survey on adults. 10.2903/Sp. .Efsa.2020.En-1981 EFSA Support Publ

[30] van Rossum C et al (2023) The diet of the Dutch. Results of the Dutch National Food Consumption Survey 2019–2021 on Food Consumption and evaluation with dietary guidelines. RIVM, Available from: https://rivm.openrepository.com/entities/publication/3c44b111-e388-49c3-81cb-74e033a012fe

[31] Lopes C et al (2018) National Food, Nutrition, and physical activity survey of the Portuguese general population (2015–2016): protocol for design and development. JMIR Res Protoc10.2196/resprot.8990 10.2196/resprot.8990 PMC5832902 29449204

[32] Marcos Suarez V, Rubio Mañas J, Sanchidrián Fernández R (2016) Spanish National dietary survey in adults, elderly and pregnant women. EFSA Support Publ 10.2903/sp.efsa.2016.EN-1053

[33] Chatelan A et al (2017) Major differences in diet across three linguistic regions of switzerland: results from the first National nutrition survey menuch. Nutrients 10.3390/nu9111163 10.3390/nu9111163 PMC5707635 29068399

[34] Venables MC et al (2022) Data resource profile: united Kingdom National diet and nutrition survey rolling programme (2008-19). Int J Epidemiol. 10.1093/ije/dyac106 35640141 10.1093/ije/dyac106 PMC9365634

[35] Miranda AR, Vieux F, Maillot M, Verger EO (2025) How do the indices based on the EAT-Lancet recommendations measure adherence to healthy and sustainable diets? A comparison of measurement performance in adults from a French National survey. Curr Dev Nutr 10.1016/j.cdnut.2025.104565 10.1016/j.cdnut.2025.104565 PMC11919322 40104607

[36] Vossenaar M et al (2022) Guidance for the Use of Standard and Non-Standard Recipes in Quantitative 24-Hour Dietary Recall Surveys: The Simple Ingredient Method. Intake-Center for Dietary Assessment/FHI Solutions, Washington. https://www.intake.org/sites/default/files/2023-01/A%20guidance%20document%20for%20recipes%20-%20final%203%20jan%202023.pdf

[37] Karageorgou D et al (2024) Harmonising dietary datasets for global surveillance: methods and findings from the global dietary database. Public Health Nutr. 10.1017/S1368980024000211 38238892 10.1017/S1368980024000211 PMC10882534

[38] Miller V et al (2021) Global dietary database 2017: data availability and gaps on 54 major foods, beverages and nutrients among 5.6 million children and adults from 1220 surveys worldwide. BMJ Glob Health. 10.1136/bmjgh-2020-003585 33547175 10.1136/bmjgh-2020-003585 PMC7871251

[39] Subar AF et al (2001) Comparative validation of the Block, Willett, and National cancer Institute food frequency questionnaires: the eating at america’s table study. Am J Epidemiol. 10.1093/aje/154.12.1089 11744511 10.1093/aje/154.12.1089

[40] Hendrie GA et al (2022) Towards healthier and more sustainable diets in the Australian context: comparison of current diets with the Australian dietary guidelines and the EAT-Lancet planetary health diet. BMC Public Health. 10.1186/s12889-022-14252-z 36261800 10.1186/s12889-022-14252-z PMC9583557

[41] Trijsburg L et al (2020) Method for the development of WISH, a globally applicable index for healthy diets from sustainable food systems. Nutrients 10.3390/nu13010093 10.3390/nu13010093 PMC7824146 33396659

[42] Stubbendorff A, Sonestedt E, Ramne S, Drake I, Hallström E, Ericson U (2022) Development of an EAT-Lancet index and its relation to mortality in a Swedish population. Am J Clin Nutr. 10.1093/ajcn/nqab369 34791011 10.1093/ajcn/nqab369 PMC8895215

[43] Kesse-Guyot E et al (2021) Environmental and nutritional analysis of the EAT-Lancet diet at the individual level: insights from the NutriNet-Santé study. J Clean Prod. 10.1016/j.jclepro.2021.126555

[44] Mustafa A, Shekhar C (2023) Factors associated with vitamin D deficiency and their relative importance among Indian adolescents: an application of dominance analysis. Int J Endocrinol. 10.1155/2023/4209369 37881405 10.1155/2023/4209369 PMC10597726

[45] Li Z et al (2025) Adherence to the modified planetary health diet among the working-age population in 185 countries from 1990 to 2018: a population-based study. Am J Clin Nutr. 10.1016/j.ajcnut.2025.06.016 40545002 10.1016/j.ajcnut.2025.06.016

[46] Micha R, Khatibzadeh S, Shi P, Andrews KG, Engell RE, Mozaffarian D (2015) Global, regional and National consumption of major food groups in 1990 and 2010: a systematic analysis including 266 country-specific nutrition surveys worldwide. BMJ Open. 10.1136/bmjopen-2015-008705 26408285 10.1136/bmjopen-2015-008705 PMC4593162

[47] Price EJ, Barrett EM, Batterham MJ, Beck EJ (2024) Exploring the reporting, intake and recommendations of primary food sources of whole grains globally: a scoping review. Br J Nutr. 10.1017/S0007114524002678 39494733 10.1017/S0007114524002678 PMC11646673

[48] Tammi R, Männistö S, Maukonen M, Kaartinen NE (2024) Whole grain intake, diet quality and risk factors of chronic diseases: results from a population-based study in Finnish adults. Eur J Nutr. 10.1007/s00394-023-03272-z 37934237 10.1007/s00394-023-03272-z PMC10899358

[49] Bellisle F, Hébel P, Colin J, Reyé B, Hopkins S (2014) Consumption of whole grains in French children, adolescents and adults. Br J Nutr. 10.1017/S0007114514002670 25300424 10.1017/S0007114514002670 PMC4234471

[50] Langmann F et al (2025) Plant-based diets, legumes, and prevalence of cardiometabolic risk factors in the NutriNet-Santé cohort. Eur J Nutr. 10.1007/s00394-025-03722-w 40445244 10.1007/s00394-025-03722-w

[51] Fassier P, Rabès A, Ducrot P, Serry AJ (2023) Impact of a French social marketing campaign promoting pulse and whole grain consumption: results from a longitudinal cohort study. Front Nutr. 10.3389/fnut.2023.1208824 37614747 10.3389/fnut.2023.1208824 PMC10442484

[52] Hughes J, Pearson E, Grafenauer S (2022) Legumes-A comprehensive exploration of global Food-Based dietary guidelines and consumption. Nutrients. 10.3390/nu14153080 35956258 10.3390/nu14153080 PMC9370574

[53] Henn K, Goddyn H, Olsen SB, Bredie WL (2022) Identifying behavioral and attitudinal barriers and drivers to promote consumption of pulses: a quantitative survey across five European countries. Food Qual Prefer 10.1016/j.foodqual.2021.104455

[54] Jenab M et al (2006) Consumption and portion sizes of tree nuts, peanuts and seeds in the European prospective investigation into cancer and nutrition (EPIC) cohorts from 10 European countries. Br J Nutr. 10.1017/bjn20061859 17125528 10.1017/bjn20061859

[55] Bajželj B, Laguzzi F, Röös E (2021) The role of fats in the transition to sustainable diets. Lancet Planet Health. 10.1016/S2542-5196(21)00194-7 34508684 10.1016/S2542-5196(21)00194-7

[56] Miller V et al (2022) Global, regional, and National consumption of animal-source foods between 1990 and 2018: findings from the global dietary database. Lancet Planet Health 10.1016/S2542-5196(21)00352-1 10.1016/S2542-5196(21)00352-1 PMC8926870 35278390

[57] Shivarov A (2023) Fish and seafood markets in central and Eastern Europe. Izvestia J union Scientists-Varna. Econ Sci Series 10.56065/IJUSV-ESS/2023.12.1.123

[58] Waijers PM et al (2006) Dietary patterns and survival in older Dutch women. Am J Clin Nutr. 10.1093/ajcn/83.5.1170 16685062 10.1093/ajcn/83.5.1170

[59] Mõttus R, Realo A, Allik J, Deary IJ, Esko T, Metspalu A (2012) Personality traits and eating habits in a large sample of Estonians. Health Psychol. 10.1037/a0027041 22268715 10.1037/a0027041

[60] Bárdos H et al (2022) Diet quality as assessed by healthy eating Index-2015 among Hungarian Roma living in settlements of Northeast Hungary. Sci Rep. 10.1038/s41598-022-23670-3 36357460 10.1038/s41598-022-23670-3 PMC9649748

[61] Eustachio Colombo P et al (2021) Pathways to 5-a-day: modeling the health impacts and environmental footprints of meeting the target for fruit and vegetable intake in the united Kingdom. Am J Clin Nutr. 10.1093/ajcn/nqab076 33871601 10.1093/ajcn/nqab076 PMC8326030

[62] Vega-Cabello V et al (2025) Adherence to the healthy and sustainable dietary recommendations for the Spanish population and all-cause mortality. Rev Esp Cardiol. 10.1016/j.rec.2024.11.008 39645195 10.1016/j.rec.2024.11.008

[63] Watson F, Modi M (2018) Fresh start: A framework for healthy and sustainable diets in the UK-Situational analysis. UK Health Forum 2018. https://ukhealthforum.org.uk/wp-content/uploads/2019/01/UKHF_situational_analysis_FINAL.pdf

[64] Linzmajer M, Eggenschwiler M, Bally L (2022) Der Schweizer Ernährungsatlas – Eine Schätzmethodik des Ernährungsverhaltens der Schweizer Bevölkerung basierend auf Einkaufsdaten. St.Gallen: Forschungszentrum für Handelsmanagement, Universität St.Gallen. https://www.alexandria.unisg.ch/server/api/core/bitstreams/b1adb76a-5d60-4755-bf70-ad214f19d4df/content

[65] Chatelan A, Gaillard P, Kruseman M, Keller A (2019) Total, Added, and free sugar consumption and adherence to guidelines in switzerland: results from the first National nutrition survey menuch. Nutrients. 10.3390/nu11051117 31109151 10.3390/nu11051117 PMC6566881

[66] Bottari F, Mark-Herbert C (2022) Development of uniform food information -the case of front of package nutrition labels in the EU. Arch Public Health. 10.1186/s13690-022-00915-1 35869519 10.1186/s13690-022-00915-1 PMC9306229

[67] Leibinger A et al (2025) The impact of tiered soft drink taxes in Europe on mean sales-weighted sugar content of soft drinks: a quasi-experimental study. BMC Public Health. 10.1186/s12889-025-23331-w 40474135 10.1186/s12889-025-23331-w PMC12142870

[68] Royo-Bordonada MÁ, Capellán LM, Junquera-Abaitua C, López JV, Gómez SF (2023) Spain facing the challenge of regulating unhealthy food advertising. Lancet. 10.1016/S0140-6736(23)00724-9 37149297 10.1016/S0140-6736(23)00724-9

[69] Rust NA et al (2020) How to transition to reduced-meat diets that benefit people and the planet. Sci Total Environ. 10.1016/j.scitotenv.2020.137208 32088475 10.1016/j.scitotenv.2020.137208 PMC7184671

[70] Gu X, Bui LP, Wang F, Wang DD, Springmann M, Willett WC (2024) Global adherence to a healthy and sustainable diet and potential reduction in premature death. Proc Natl Acad Sci USA. 10.1073/pnas.2319008121 39621925 10.1073/pnas.2319008121 PMC11648617

[71] OECD, FAO (2021) OECD-FAO agricultural outlook 2021–2030. OECD Publishing, Paris. 10.1787/19428846-en

[72] de la Aznar MDC et al (2025) Health and environmental dietary impact: planetary health diet vs. Mediterranean diet. A nationwide cohort in Spain. Sci Total Environ. 10.1016/j.scitotenv.2025.178924 10.1016/j.scitotenv.2025.178924 39987831

[73] Colizzi C et al (2023) Adherence to the EAT-Lancet healthy reference diet in relation to risk of cardiovascular events and environmental impact: results from the EPIC-NL cohort. J Am Heart Assoc. 10.1161/JAHA.122.026318 37066787 10.1161/JAHA.122.026318 PMC10227249

[74] Wang Y et al (2025) Planetary health diet and risk of mortality and chronic diseases: results from US NHANES, UK Biobank, and a meta-analysis. Sci Adv. 10.1126/sciadv.adq5147 40911663 10.1126/sciadv.adq5147 PMC12412635

[75] Llanaj E et al (2021) Deteriorated dietary patterns with regards to health and environmental sustainability among Hungarian Roma are not differentiated from those of the general population. Nutrients 10.3390/nu13030721 10.3390/nu13030721 PMC7996132 33668386

[76] Cué Rio M et al (2022) The elephant in the room is really a cow: using consumption corridors to define sustainable meat consumption in the European union. Sustain Sci. 10.1007/s11625-022-01235-7

[77] Castaldi S, Dembska K, Antonelli M, Petersson T, Piccolo MG, Valentini R (2022) The positive climate impact of the mediterranean diet and current divergence of mediterranean countries towards less climate sustainable food consumption patterns. Sci Rep. 10.1038/s41598-022-12916-9 35614126 10.1038/s41598-022-12916-9 PMC9132980

[78] Sandri E, Cantín Larumbe E, Part-Ferrer R, Ferrer-Torregrosa J, Fernández-Ehrling N (2023) Diet and lifestyle in the Spanish population and their relationship with sociodemographic variables: a descriptive study. Foods 10.3390/foods12183409 10.3390/foods12183409 PMC10527864 37761118

[79] Moncho J, Ortega Sarabia LE, Trescastro-López EM, Martínez-García A (2025) Differences in dietary patterns between native and immigrant populations in Spain. PLoS One. 10.1371/journal.pone.0328458 40690418 10.1371/journal.pone.0328458 PMC12279135

[80] Bertoni Maluf VA et al (2022) Description of Ultra-Processed food intake in a Swiss Population-Based sample of adults aged 18 to 75 years. Nutrients 10.3390/nu14214486 10.3390/nu14214486 PMC9659134 36364749

[81] Steinbach L et al (2021) No-meat eaters are less likely to be overweight or obese, but take dietary supplements more often: results from the Swiss National nutrition survey menuch. Public Health Nutr. 10.1017/S1368980020003079 32893771 10.1017/S1368980020003079 PMC10195288

[82] Xu C, Cao Z, Yang H, Hou Y, Wang X, Wang Y (2022) Association between the EAT-Lancet diet pattern and risk of type 2 diabetes: a prospective cohort study. Front Nutr 10.3389/fnut.2021.784018 10.3389/fnut.2021.784018 PMC8795697 35096931

[83] de Moraes MM et al (2021) An Ultra-Processed food dietary pattern is associated with lower diet quality in Portuguese adults and the elderly: the UPPER project. Nutrients 10.3390/nu13114119 10.3390/nu13114119 PMC8619325 34836373

[84] Lehto E, Kaartinen NE, Sääksjärvi K, Männistö S, Jallinoja P (2022) Vegetarians and different types of meat eaters among the Finnish adult population from 2007 to 2017. Br J Nutr. 10.1017/S0007114521001719 34184978 10.1017/S0007114521001719 PMC8924490

[85] Kähäri A (2022) Gender differences in fresh vegetable intake from 1979 to 2017 in Finland. Br Food J. 10.1108/BFJ-09-2021-1004

[86] Goryńska-Goldmann E, Murawska A, Balcerowska-Czerniak G (2023) Consumer profiles of sustainable fruit and vegetable consumption in the European union. Sustainability 10.3390/su152115512

[87] Stewart C, Piernas C, Cook B, Jebb SA (2021) Trends in UK meat consumption: analysis of data from years 1–11 (2008-09 to 2018-19) of the National diet and nutrition survey rolling programme. Lancet Planet Health. 10.1016/s2542-5196(21)00228-x 34627474 10.1016/S2542-5196(21)00228-X PMC8515514

[88] Toujgani H et al (2024) Dietary pattern trajectories in French adults of the NutriNet-Santé cohort over time (2014–2022): role of socio-economic factors. Br J Nutr. 10.1017/S0007114524002514 39417338 10.1017/S0007114524002514 PMC11617108

[89] Carvalho C, Correia D, Lopes C, Torres D (2025) Adherence to the EAT-Lancet planetary health diet in Portugal and its associations with socioeconomic and lifestyle factors. Eur J Nutr. 10.1007/s00394-025-03661-6 40205117 10.1007/s00394-025-03661-6 PMC11982082

[90] van Bussel LM, van Rossum CT, Temme EH, Boon PE, Ocké MC (2020) Educational differences in healthy, environmentally sustainable and safe food consumption among adults in the Netherlands. Public Health Nutr. 10.1017/S1368980019005214 32383426 10.1017/S1368980019005214 PMC10200583

[91] Cai YW et al (2025) Adherence to EAT-Lancet diet, biological aging, and life expectancy in the UK biobank: a cohort study. Am J Clin Nutr. 10.1016/j.ajcnut.2025.04.030 40339905 10.1016/j.ajcnut.2025.04.030

[92] Hu FL et al (2025) EAT-Lancet diet pattern, genetic risk, and risk of colorectal cancer: a prospective study from the UK biobank. Am J Clin Nutr. 10.1016/j.ajcnut.2025.02.025 39993568 10.1016/j.ajcnut.2025.02.025

[93] Govzman S, Looby S, Wang X, Butler F, Gibney ER, Timon CM (2021) A systematic review of the determinants of seafood consumption. Br J Nutr. 10.1017/S0007114520003773 32967738 10.1017/S0007114520003773

[94] Mieziene B, Emeljanovas A, Fatkulina N, Stukas R (2020) Dietary pattern and its correlates among Lithuanian young adults: mediterranean diet approach. Nutrients. 10.3390/nu12072025 32650389 10.3390/nu12072025 PMC7400829

[95] Prättälä R, Paalanen L, Grinberga D, Helasoja V, Kasmel A, Petkeviciene J (2007) Gender differences in the consumption of meat, fruit and vegetables are similar in Finland and the Baltic countries. Eur J Public Health. 10.1093/eurpub/ckl265 17194710 10.1093/eurpub/ckl265

[96] Dinnissen CS, Ocké MC, Buurma-Rethans EJM, van Rossum CTM (2021) Dietary changes among adults in the Netherlands in the period 2007–2010 and 2012–2016. Results from two Cross-Sectional National food consumption surveys. Nutrients 10.3390/nu13051520. 10.3390/nu13051520 PMC8146163 33946365

[97] Abuladze L, Kunder N, Lang K, Vaask S (2017) Associations between self-rated health and health behaviour among older adults in estonia: a cross-sectional analysis. BMJ Open. 10.1136/bmjopen-2016-013257 28601816 10.1136/bmjopen-2016-013257 PMC5734211

[98] Mata J, Kadel P, Frank R, Schüz B (2023) Education- and income-related differences in processed meat consumption across europe: the role of food-related attitudes. Appetite 10.1016/j.appet.2022.106417. 10.1016/j.appet.2022.106417 36521648

[99] Thompson FE et al (2015) The national cancer institute’s dietary assessment primer: a resource for diet research. J Acad Nutr Diet 10.1016/j.jand.2015.08.016. 10.1016/j.jand.2015.08.016 PMC4663113 26422452

[100] Hoffmann K et al (2002) Estimating the distribution of usual dietary intake by short-term measurements. Eur J Clin Nutr. 10.1038/sj.ejcn.1601429 12082518 10.1038/sj.ejcn.1601429

[101] Dodd KW et al (2006) Statistical methods for estimating usual intake of nutrients and foods: a review of the theory. J Am Diet Assoc. 10.1016/j.jada.2006.07.011 17000197 10.1016/j.jada.2006.07.011

[102] Joslowski G et al (2017) Development of a harmonized food grouping system for between-country comparisons in the TEDDY study. J Food Compost Anal. 10.1016/j.jfca.2017.07.037 29151672 10.1016/j.jfca.2017.07.037 PMC5690566

[103] Baudry J et al (2023) Associations between measures of socio-economic position and sustainable dietary patterns in the NutriNet-Santé study. Public Health Nutr. 10.1017/S1368980022002208 36213945 10.1017/S1368980022002208 PMC10346073

[104] Azaïs-Braesco V, Sluik D, Maillot M, Kok F, Moreno LA (2017) A review of total & added sugar intakes and dietary sources in Europe. Nutr J. 10.1186/s12937-016-0225-2 28109280 10.1186/s12937-016-0225-2 PMC5251321

[105] Rockström J et al (2025) The EAT-Lancet commission on healthy, sustainable, and just food systems. Lancet. 10.1016/S0140-6736(25)01201-2 41046857 10.1016/S0140-6736(25)01201-2

[106] European Commission (2020) Farm to Fork strategy. https://food.ec.europa.eu/horizontal-topics/farm-fork-strategy_en. Accessed 24 September 2025

[107] Dimbleby H (2021) National Food Strategy: The Plan. https://www.nationalfoodstrategy.org/wp-content/uploads/2021/10/25585_1669_NFS_The_Plan_July21_S12_New-1.pdf. Accessed 24 September 2025

[108] UK Research and Innovation (2025) Roadmap for Resilience: A UK Food Plan for 2050. https://www.agrifood4netzero.net/wp-content/uploads/2025/10/AFN-ROADMAP.pdf. Accessed 15 December 2025

[109] Fakhri M (2025) Corporate power and human rights in food systems - Report of the Special Rapporteur on the right to food. A/80/213. https://www.ohchr.org/en/documents/thematic-reports/a80213-corporate-power-and-human-rights-food-systems-report-special. Accessed 24 September 2025

[110] Changing Markets (2024) The new merchants of doubt: how big meat and dairy avoid climate action. https://changingmarkets.org/wp-content/uploads/2024/07/Report-Summary-Eng.pdf. Accessed 24 September 2025

