# Supplementary material for: Are European diets healthy and sustainable? Evidence from nine countries using the planetary health diet framework
Source: Eur J Nutr. 2026 Mar 7;65(3):82. doi: 10.1007/s00394-026-03929-5 (PMC12967455; doi:10.1007/s00394-026-03929-5)
Supplement: Supplementary file 1 — Supplementary Material 1 [file 394_2026_3929_MOESM1_ESM.pdf]

Are European Diets Healthy and Sustainable? Evidence from Nine Countries Using the Planetary Health Diet Framework

Authors: Agustin R. Miranda, Joseph M.M. Meunier, Sofia Romagosa Vilarnau, Anant Jani, Eric O. Verger

Supplementary Materials

Supplementary Figures 3

Figure S1. Sampling flow chart and sample size. 3

Figure S2. Between-country heterogeneity in consumption of encouraged food groups (g/day). 4

Figure S3. Between-country heterogeneity in consumption of foods to moderate and to limit (g/day). 5

Figure S4. Percentage of compliance with the PHD targets across nine European countries. 6

Figure S5. Mean total scores in PHD indices across countries. 7

Figure S6. Between-country heterogeneity in PHD indices. 8

Figure S7. Correlation and agreement between PHD indices. 9

Figure S8. Component-level heatmap showing scoring level of each food group per country. 10

Figure S19. Cross-country comparison of food-group influence rankings on the WISH index based on dominance analysis. 11

Figure S10. Cross-country comparison of food-group influence rankings on the ELI index based on dominance analysis. 12

Figure S11. Cross-country comparison of food-group influence rankings on the ELD-I index based on dominance analysis. 13

Figure S12. PHD indices according to sex in nine European countries. 14

Figure S13. PHD indices according to age in nine European countries. 15

Figure S14. PHD indices according to education level in nine European countries. 16

Figure S15. Sensitivity analysis assessing the influence of survey design on global mean. 17

Supplementary Tables 18

Table S1. Recipes used to disaggregate dishes into food groups for intake estimation. 18

Table S2. Number of participants excluded due to implausible energy intakes, by country, sex, and age group 27

Table S3. EAT-Lancet reference diet, with possible ranges, for an intake of 2,500 kcal/day 28

Table S4. World Index for Sustainability and Health 30

Table S5. EAT-Lancet Index 31

Table S6. EAT-Lancet diet index 32

Table S7. Survey respondent distribution by country, sex, and age group: Counts, within-group proportions, and country weights 33

Table S8. Weighted proportions 36

Table S9. Sex-specific mean consumption of PHD food groups across countries (g/day). ... 37

Table S10. Age-specific mean consumption of PHD food groups across countries (g/day). ... 38

Table S11. Dominance analysis of food group consumption (g/day) on the WISH index. ... 40

Table S12. Dominance analysis of food group consumption (g/day) on the ELI index. ... 41

Table S13. Dominance analysis of food group consumption (g/day) on the EDL-I index. ... 42

Table S14. Multivariate regression analyses of sociodemographic factors associated with WISH, ELI, and ELD-I in the United Kingdom. ... 43

Table S15. Multivariate regression analyses of sociodemographic factors associated with WISH, ELI, and ELD-I in France. ... 44

Table S16. Multivariate regression analyses of sociodemographic factors associated with WISH, ELI, and ELD-I in Spain. ... 45

Table S17. Multivariate regression analyses of sociodemographic factors associated with WISH, ELI, and ELD-I in the Netherlands. ... 46

Table S18. Multivariate regression analyses of sociodemographic factors associated with WISH, ELI, and ELD-I in Portugal. ... 47

Table S19. Multivariate regression analyses of sociodemographic factors associated with WISH, ELI, and ELD-I in Hungary. ... 48

Table S20. Multivariate regression analyses of sociodemographic factors associated with WISH, ELI, and ELD-I in Switzerland. ... 49

Table S21. Multivariate regression analyses of sociodemographic factors associated with WISH, ELI, and ELD-I in Finland. ... 50

Table S22. Multivariate regression analyses of sociodemographic factors associated with WISH, ELI, and ELD-I in Estonia. ... 51

Extended discussion: Heterogeneity in European food consumption ... 52

Table S23. Example multi-level and -sectoral actions that can promote healthier and more sustainable food choice architecture ... 54

Supplementary Figures

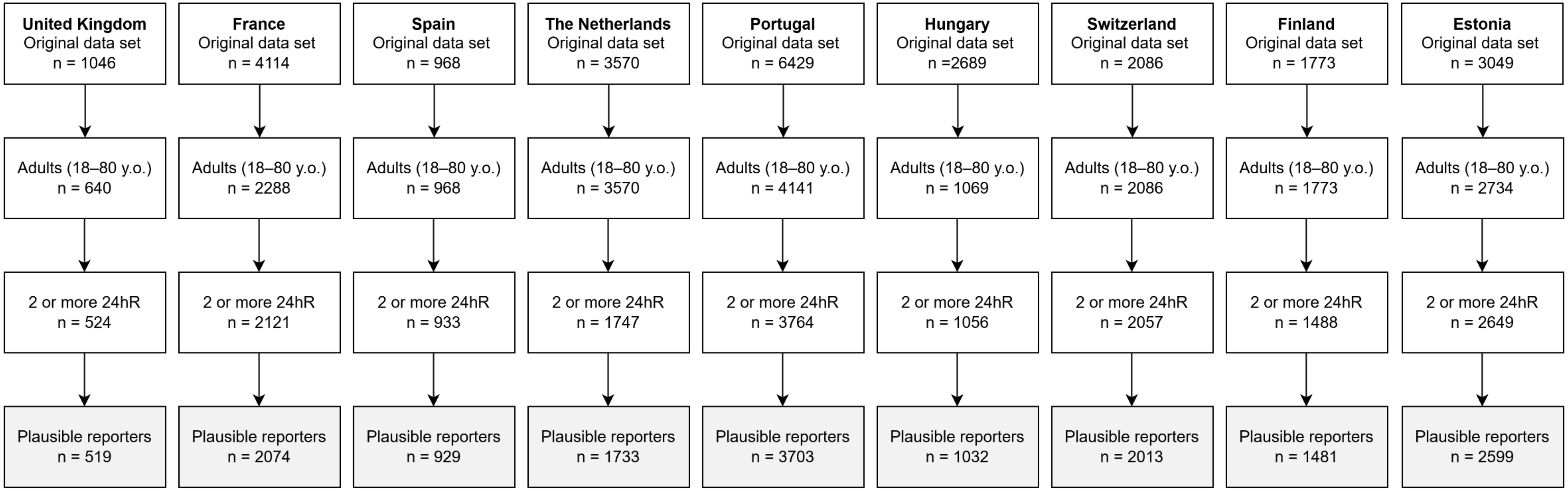

Figure S1. Sampling flow chart and sample size. Participants reporting extreme energy intakes (<800 or >4200 kcal/day for men; <600 or >3500 kcal/day for women) were considered as mis-reporters. 24hR = non-consecutive 24-hour dietary recalls. This flowchart illustrates the stepwise application of inclusion criteria; it does not show sample characteristics.

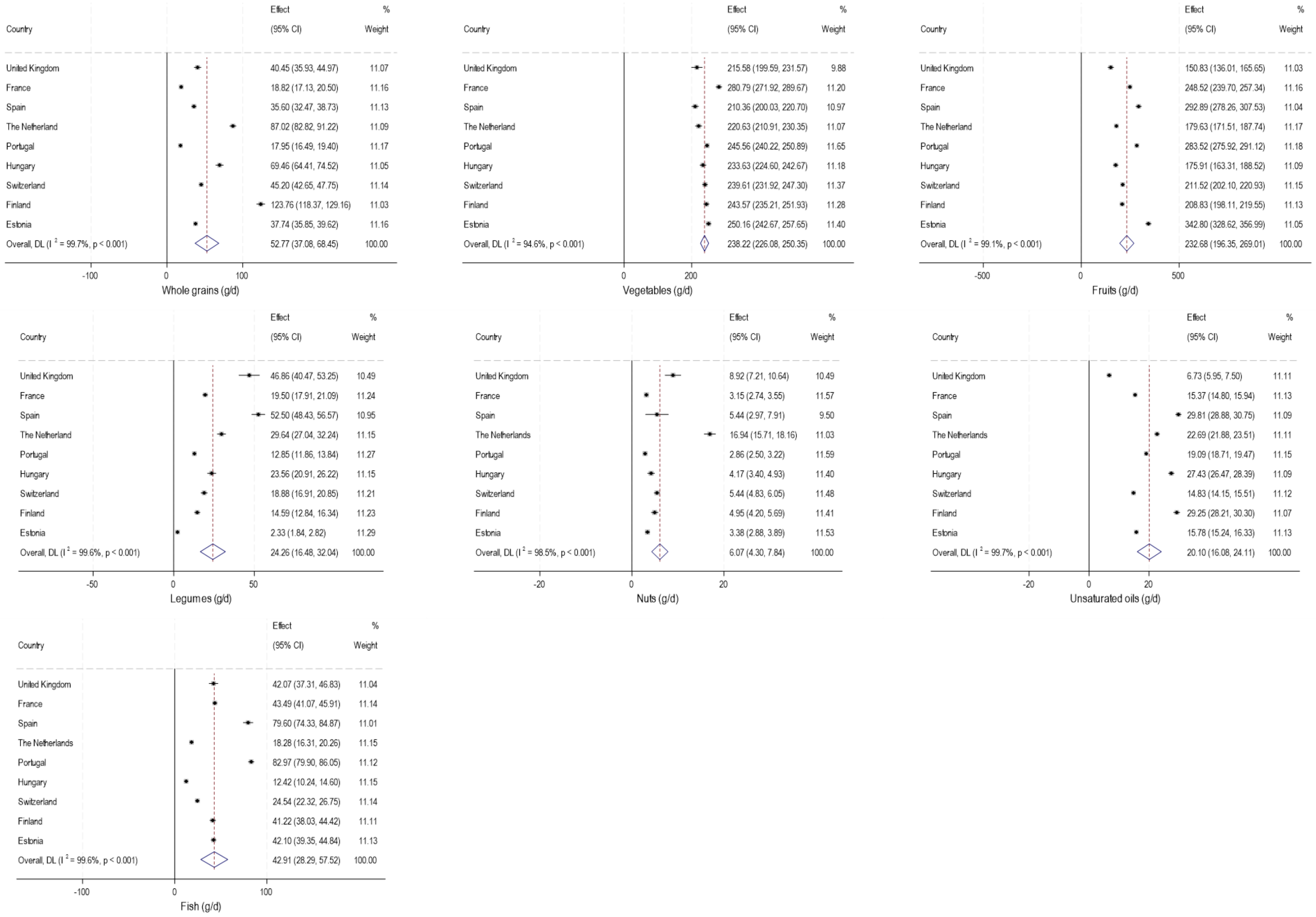

Figure S2. Between-country heterogeneity in consumption of encouraged food groups (g/day). I^2 indicates the percentage of total variability attributable to between-country heterogeneity. Effect estimates are presented as means with 95% confidence intervals. Diamonds indicate pooled estimates.

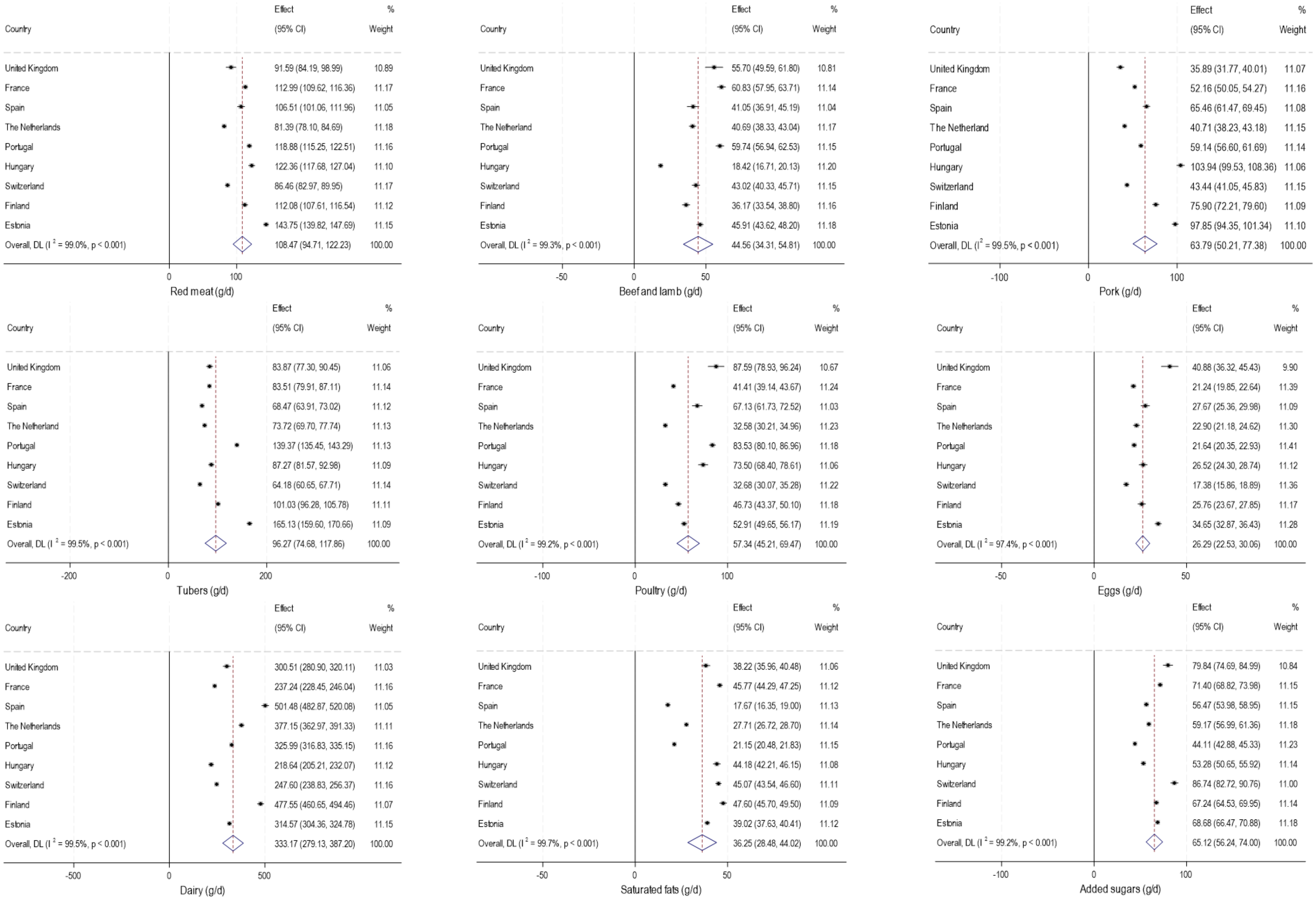

Figure S3. Between-country heterogeneity in consumption of foods to moderate and to limit (g/day). I^2 indicates the percentage of total variability attributable to between-country heterogeneity. Effect estimates are presented as means with 95% confidence intervals. Diamonds indicate pooled estimates.

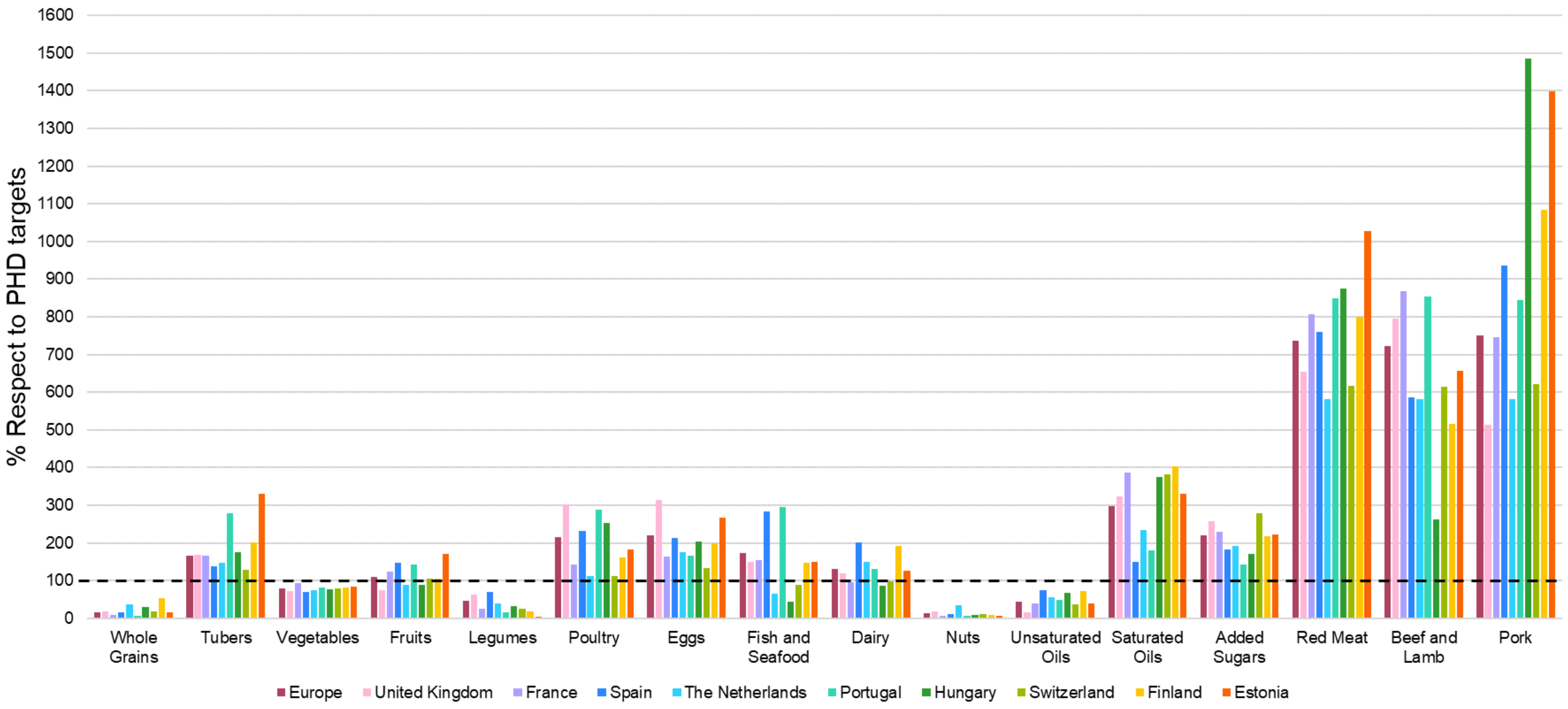

Figure S4. Percentage of compliance with the PHD targets across nine European countries. Bars show the average national intake per food group as a percentage of the PHD target (dashed line = 100 %). Values above the dashed line indicate intake exceeding the target, and values below it indicates under-consumption.

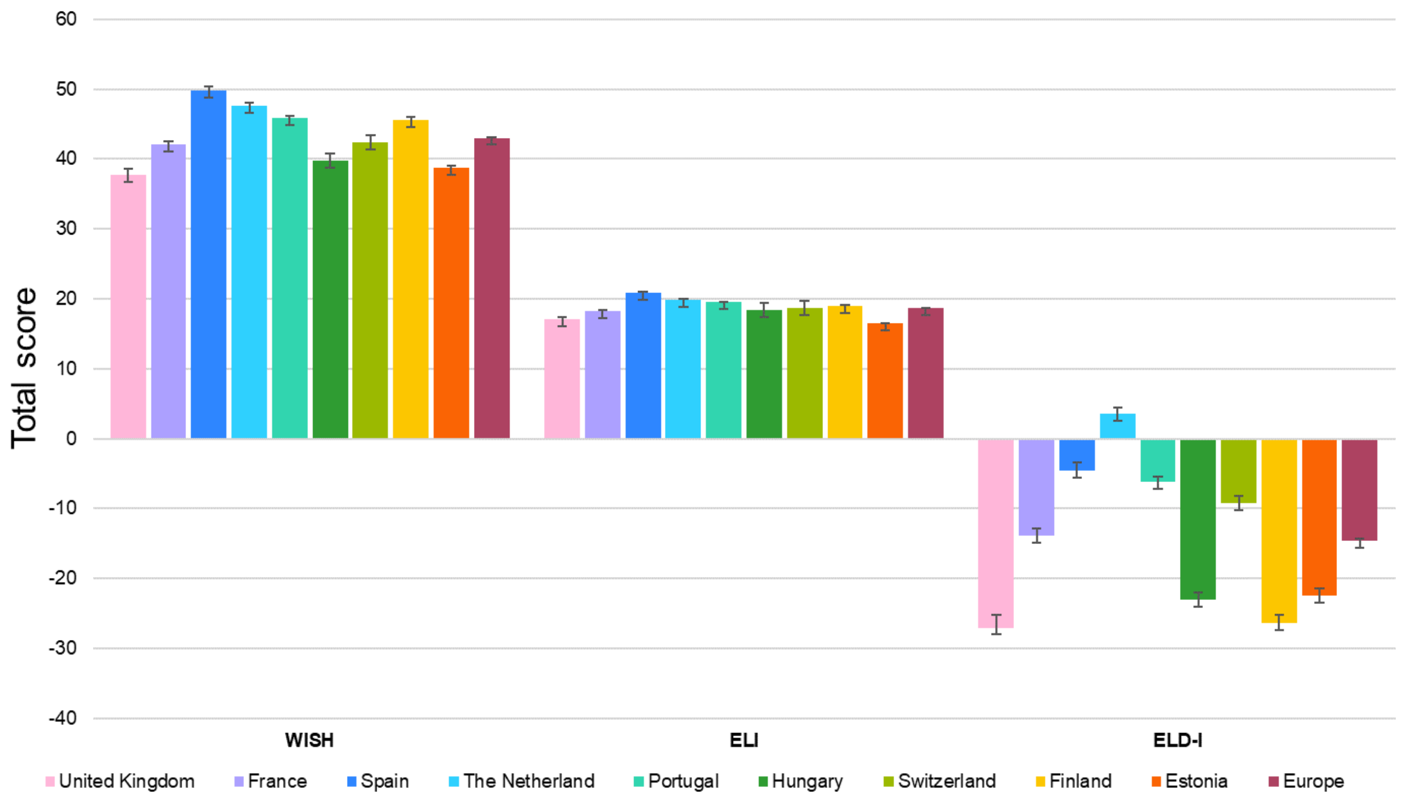

Figure S5. Mean total scores in PHD indices across countries.

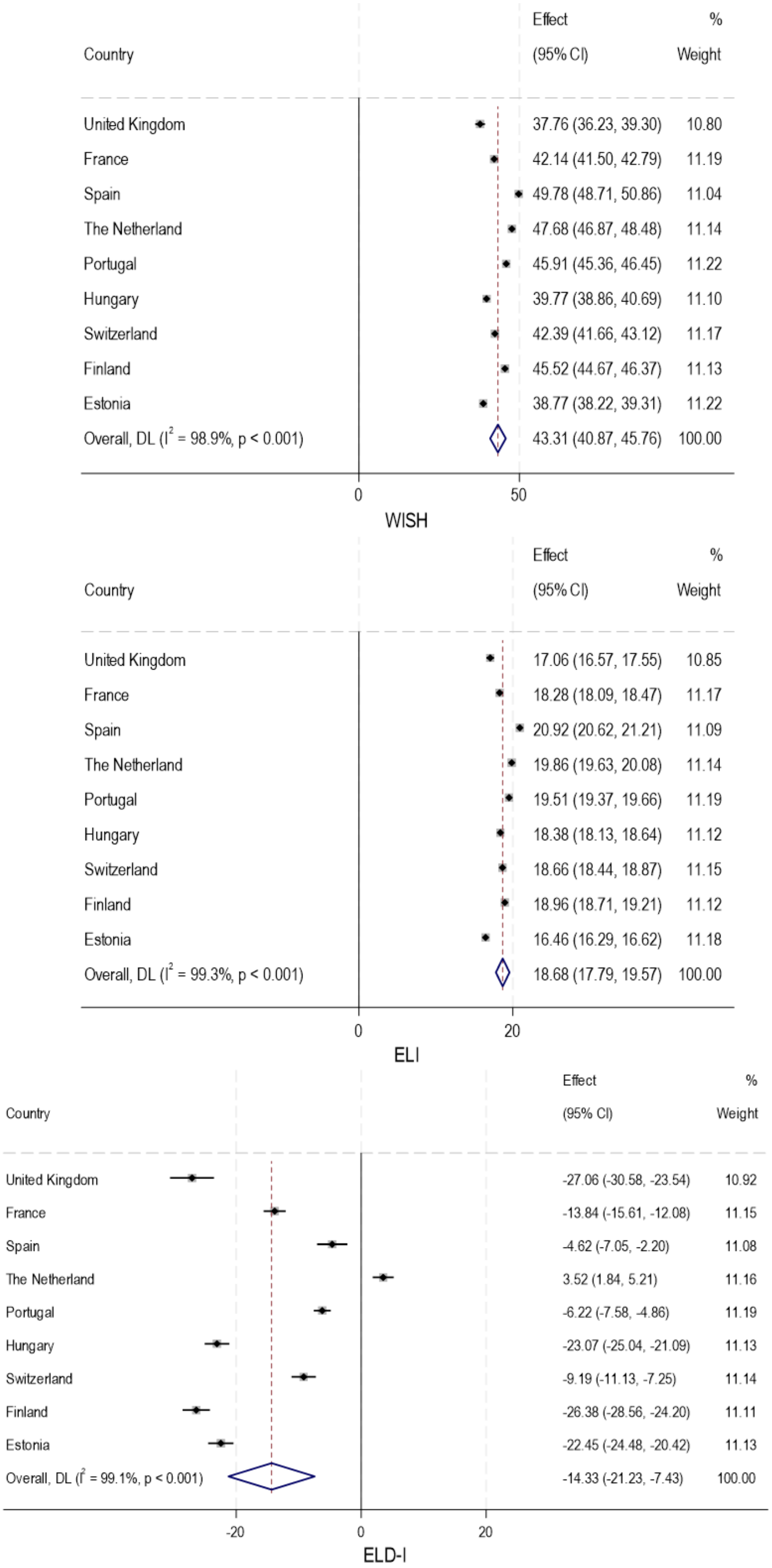

Figure S6. Between-country heterogeneity in PHD indices. I^2 indicates the percentage of total variability attributable to between-country heterogeneity. Effect estimates are presented as means with 95% confidence intervals. Diamonds indicate pooled estimates.

	Pearson correlation			Quintile agreement (%)		
	WISH/ELI	WISH/ELDI	ELDI/ELI	WISH/ELI	WISH/ELDI	ELDI/ELI
Europe	0.80	0.56	0.66	0.48	0.36	0.40
Estonia	0.73	0.50	0.61	0.41	0.32	0.37
Finland	0.81	0.55	0.65	0.48	0.34	0.39
France	0.75	0.46	0.59	0.43	0.32	0.37
Hungary	0.73	0.46	0.56	0.44	0.32	0.34
Portugal	0.76	0.56	0.63	0.44	0.34	0.37
Spain	0.75	0.50	0.63	0.42	0.35	0.37
Switzerland	0.82	0.58	0.64	0.47	0.37	0.38
Netherlands	0.80	0.58	0.69	0.46	0.34	0.40
United Kingdom	0.85	0.62	0.70	0.50	0.40	0.39

Figure S7. Correlation and agreement between PHD indices.

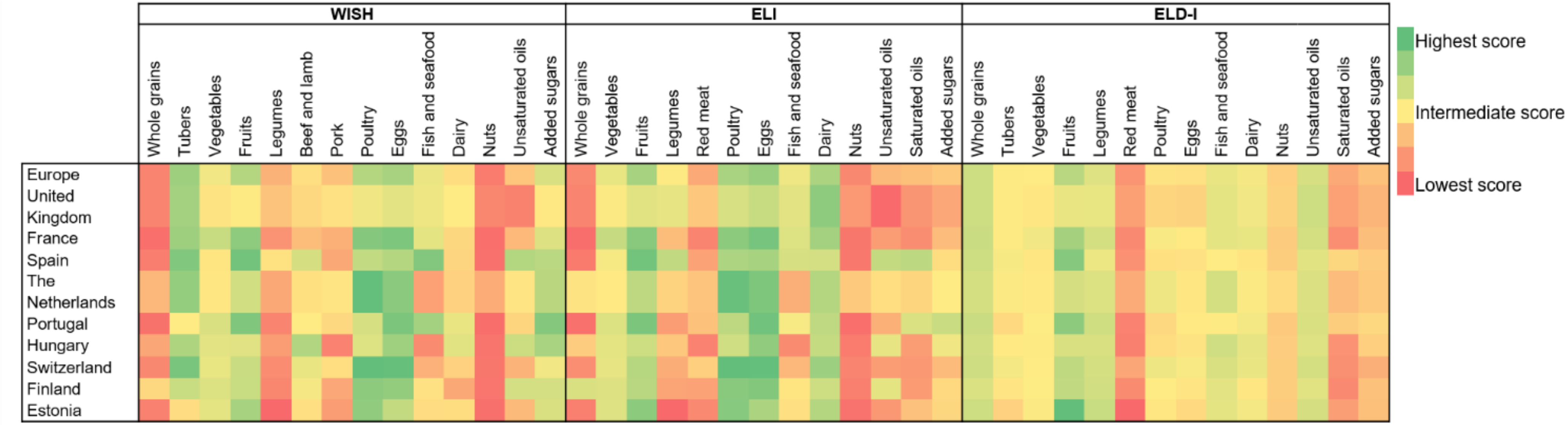

Figure S8. Component-level heatmap showing scoring level of each food group per country. In the heatmap, colours indicate the relative score within each index.

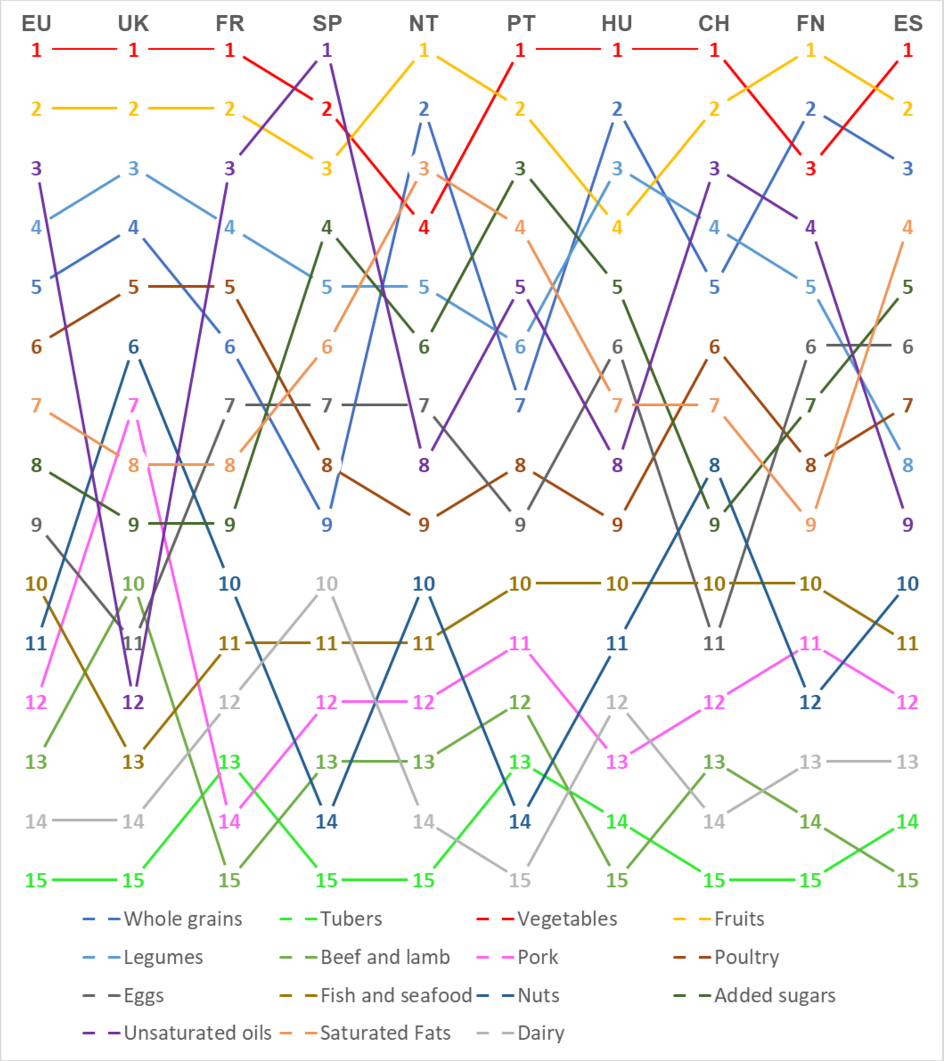

Figure S19. Cross-country comparison of food-group influence rankings on the WISH index based on dominance analysis.

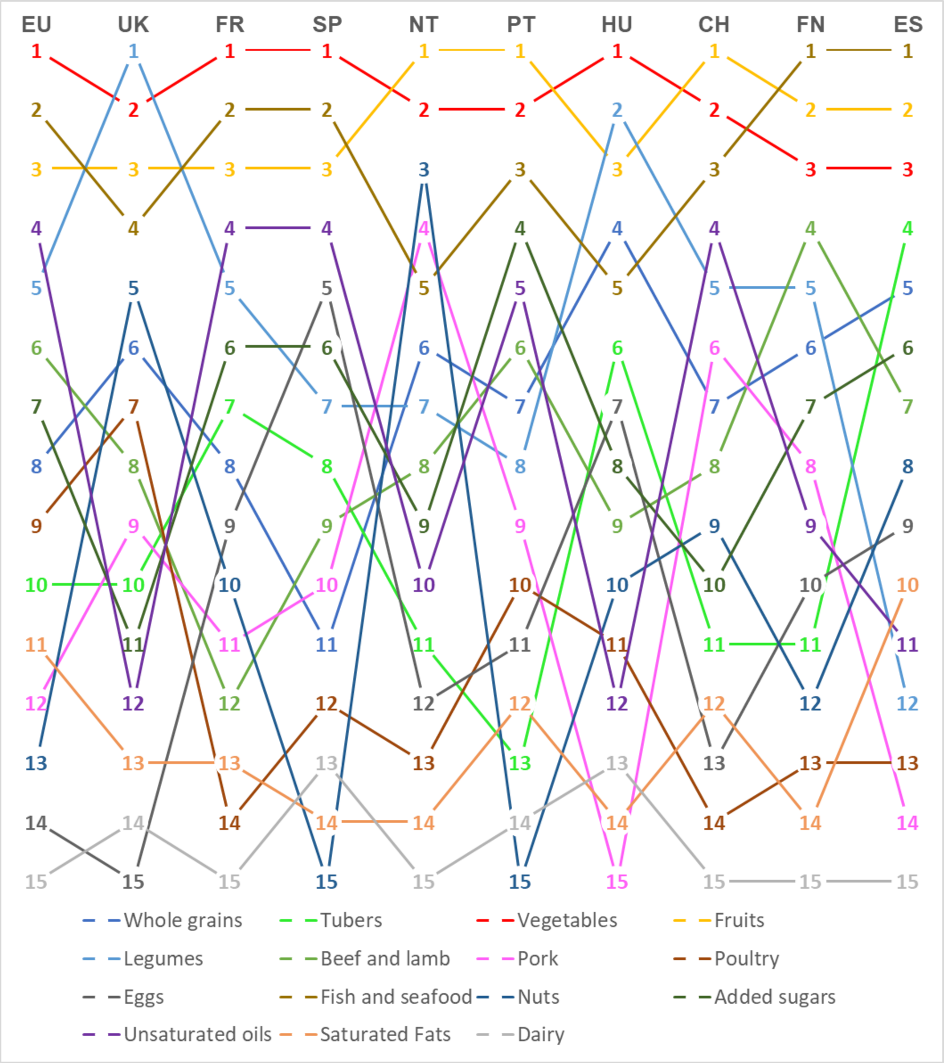

Figure S10. Cross-country comparison of food-group influence rankings on the ELI index based on dominance analysis.

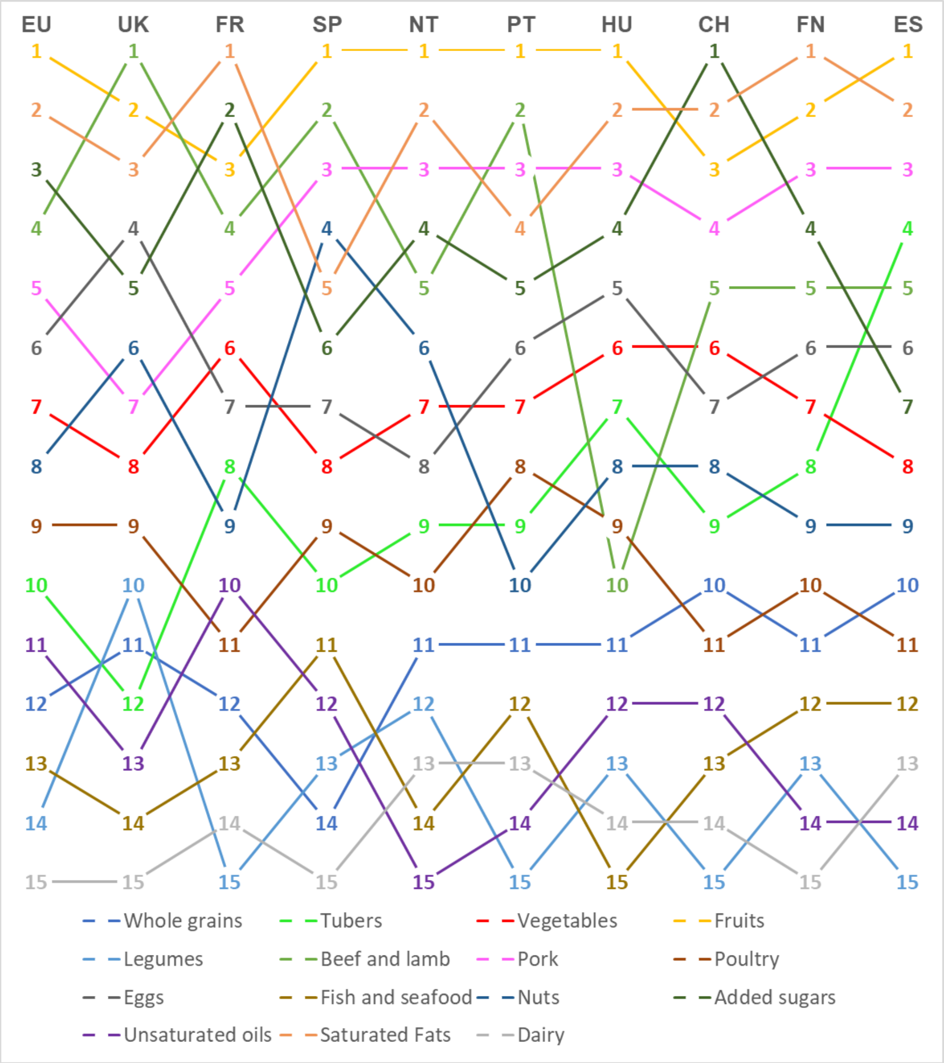

Figure S11. Cross-country comparison of food-group influence rankings on the ELD-I index based on dominance analysis.

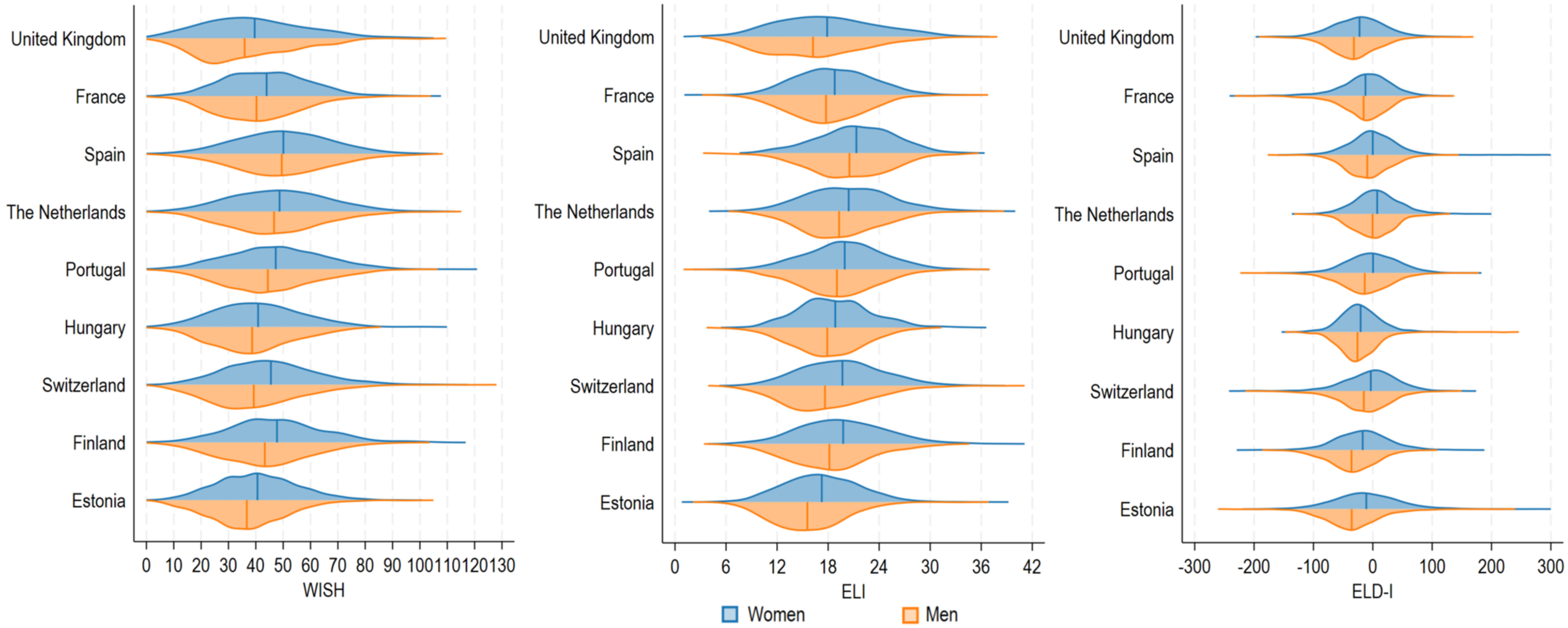

Figure S12. PHD indices according to sex in nine European countries. Lines inside the violins denotes means.

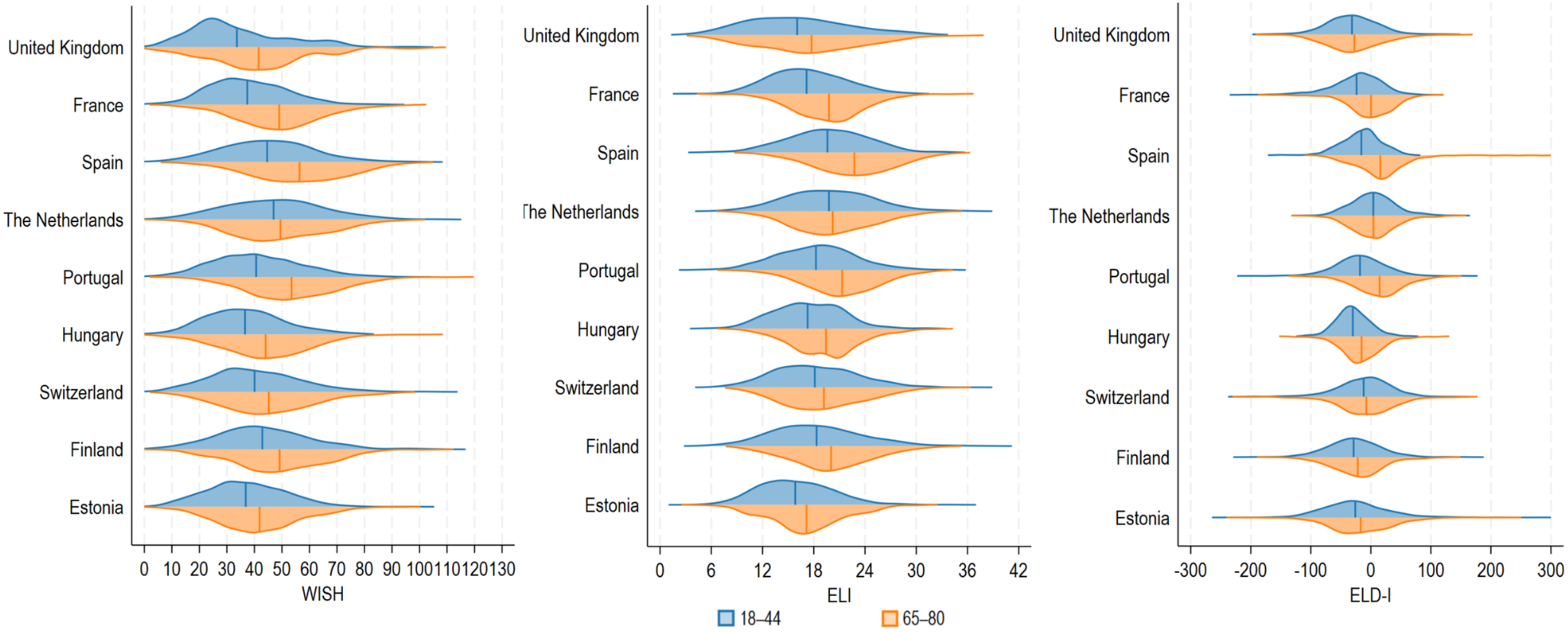

Figure S13. PHD indices according to age in nine European countries. Lines inside the violins denotes means.

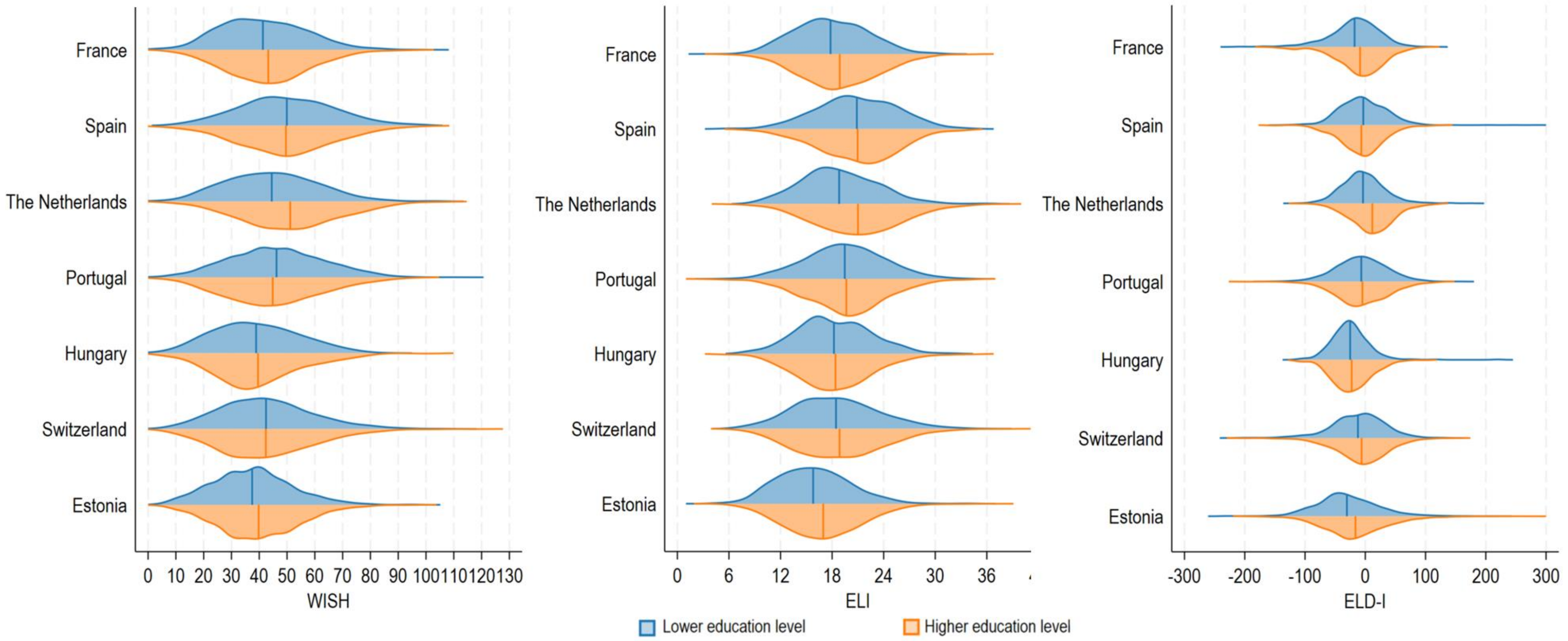

Figure S14. PHD indices according to education level in nine European countries. Lines inside the violins denotes means.

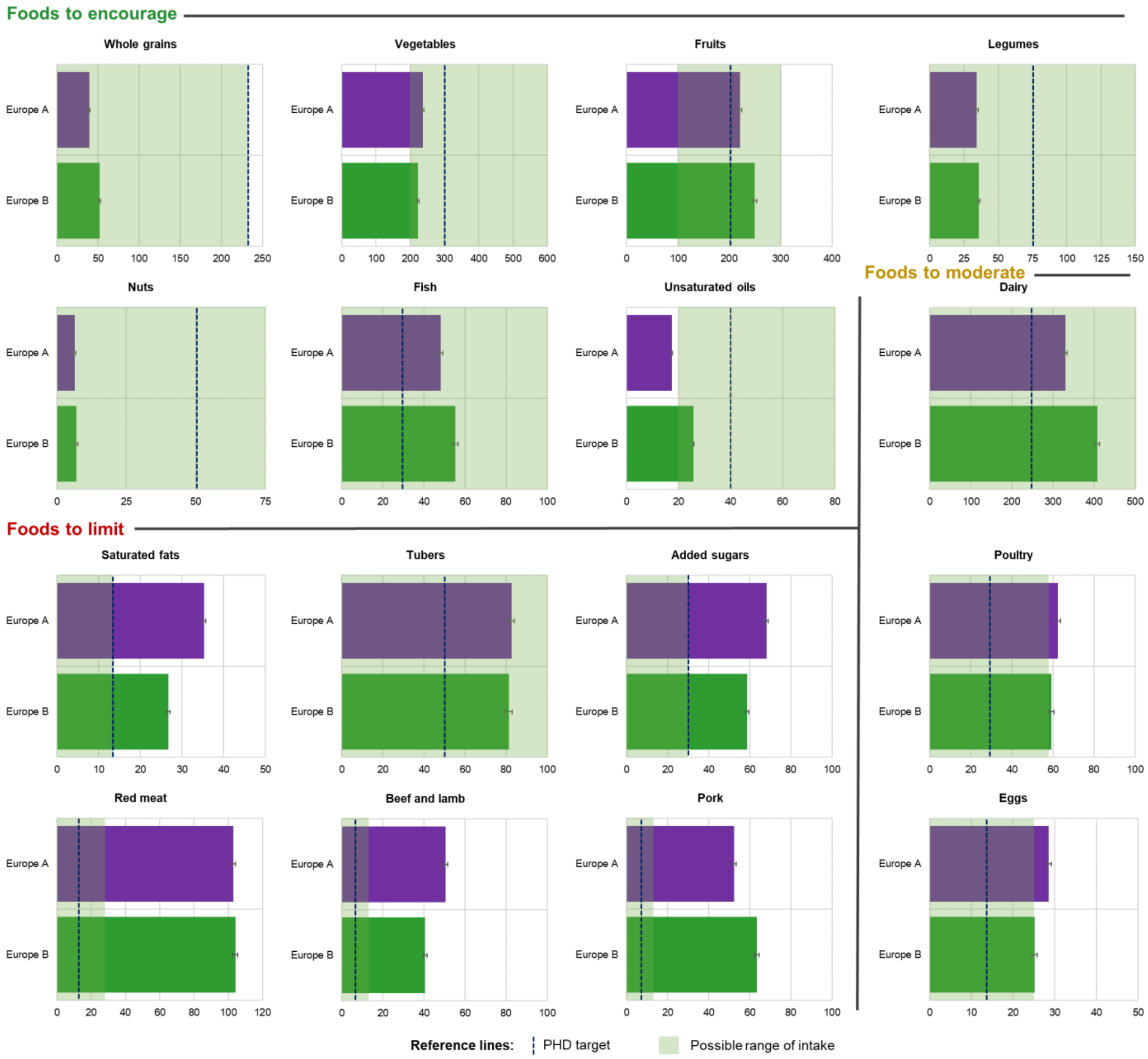

Figure S15. Sensitivity analysis assessing the influence of survey design on global mean. A = European average intakes including all countries; B = same estimates after excluding the UK and France (countries with a higher number of recall days). Intakes are standardised to 2,500 kcal.

Supplementary Tables

Table S1. Recipes used to disaggregate dishes into food groups for intake estimation. Values in %.																			
**ID_Recipe**	**Category**	**Recipe**	**Whole grains**	**Tubers**	**Vegetables**	**Fruits**	**Legumes**	**Red meat (no pork)**	**Pork**	**Poultry**	**Eggs**	**Fish and seafood**	**Milk**	**Cheese**	**Cream**	**Butter**	**Nuts**	**Oils**	**Sugars**
1.	Dishes with dough / Pasta / Pâte	Pasta / Meat	0.00	0.00	0.00	0.00	0.00	10.00	0.00	0.00	0.00	0.00	0.00	0.00	3.00	2.00	0.00	5.00	0.00
2.	Dishes with dough / Pasta / Pâte	Pasta / Pork	0.00	0.00	0.00	0.00	0.00	0.00	10.00	0.00	0.00	0.00	0.00	0.00	3.00	2.00	0.00	5.00	0.00
3.	Dishes with dough / Pasta / Pâte	Pasta / Poultry	0.00	0.00	0.00	0.00	0.00	0.00	0.00	10.00	0.00	0.00	0.00	0.00	3.00	2.00	0.00	5.00	0.00
4.	Dishes with dough / Pasta / Pâte	Pasta / Fish	0.00	0.00	0.00	0.00	0.00	0.00	0.00	0.00	0.00	10.00	0.00	0.00	3.00	2.00	0.00	5.00	0.00
5.	Dishes with dough / Pasta / Pâte	Pasta / Vegetables	0.00	0.00	10.00	0.00	5.00	0.00	0.00	0.00	0.00	0.00	0.00	0.00	3.00	2.00	0.00	5.00	0.00

6.	Soups and Stews	Soup / Meat	0.00	10.00	40.00	0.00	5.00	10.00	0.00	0.00	0.00	0.00	0.00	0.00	0.00	0.00	0.00	3.00	0.00
7.	Soups and Stews	Soup / Pork	0.00	10.00	40.00	0.00	5.00	0.00	10.00	0.00	0.00	0.00	0.00	0.00	0.00	0.00	0.00	3.00	0.00
8.	Soups and Stews	Soup / Poultry	0.00	10.00	40.00	0.00	5.00	0.00	0.00	10.00	0.00	0.00	0.00	0.00	0.00	0.00	0.00	3.00	0.00
9.	Soups and Stews	Soup / Vegetables	0.00	10.00	55.00	0.00	5.00	0.00	0.00	0.00	0.00	0.00	0.00	0.00	0.00	0.00	0.00	3.00	0.00
10.	Soups and Stews	Soup / Fish	0.00	10.00	40.00	0.00	5.00	0.00	0.00	0.00	0.00	15.00	0.00	0.00	0.00	0.00	0.00	3.00	0.00
11.	Burgers	Burger / Meat	0.00	0.00	10.00	0.00	0.00	40.00	0.00	0.00	0.00	0.00	0.00	5.00	0.00	0.00	0.00	0.00	0.00
12.	Burgers	Burger / Pork	0.00	0.00	10.00	0.00	0.00	0.00	40.00	0.00	0.00	0.00	0.00	5.00	0.00	0.00	0.00	0.00	0.00
13.	Burgers	Burger / Pork / Meat	0.00	0.00	10.00	0.00	0.00	0.00	40.00	0.00	0.00	0.00	0.00	5.00	0.00	0.00	0.00	0.00	0.00
14.	Burgers	Burger / Egg / Bacon	0.00	0.00	0.00	0.00	0.00	0.00	10.00	0.00	20.00	0.00	0.00	5.00	0.00	0.00	0.00	0.00	0.00
15.	Burgers	Burger / Poultry	0.00	0.00	10.00	0.00	0.00	0.00	0.00	40.00	0.00	0.00	0.00	5.00	0.00	0.00	0.00	0.00	0.00
16.	Burgers	Burger / Vegetarian	0.00	0.00	10.00	0.00	40.00	0.00	0.00	0.00	0.00	0.00	0.00	5.00	0.00	0.00	0.00	0.00	0.00
17.	Burgers	Burger / Fish	0.00	0.00	10.00	0.00	0.00	0.00	0.00	0.00	0.00	40.00	0.00	5.00	0.00	0.00	0.00	0.00	0.00
18.	Salads	Salad / Meat	0.00	10.00	60.00	0.00	5.00	10.00	0.00	0.00	5.00	0.00	0.00	0.00	0.00	0.00	0.00	5.00	0.00
19.	Salads	Salad / Pork	0.00	10.00	60.00	0.00	5.00	0.00	10.00	0.00	5.00	0.00	0.00	0.00	0.00	0.00	0.00	5.00	0.00
20.	Salads	Salad / Poultry	0.00	10.00	60.00	0.00	5.00	0.00	0.00	10.00	5.00	0.00	0.00	0.00	0.00	0.00	0.00	5.00	0.00
21.	Salads	Salad / Vegetables	0.00	10.00	70.00	0.00	5.00	0.00	0.00	0.00	5.00	0.00	0.00	0.00	0.00	0.00	0.00	5.00	0.00
22.	Salads	Salad / Fish	0.00	10.00	60.00	0.00	5.00	0.00	0.00	0.00	5.00	10.00	0.00	0.00	0.00	0.00	0.00	5.00	0.00
23.	Sandwiches	Sandwich / Meat	0.00	0.00	5.00	0.00	0.00	25.00	0.00	0.00	5.00	0.00	0.00	5.00	0.00	5.00	0.00	0.00	0.00
24.	Sandwiches	Sandwich / Meat / Whole grain bread	50.00	0.00	5.00	0.00	0.00	25.00	0.00	0.00	5.00	0.00	0.00	5.00	0.00	5.00	0.00	0.00	0.00
25.	Sandwiches	Sandwich / Pork	0.00	0.00	5.00	0.00	0.00	0.00	25.00	0.00	5.00	0.00	0.00	5.00	0.00	5.00	0.00	0.00	0.00
26.	Sandwiches	Sandwich / Pork / Whole grain bread	50.00	0.00	5.00	0.00	0.00	0.00	25.00	0.00	5.00	0.00	0.00	5.00	0.00	5.00	0.00	0.00	0.00

27.	Sandwiches	Sandwich / Poultry	0.00	0.00	5.00	0.00	0.00	0.00	0.00	25.00	5.00	0.00	0.00	5.00	0.00	5.00	0.00	0.00	0.00
28.	Sandwiches	Sandwich / Vegetarian	0.00	0.00	5.00	0.00	25.00	0.00	0.00	0.00	5.00	0.00	0.00	5.00	0.00	5.00	0.00	0.00	0.00
29.	Sandwiches	Sandwich / Fish	0.00	0.00	5.00	0.00	0.00	0.00	0.00	0.00	5.00	25.00	0.00	5.00	0.00	5.00	0.00	0.00	0.00
30.	Sandwiches	Sandwich / Fish / Whole grain bread	50.00	0.00	5.00	0.00	0.00	0.00	0.00	0.00	5.00	25.00	0.00	5.00	0.00	5.00	0.00	0.00	0.00
31.	Sandwiches	Sandwich / Cheese	0.00	0.00	0.00	0.00	0.00	0.00	0.00	0.00	0.00	0.00	0.00	30.00	0.00	5.00	0.00	0.00	0.00
32.	Sandwiches	Sandwich / Cheese / Whole grain bread	50.00	0.00	0.00	0.00	0.00	0.00	0.00	0.00	0.00	0.00	0.00	30.00	0.00	5.00	0.00	0.00	0.00
33.	Tarts	Tart / Meat	0.00	0.00	5.00	0.00	0.00	10.00	0.00	0.00	10.00	0.00	5.00	5.00	10.00	20.00	0.00	0.00	0.00
34.	Tarts	Tart / Pork	0.00	0.00	5.00	0.00	0.00	0.00	10.00	0.00	10.00	0.00	5.00	5.00	10.00	20.00	0.00	0.00	0.00
35.	Tarts	Tart / Poultry	0.00	0.00	5.00	0.00	0.00	0.00	0.00	10.00	10.00	0.00	5.00	5.00	10.00	20.00	0.00	0.00	0.00
36.	Tarts	Tart / Vegetables	0.00	0.00	20.00	0.00	0.00	0.00	0.00	0.00	10.00	0.00	5.00	5.00	10.00	20.00	0.00	0.00	0.00
37.	Tarts	Tart / Fish	0.00	0.00	5.00	0.00	0.00	0.00	0.00	0.00	10.00	10.00	5.00	5.00	10.00	20.00	0.00	0.00	0.00
38.	Tarts	Tart / Cheese	0.00	0.00	0.00	0.00	0.00	0.00	0.00	0.00	10.00	0.00	5.00	15.00	10.00	20.00	0.00	0.00	0.00
39.	Tarts	Puff pastry tart / Meat	0.00	0.00	0.00	0.00	0.00	15.00	0.00	0.00	5.00	0.00	5.00	5.00	10.00	20.00	0.00	0.00	0.00
40.	Tarts	Puff pastry tart / Pork	0.00	0.00	0.00	0.00	0.00	0.00	15.00	0.00	5.00	0.00	5.00	5.00	10.00	20.00	0.00	0.00	0.00
41.	Tarts	Puff pastry tart / Pork / Meat	0.00	0.00	0.00	0.00	0.00	7,5	7,5	0.00	5.00	0.00	5.00	5.00	10.00	20.00	0.00	0.00	0.00
42.	Tarts	Puff pastry tart / Poultry	0.00	0.00	0.00	0.00	0.00	0.00	0.00	15.00	5.00	0.00	5.00	5.00	10.00	20.00	0.00	0.00	0.00
43.	Tarts	Puff pastry tart / Vegetables	0.00	0.00	15.00	0.00	0.00	0.00	0.00	0.00	5.00	0.00	5.00	5.00	10.00	20.00	0.00	0.00	0.00

44.	Tarts	Puff pastry tart / Fish	0.00	0.00	0.00	0.00	0.00	0.00	0.00	0.00	5.00	15.00	5.00	5.00	10.00	20.00	0.00	0.00	0.00
45.	Tarts	Puff pastry tart / Cheese	0.00	0.00	0.00	0.00	0.00	0.00	0.00	0.00	5.00	0.00	5.00	20.00	10.00	20.00	0.00	0.00	0.00
46.	Pastries / Desserts	Sweet tart	0.00	0.00	0.00	0.00	0.00	0.00	0.00	0.00	0.00	0.00	0.00	0.00	10.00	20.00	0.00	0.00	20.00
47.	Pastries / Desserts	Pastry	0.00	0.00	0.00	0.00	0.00	0.00	0.00	0.00	0.00	0.00	0.00	0.00	20.00	15.00	0.00	0.00	25.00
48.	Pastries / Desserts	Brioche / Viennoiserie	0.00	0.00	0.00	0.00	0.00	0.00	0.00	0.00	0.00	0.00	0.00	0.00	0.00	25.00	0.00	0.00	15.00
49.	Pastries / Desserts	Croissant dough / Viennoiserie	0.00	0.00	0.00	0.00	0.00	0.00	0.00	0.00	0.00	0.00	0.00	0.00	0.00	20.00	0.00	0.00	15.00
50.	Pastries / Desserts	Puff pastry dough / Viennoiserie	0.00	0.00	0.00	0.00	0.00	0.00	0.00	0.00	0.00	0.00	0.00	0.00	0.00	30.00	0.00	0.00	15.00
51.	Savoury Crêpes	Crêpe / Meat	5.00	0.00	5.00	0.00	0.00	25.00	0.00	0.00	5.00	0.00	20.00	10.00	5.00	0.00	0.00	0.00	0.00
52.	Savoury Crêpes	Crêpe / Pork	5.00	0.00	5.00	0.00	0.00	0.00	25.00	0.00	5.00	0.00	20.00	10.00	5.00	0.00	0.00	0.00	0.00
53.	Savoury Crêpes	Crêpe / Poultry	5.00	0.00	5.00	0.00	0.00	0.00	0.00	25.00	5.00	0.00	20.00	10.00	5.00	0.00	0.00	0.00	0.00
54.	Savoury Crêpes	Crêpe / Vegetables	5.00	0.00	30.00	0.00	0.00	0.00	0.00	0.00	5.00	0.00	20.00	10.00	5.00	0.00	0.00	0.00	0.00
55.	Pastries / Desserts	Crêpe / Sweet	0.00	0.00	0.00	0.00	0.00	0.00	0.00	0.00	0.00	0.00	0.00	0.00	10.00	5.00	0.00	0.00	20.00
56.	Sweets / Desserts	Ice cream	0.00	0.00	0.00	0.00	0.00	0.00	0.00	0.00	0.00	0.00	0.00	0.00	40.00	0.00	0.00	0.00	20.00
57.	Sweets / Desserts	Sorbet	0.00	0.00	0.00	0.00	0.00	0.00	0.00	0.00	0.00	0.00	0.00	0.00	0.00	0.00	0.00	0.00	20.00
58.	Breakfast cereal	Breakfast cereal	0.00	0.00	0.00	0.00	0.00	0.00	0.00	0.00	0.00	0.00	0.00	0.00	0.00	0.00	0.00	0.00	20.00
59.	Sweets / Desserts	Jam	0.00	0.00	0.00	0.00	0.00	0.00	0.00	0.00	0.00	0.00	0.00	0.00	0.00	0.00	0.00	0.00	80.00
60.	Sweets / Desserts	Fruit juice	0.00	0.00	0.00	0.00	0.00	0.00	0.00	0.00	0.00	0.00	0.00	0.00	0.00	0.00	0.00	0.00	20.00
61.	Fruits	Compote	0.00	0.00	0.00	90.00	0.00	0.00	0.00	0.00	0.00	0.00	0.00	0.00	0.00	0.00	0.00	0.00	10.00
62.	Sweets / Desserts	Chocolate bar	0.00	0.00	0.00	0.00	0.00	0.00	0.00	0.00	0.00	0.00	0.00	0.00	25.00	0.00	0.00	0.00	55.00

63.	Sweets / Desserts	Confectionery	0.00	0.00	0.00	0.00	0.00	0.00	0.00	0.00	0.00	0.00	0.00	0.00	5.00	0.00	0.00	0.00	80.00
64.	Sweets / Desserts	Biscuit	0.00	0.00	0.00	0.00	0.00	0.00	0.00	0.00	0.00	0.00	0.00	0.00	0.00	15.00	0.00	0.00	15.00
65.	Sweets / Desserts	Honey	0.00	0.00	0.00	0.00	0.00	0.00	0.00	0.00	0.00	0.00	0.00	0.00	0.00	0.00	0.00	0.00	80.00
66.	Sweets / Desserts	Chocolate	0.00	0.00	0.00	0.00	0.00	0.00	0.00	0.00	0.00	0.00	0.00	0.00	10.00	10.00	0.00	0.00	40.00
67.	Sweets / Desserts	Dessert cream	0.00	0.00	0.00	0.00	0.00	0.00	0.00	0.00	0.00	0.00	0.00	0.00	5.00	0.00	0.00	0.00	10.00
68.	Savoury Crêpes	Crêpe / Cheese	5.00	0.00	5.00	0.00	0.00	0.00	0.00	0.00	5.00	0.00	20.00	35.00	5.00	0.00	0.00	0.00	0.00
69.	Savoury Crêpes	Crêpe / Egg	5.00	0.00	5.00	0.00	0.00	0.00	0.00	0.00	30.00	0.00	20.00	10.00	5.00	0.00	0.00	0.00	0.00
70.	Savoury Crêpes	Crêpe	5.00	0.00	5.00	0.00	0.00	0.00	0.00	0.00	5.00	0.00	30.00	0.00	5.00	0.00	0.00	0.00	0.00
71.	Savoury Crêpes	Crêpe / Fish	5.00	0.00	5.00	0.00	0.00	0.00	0.00	0.00	5.00	25.00	25.00	5.00	5.00	0.00	0.00	0.00	0.00
72.	Baked Gratin-Type Dishes	Gratin / Meat	0.00	0.00	0.00	0.00	0.00	35.00	0.00	0.00	0.00	0.00	1.00	4.00	8.00	2.00	0.00	0.00	0.00
73.	Baked Gratin-Type Dishes	Gratin / Pork	0.00	0.00	0.00	0.00	0.00	0.00	35.00	0.00	0.00	0.00	1.00	4.00	8.00	2.00	0.00	0.00	0.00
74.	Baked Gratin-Type Dishes	Gratin / Poultry	0.00	0.00	0.00	0.00	0.00	0.00	0.00	35.00	0.00	0.00	1.00	4.00	8.00	2.00	0.00	0.00	0.00
75.	Baked Gratin-Type Dishes	Gratin / Vegetables	0.00	0.00	35.00	0.00	0.00	0.00	0.00	0.00	0.00	0.00	1.00	4.00	8.00	2.00	0.00	0.00	0.00
76.	Baked Gratin-Type Dishes	Gratin / Fish	0.00	0.00	0.00	0.00	0.00	0.00	0.00	0.00	0.00	35.00	1.00	4.00	8.00	2.00	0.00	0.00	0.00
77.	Baked Gratin-Type Dishes	Gratin / Starch / Meat	0.00	0.00	0.00	0.00	0.00	15.00	0.00	0.00	0.00	0.00	1.00	4.00	8.00	2.00	0.00	0.00	0.00
78.	Baked Gratin-Type Dishes	Gratin / Starch / Pork	0.00	0.00	0.00	0.00	0.00	0.00	15.00	0.00	0.00	0.00	1.00	4.00	8.00	2.00	0.00	0.00	0.00
79.	Baked Gratin-Type Dishes	Gratin / Starch / Poultry	0.00	0.00	0.00	0.00	0.00	0.00	0.00	15.00	0.00	0.00	1.00	4.00	8.00	2.00	0.00	0.00	0.00
80.	Baked Gratin-Type Dishes	Gratin / Starch / Vegetables	0.00	0.00	15.00	0.00	0.00	0.00	0.00	0.00	0.00	0.00	1.00	4.00	8.00	2.00	0.00	0.00	0.00
81.	Baked Gratin-Type Dishes	Gratin / Starch / Fish	0.00	0.00	0.00	0.00	0.00	0.00	0.00	0.00	0.00	15.00	1.00	4.00	8.00	2.00	0.00	0.00	0.00
82.	Baked Gratin-Type Dishes	Gratin / Starch	0.00	0.00	0.00	0.00	0.00	0.00	0.00	0.00	0.00	0.00	1.00	4.00	8.00	2.00	0.00	0.00	0.00
83.	Baked Gratin-Type Dishes	Potato gratin / Meat	0.00	30.00	0.00	0.00	0.00	15.00	0.00	0.00	0.00	0.00	1.00	4.00	8.00	2.00	0.00	0.00	0.00

84.	Baked Gratin-Type Dishes	Potato gratin / Pork	0.00	30.00	0.00	0.00	0.00	0.00	15.00	0.00	0.00	0.00	1.00	4.00	8.00	2.00	0.00	0.00	0.00
85.	Baked Gratin-Type Dishes	Potato gratin / Meat / Poultry	0.00	30.00	0.00	0.00	0.00	10.00	0.00	10.00	0.00	0.00	1.00	4.00	8.00	2.00	0.00	0.00	0.00
86.	Baked Gratin-Type Dishes	Potato gratin / Poultry	0.00	30.00	0.00	0.00	0.00	0.00	0.00	15.00	0.00	0.00	1.00	4.00	8.00	2.00	0.00	0.00	0.00
87.	Baked Gratin-Type Dishes	Potato gratin / Vegetables	0.00	30.00	15.00	0.00	0.00	0.00	0.00	0.00	0.00	0.00	1.00	4.00	8.00	2.00	0.00	0.00	0.00
88.	Baked Gratin-Type Dishes	Potato gratin / Fish	0.00	30.00	0.00	0.00	0.00	0.00	0.00	0.00	0.00	15.00	1.00	4.00	8.00	2.00	0.00	0.00	0.00
89.	Baked Gratin-Type Dishes	Potato gratin	0.00	30.00	0.00	0.00	0.00	0.00	0.00	0.00	0.00	0.00	1.00	4.00	8.00	2.00	0.00	0.00	0.00
90.	Baked Gratin-Type Dishes	Gratin / Vegetables / Meat	0.00	0.00	30.00	0.00	0.00	0.00	0.00	15.00	0.00	0.00	1.00	4.00	8.00	2.00	0.00	0.00	0.00
91.	Baked Gratin-Type Dishes	Gratin / Vegetables / Pork	0.00	0.00	30.00	0.00	0.00	0.00	0.00	15.00	0.00	0.00	1.00	4.00	8.00	2.00	0.00	0.00	0.00
92.	Dishes with dough / Pasta / Pâte	Pasta / Legumes	10.00	0.00	0.00	0.00	40.00	0.00	0.00	0.00	0.00	0.00	0.00	0.00	0.00	0.00	0.00	0.00	0.00
93.	Potato-based dishes	Tuber / Cheese	0.00	50.00	0.00	0.00	0.00	0.00	0.00	0.00	0.00	0.00	2.00	8.00	0.00	0.00	0.00	0.00	0.00
94.	Baked Gratin-Type Dishes	Gratin / Vegetables / Fish	0.00	0.00	20.00	0.00	0.00	0.00	0.00	0.00	0.00	15.00	1.00	4.00	8.00	2.00	0.00	0.00	0.00
95.	Baked Gratin-Type Dishes	Gratin / Tuber / Cheese	0.00	0.00	50.00	0.00	0.00	0.00	0.00	0.00	0.00	0.00	0.00	10.00	13.00	2.00	0.00	0.00	0.00
96.	Dishes with dough / Pasta / Pâte	Pasta / Fish / Poultry	0.00	0.00	0.00	0.00	0.00	0.00	0.00	15.00	0.00	15.00	0.00	0.00	0.00	0.00	0.00	0.00	0.00
97.	Spring rolls (Nem)	Spring roll / Fish	0.00	0.00	45.00	0.00	0.00	0.00	0.00	0.00	10.00	25.00	0.00	0.00	0.00	0.00	0.00	0.00	0.00

98.	Spring rolls (Nem)	Spring roll / Vegetables	0.00	0.00	70.00	0.00	0.00	0.00	0.00	0.00	10.00	0.00	0.00	0.00	0.00	0.00	0.00	0.00	0.00
99.	Spring rolls (Nem)	Spring roll / Poultry	0.00	0.00	45.00	0.00	0.00	0.00	0.00	25.00	10.00	0.00	0.00	0.00	0.00	0.00	0.00	0.00	0.00
100.	Spring rolls (Nem)	Spring roll / Meat	0.00	0.00	45.00	0.00	0.00	25.00	0.00	0.00	10.00	0.00	0.00	0.00	0.00	0.00	0.00	0.00	0.00
101.	Spring rolls (Nem)	Spring roll / Pork	0.00	0.00	45.00	0.00	0.00	0.00	25.00	0.00	10.00	0.00	0.00	0.00	0.00	0.00	0.00	0.00	0.00
102.	Spring rolls (Nem)	Spring roll / Fish / Poultry	0.00	0.00	45.00	0.00	0.00	0.00	0.00	12.00	15.00	12.00	0.00	0.00	0.00	0.00	0.00	0.00	0.00
103.	Potato-based dishes	Potatoes / Meat / Cheese	0.00	45.00	0.00	0.00	0.00	25.00	0.00	0.00	0.00	0.00	0.00	10.00	5.00	0.00	0.00	5.00	0.00
104.	Potato-based dishes	Potatoes / Pork / Cheese	0.00	45.00	0.00	0.00	0.00	0.00	25.00	0.00	0.00	0.00	0.00	10.00	5.00	0.00	0.00	5.00	0.00
105.	Dishes with dough / Pasta / Pâte	Pasta / Meat / Cheese	0.00	0.00	0.00	0.00	0.00	10.00	0.00	0.00	0.00	0.00	0.00	10.00	5.00	0.00	0.00	5.00	0.00
106.	Dishes with dough / Pasta / Pâte	Pasta / Pork / Cheese	0.00	0.00	0.00	0.00	0.00	0.00	10.00	0.00	0.00	0.00	0.00	10.00	5.00	0.00	0.00	5.00	0.00
107.	Pizzas	Pizza	0.00	4.00	8.00	0.00	0.00	2.00	2.00	4.00	4.00	4.00	0.00	10.00	4.00	0.00	0.00	4.00	4.00
108.	Fritters	Savoury fritter	0.00	0.00	0.00	0.00	0.00	0.00	0.00	0.00	0.00	0.00	0.00	0.00	0.00	5.00	0.00	0.00	0.00
109.	Fritters	Sweet fritter	0.00	0.00	0.00	0.00	0.00	0.00	0.00	0.00	0.00	0.00	0.00	0.00	0.00	5.00	0.00	0.00	15.00
110.	Spring rolls (Nem)	Spring roll / Cheese	0.00	0.00	0.00	0.00	0.00	0.00	0.00	0.00	10.00	0.00	0.00	25.00	0.00	0.00	0.00	0.00	0.00
111.	Meat-based dishes	Meat / Tuber / Vegetables	0.00	35.00	35.00	0.00	0.00	20.00	0.00	0.00	0.00	0.00	0.00	0.00	0.00	0.00	0.00	0.00	0.00
112.	Meat-based dishes	Pork / Tuber / Vegetables	0.00	35.00	35.00	0.00	0.00	0.00	20.00	0.00	0.00	0.00	0.00	0.00	0.00	0.00	0.00	0.00	0.00

113.	Meat-based dishes	Poultry / Tuber / Vegetables	0.00	35.00	35.00	0.00	0.00	0.00	0.00	20.00	0.00	0.00	0.00	0.00	0.00	0.00	0.00	0.00	0.00
114.	Vegetables-based dishes	Vegetables / Tuber	0.00	45.00	45.00	0.00	0.00	0.00	0.00	0.00	0.00	0.00	0.00	0.00	0.00	0.00	0.00	0.00	0.00
115.	Tarts	Pasta / Tart	0.00	0.00	0.00	0.00	0.00	0.00	0.00	0.00	5.00	0.00	0.00	0.00	0.00	30.00	0.00	0.00	0.00
116.	Meat-based dishes	Meat / Starch / Vegetables	0.00	0.00	35.00	0.00	0.00	20.00	0.00	0.00	0.00	0.00	0.00	0.00	0.00	0.00	0.00	0.00	0.00
117.	Meat-based dishes	Pork / Starch / Vegetables	0.00	0.00	35.00	0.00	0.00	0.00	20.00	0.00	0.00	0.00	0.00	0.00	0.00	0.00	0.00	0.00	0.00
118.	Meat-based dishes	Poultry / Starch / Vegetables	0.00	0.00	35.00	0.00	0.00	0.00	0.00	20.00	0.00	0.00	0.00	0.00	0.00	0.00	0.00	0.00	0.00
119.	Vegetables-based dishes	Starch / Vegetables	0.00	0.00	35.00	0.00	0.00	0.00	0.00	0.00	0.00	0.00	0.00	0.00	0.00	0.00	0.00	0.00	0.00
120.	Tuber-based dishes	Tuber / Egg	0.00	50.00	0.00	0.00	0.00	0.00	0.00	0.00	50.00	0.00	0.00	0.00	0.00	0.00	0.00	0.00	0.00
121.	Meat-based dishes	Burrito / Fajitas / Chili	5.00	0.00	20.00	0.00	20.00	20.00	0.00	0.00	0.00	0.00	0.00	0.00	0.00	0.00	0.00	0.00	0.00
122.	Sauces	Tzatziki (Yogurt / Cucumber)	0.00	0.00	60.00	0.00	0.00	0.00	0.00	0.00	0.00	0.00	0.00	0.00	20.00	0.00	0.00	0.00	0.00
123.	Meat-based dishes	Meat / Tuber	0.00	50.00	0.00	0.00	0.00	50.00	0.00	0.00	0.00	0.00	0.00	0.00	0.00	0.00	0.00	0.00	0.00
124.	Meat-based dishes	Pork / Tuber	0.00	50.00	0.00	0.00	0.00	0.00	50.00	0.00	0.00	0.00	0.00	0.00	0.00	0.00	0.00	0.00	0.00
125.	Vegetables-based dishes	Whole grain / Vegetables / Tuber / Cheese	25.00	15.00	20.00	0.00	0.00	0.00	0.00	0.00	4.00	0.00	5.00	10.00	2.00	1.00	0.00	0.00	0.00
126.	Fats	Butter / Oil	0.00	0.00	0.00	0.00	0.00	0.00	0.00	0.00	0.00	0.00	0.00	0.00	0.00	50.00	0.00	50.00	0.00
127.	Fruits	Fruit / Nut mix	0.00	0.00	0.00	50.00	0.00	0.00	0.00	0.00	0.00	0.00	0.00	0.00	0.00	0.00	50.00	0.00	0.00

128.	Meat-based dishes	Meat / Nuts	0.00	0.00	0.00	0.00	0.00	80.00	0.00	0.00	5.00	0.00	0.00	0.00	0.00	0.00	10.00	0.00	0.00
129.	Meat-based dishes	Pork / Nuts	0.00	0.00	0.00	0.00	0.00	0.00	80.00	0.00	5.00	0.00	0.00	0.00	0.00	0.00	10.00	0.00	0.00
130.	Meat-based dishes	Meat / Legumes	0.00	0.00	0.00	0.00	50.00	50.00	0.00	0.00	0.00	0.00	0.00	0.00	0.00	0.00	0.00	0.00	0.00
131.	Meat-based dishes	Pork / Legumes	0.00	0.00	0.00	0.00	50.00	0.00	50.00	0.00	0.00	0.00	0.00	0.00	0.00	0.00	0.00	0.00	0.00
132.	Meat-based dishes	Meat / Tuber	0.00	50.00	0.00	0.00	0.00	0.00	0.00	50.00	0.00	0.00	0.00	0.00	0.00	0.00	0.00	0.00	0.00
133.	Meat-based dishes	Pork / Tuber	0.00	50.00	0.00	0.00	0.00	0.00	0.00	50.00	0.00	0.00	0.00	0.00	0.00	0.00	0.00	0.00	0.00
134.	Whole grain-based dishes	Semi-wholegrain	50.00	0.00	0.00	0.00	0.00	0.00	0.00	0.00	0.00	0.00	0.00	0.00	0.00	0.00	0.00	0.00	0.00
135.	Whole grain-based dishes	Multigrain	30.00	0.00	0.00	0.00	0.00	0.00	0.00	0.00	0.00	0.00	0.00	0.00	0.00	0.00	0.00	0.00	0.00
136.	Sandwiches	Sandwich / Egg	0.00	0.00	0.00	0.00	0.00	0.00	0.00	0.00	30.00	0.00	0.00	0.00	0.00	0.00	0.00	0.00	0.00
137.	Sandwiches	Sandwich / Egg / Whole grain bread	50.00	0.00	0.00	0.00	0.00	0.00	0.00	0.00	30.00	0.00	0.00	0.00	0.00	0.00	0.00	0.00	0.00
138.	Sandwiches	Sandwich / Legumes	0.00	0.00	5.00	0.00	25.00	0.00	0.00	0.00	5.00	0.00	0.00	5.00	0.00	5.00	0.00	0.00	0.00
139.	Meat-based dishes	Meat / Pork	0.00	0.00	0.00	0.00	0.00	50.00	50.00	0.00	0.00	0.00	0.00	0.00	0.00	0.00	0.00	0.00	0.00
140.	Sweets / Desserts	Soda	0.00	0.00	0.00	0.00	0.00	0.00	0.00	0.00	0.00	0.00	0.00	0.00	0.00	0.00	0.00	0.00	10.00
141.	Fats	Butter / Sugar	0.00	0.00	0.00	0.00	0.00	0.00	0.00	0.00	0.00	0.00	0.00	0.00	0.00	30.00	0.00	0.00	55.00

Table S2. Number of participants excluded due to implausible energy intakes, by country, sex, and age group

	Europe	UK	France	Spain	Netherlands	Portugal	Hungary	Switzerland	Finland	Estonia
Women/Age group [18:45[	47	1	8	0	4	5	4	8	0	17
Women/Age group [45:65[	40	1	7	0	2	11	2	4	1	12
Women/Age group [65:80[	18	0	2	1	1	8	1	1	1	3
Men/Age group [18:45[	79	1	14	2	3	23	10	17	2	7
Men/Age group [45:65[	47	0	11	1	3	11	5	9	2	5
Men/Age group [65:80[	25	2	5	0	1	3	2	5	1	6
Total	256	5	47	4	14	61	24	44	7	50

Table S3. EAT-Lancet reference diet, with possible ranges, for an intake of 2,500 kcal/day

Food components	Macronutrient intake (possible range), g/day	Caloric intake, kcal/day
**Whole grains**		
Rice, wheat, corn, and other†	232 (total gains 0–60% of energy)	811
**Tubers or starchy vegetables**		
Potatoes and cassava	50 (0–100)	39
**Vegetables**		
All vegetables	300 (200–600)	
Dark green vegetables	100	23
Red and orange vegetables	100	30
Other vegetables	100	25
**Fruits**		
All fruit	200 (100–300)	126
**Dairy foods**		
Whole milk or derivative equivalents (eg, cheese)	250 (0–500)	153
**Protein sources**		
Beef and lamb	7 (0–14)	15
Pork	7 (0–14)	15
Chicken and other poultry	29 (0–58)	62
Eggs	13 (0–25)	19
Fish	28 (0–100)	40
Legumes		
Dry beans, lentils, and peas	50 (0–100)	172
Soy foods	25 (0–50)	112
Peanuts	25 (0–75)	142
Tree nuts	25	149
**Added fats**		
Palm oil	6.8 (0–6.8)	60
Unsaturated oils	40 (20–80)	354
Dairy fats (included in milk)	0	0
Lard or tallow	5 (0–5)	36
**Added sugars**		
All sweeteners	31 (0–31)	120

Willett W, et al. Food in the Anthropocene: the EAT-Lancet Commission on healthy diets from sustainable food systems. Lancet. 2019;393(10170):447-492.

World Index for Sustainability and Health

The World Index for Sustainability and Health (WISH) was developed to assess the healthiness and environmental sustainability of the diet based on EAT-Lancet recommendations. It comprises 13 food components classified as neutral, protective or negative for human and planetary health. Each food component is assessed by a quantitative score, using reference values in grams, which limits the independent individual assessment of total energy intake. The WISH index excludes tubers and starchy vegetables on the argument that they are not in the WHO and Global Burden of Disease study. It was developed using data collected through repeated 24-hour recall from a sample of 396 adults in Vietnam (urban area). The WISH score has been linked to certain health outcomes, such as decreased depression levels and improved cognitive performance.

The following formula is applied to compute the score of an intake between the lower recommended intake and the recommended intake:

$$WISH = 10 * \frac{reported\ intake - lower\ recommended\ intake}{recommended\ intake - lower\ recommended\ intake}$$

On the other hand, the score between the recommended intake and the upper recommended intake is calculated according to the following formula:

$$WISH = 10 * \frac{(upper\ recommended\ intake - recommended\ intake) - (reported\ intake - recommended\ intake)}{upper\ recommended\ intake - recommended\ intake}$$

Saturated oils and added sugars are scored as a bivariate component: 10 points are allocated for those consumptions within the recommended range and zero points are given for those exceeding this recommendation. The total score is calculated by adding the score of each food component, and ranges between 0 up to 130 points. Moreover, the authors propose four sub-scores derived from the WISH index: healthy, less healthy, low environmental impact and high environmental impact.

The following table presents the food components of WISH, proving examples of food items and a more detailed explanation of the scoring system.

Table S4. World Index for Sustainability and Health

Food components	Food items	Scoring
**Whole Grains**	Rice, wheat and corn (bran, germ, and endosperm in their natural proportion) or whole grain products in the form of breakfast cereals, bread, pasta, biscuits, muffins, tortillas, pancakes, and other sources.	≥125 g/d = 10 points.<br><100 g/d = 0 point.<br>100–125 g/d = 0–10 points.
**Vegetables**	Fresh, frozen, cooked, canned, or dried vegetables, and excludes legumes and salted or pickled vegetables, juices, nuts, and seeds, and starchy vegetables such as potatoes or corn.	≥300 g/d = 10 points.<br><200 g/d = 0 point.<br>200–300 g/d = 0–10 points.
**Fruits**	Fresh, frozen, cooked, canned, or dried fruits but excluding fruit juices, salted or pickled fruits and nuts or seeds.	≥200 g/d = 10 points.<br><100 g/d = 0 point.<br>100–200 g/d = 0–10 points.
**Dairy Foods**	Whole or skimmed milk or derivative equivalents (e.g., cheese, yoghurt or curd), excluding butter and cream.	250–500 g/d = 10 points.<br>0–250 g/d = 0–10 points.<br>>500 = 0 point.
**Red Meat**	Beef, pork, lamb, and goat including red meat in its processed form but excluding poultry, fish, eggs.	≤14 g/d = 10 points.<br>>28 g/d = 0 point.<br>14–28 g/d = 0–10 points, inversely.
**Fish**	Fish and shellfish such as mussels and shrimp.	28–100 g/d = 10 points.<br>0–28 g/d = 0–10 points.<br>>100 = 0 point.
**Eggs**	Eggs from chickens and ducks, but excludes fish eggs.	≤13 g/d = 10 points.<br>>25 g/d = 0 point.<br>13–25 g/d = 0–10 points, inversely.
**Chicken and Other Poultry**	Meat from chicken, ducks, geese etc.	≤29 g/d = 10 points.<br>>58 g/d = 0 point.<br>29–58 g/d = 0–10 points, inversely.
**Legumes**	Fresh, frozen, cooked, canned, or dried legumes, beans and lentils, but also includes soy foods and peas.	0-75 g/d = 0–10 points.<br>≥75 g/d = 10 points.
**Nuts**	Tree nuts and ground nuts (including peanuts).	50–75 g/d = 10 points.<br>0-50 g/d = 0–10 points.<br>≥75 g/d = 0 point.
**Unsaturated Oils**	Olive, soybean, rapeseed, sunflower and peanut oil.	≥40 g/d = 10 points.<br><20 g/d = 0 point.<br>20–40 g/d = 0–10 points.<br>> 80 g/d = 0 point.
**Saturated Oils**	Dairy fats, lard or tallows, and palm oil.	≤11.8 g/d= 10 points<br>> 11.8 g/d = 0 point.
**Added Sugars**	Added sugar and sugar sweetened beverages.	≤31 g/d= 10 points<br>> 31 g/d = 0 point.

Trijsburg L, Talsma EF, Crispim SP, Garrett J, Kennedy G, de Vries JHM, Brouwer ID. Method for the Development of WISH, a Globally Applicable Index for Healthy Diets from Sustainable Food Systems. Nutrients. 2020;13(1):93.

EAT-Lancet Index

The EAT-Lancet Index (ELI) was developed using data from 22,421 Swedish adults aged 45 to 73 who participated in the Malmö Diet and Cancer cohort between 1991 and 1996, which included food diaries and food frequency questionnaires. The ELI covers 14 food components categorized as emphasized and restricted foods, with daily gram quantities assessed through a semi-quantitative scoring system: from 0 to 3 points. This index has been related with reduced all-cause mortality, decreased mortality from cancer and cardiovascular diseases. Furthermore, it was observed that higher adherence to this index is associated with a lower risk of type 2 diabetes in Swedish adults, and this relationship is dose-response and independent of genetic susceptibility. The following table presents the food components of the ELI, proving examples of food items and a more detailed explanation of the scoring.

Table S5. EAT-Lancet Index

Food components	Food items	Scoring			
		3 points	2 points	1 point	0 points
**Vegetables**	All vegetables except legumes.	>300	200–300	100–200	<100
**Fruits**	Fruits and berries.	>200	100–200	50–100	<50
**Unsaturated oils**	All plant oils and plant margarines.	>40	20–40	10–20	<10
**Legumes**	Dry beans, lentils, peas, soy. Peas, lentils, beans, tofu, soy containing meat replacement products.	>75	37.5–75	18.75–37.5	<18.75
**Nuts**	Peanuts or tree nuts. All nuts and seeds including peanuts, nut mixes such as almond paste.	>50	25–50	12.5–25	<12.5
**Whole grains**	Whole grains (e.g., cereals, rolled oats, crispbread) and whole grain foods (e.g., pastas, doughs and breads).	>232	116–232	58–116	<58
**Fish**	Fatty fish, lean fish, fish products, shellfish.	>28	14–28	7–14	<7
**Beef and lamb**	Beef, lamb, minced meat with pork and lamb, processed meats with beef and lamb including sausages.	<7	7–14	14–28	>28
**Pork**	Pork, minced meat of pork, processed meats with pork including ham, bacon, and sausages.	<7	7–14	14–28	>28
**Poultry**	Chicken, turkey, duck, goose, and other poultry.	<29	29–58	58–116	>116
**Eggs**	Boiled eggs, fried eggs and eggs in dishes such as omelet and pie.	<13	13–25	25–50	>50
**Dairy**	Whole milk or derivative equivalents. Regular milk, low-fat milk, yoghurt and other fermented milk products, hard cheese, soft cheese, cream, butter, butter-based spreads. All dairy foods were expressed as of milk equivalents. Equivalency factor: whole milk 1.0, Cheese 5.0, cream 2.7 and butter 6.5.	<250	250–500	500–1000	>1000
**Potatoes**	Boiled potatoes, fried potatoes, deep fried potatoes, potatoes included in dishes such as potato salad.	<50	50–100	100–200	>200
**Added sugar**	Sucrose and monosaccharides except sugars in fruits and vegetable.	<31	31–62	62–124	>124

Stubbendorff A, Sonestedt E, Ramne S, Drake I, Hallström E, Ericson U. Development of an EAT-Lancet index and its relation to mortality in a Swedish population. Am J Clin Nutr. 2022;115(3):705-716.

EAT-Lancet diet index

Data from 29,210 French adults (75% women) who participated in the NutriNet-Santé study were used to developed the EAT-Lancet Dietary Index (ELD-I). The ELD-I comprehensively covers 14 food components using cut-off points adapted from those proposed in the EPIC (European Prospective Investigation into Cancer and Nutrition)-Oxford study. The ELD-I is based on a quantitative scoring system that was designed to more accurately capture the variability in food consumption patterns, incorporating individual's total energy intake and standardizing it to a daily intake of 2,500 kcal. The computation results in a continuous variable being either positive or negative. As the score increases, the individual's diet aligns more closely with the EAT-Lancet recommendations. Moreover, the validity of the ELD-I has been explored, with results confirming its ability to measure healthiness and sustainability of diets.

The following table presents the food components of the ELD-I, proving examples of food items and a more detailed explanation of the scoring.

Table S6. EAT-Lancet diet index

Food components	Food items	Scoring computation
**Whole grains**	Rice, wheat, corn, and other.	$$ELD-I_i = \frac{100\times\left\{\sum_{component\ i=1}^{14}\frac{a_i\times\left(cut\text{-}off_i-\frac{consumption_{ij}\times 2500}{Energy\ intake\ _j}\right)}{cut\text{-}off_i}\right\}}{14}$$ Where *i* referred to on the 14 food groups and j is the individual. $\alpha_i = 1$ for component to limit and $\alpha_i = -1$ for component to promote.
**Potatoes and tuber**	Potatoes and cassava.	
**Vegetables**	All non-starchy vegetables.	
**Fruits**	All fruits.	
**Dairy foods**	Whole milk or derivative equivalents (e.g., cheese).	
**Beef, lamb, pork**	Red meats of beef, lamb and pork and processed meats.	
**Chicken and poultry**	Chicken and other poultry (e.g., turkey, duck, and quail).	
**Eggs**	Eggs from chicken, duck, and goose.	
**Fish**	Fish and shellfish (e.g., mussels and shrimps).	
**Legumes**	Dry beans, lentils, peas, soy foods.	
**Nuts**	Almonds, hazelnuts, pistachios and walnuts.	
**Saturated oil**	Dairy fats (e.g., cream and butter), lard or tallow.	
**Unsaturated oils**	Olive, soybean, rapeseed, sunflower, and peanut oil.	
**All sweet**	Added sugars and sugar-sweetened beverages.	

Kesse-Guyot E, Rebouillat P, Brunin J, Langevin B, Allès B, Touvier M, et al. Environmental and nutritional analysis of the EAT-Lancet diet at the individual level: insights from the NutriNet-Santé study. J Clean Prod. 2021;296:126555.

Table S7. Survey respondent distribution by country, sex, and age group: Counts, within-group proportions, and country weights

	Europe	UK	France	Spain	Netherlands	Portugal	Hungary	Switzerland	Finland	Estonia
Total – Count	16083	519	2074	929	1733	3703	1032	2013	1481	2599
Total – Survey Proportion	100%	3.23%	12.90%	5.78%	10.78%	23.02%	6.42%	12.52%	9.21%	16.16%
Total – Country Weight	100%	28.30%	27.91%	20.52%	7.50%	4.59%	4.43%	3.77%	2.41%	0.58%
Women – Count	9025	301	1217	531	860	1970	521	1115	778	1732
Women – Survey Proportion	100%	3.34%	13.48%	5.88%	9.53%	21.83%	5.77%	12.35%	8.62%	19.19%
Women – Country Weight	100%	28.13%	28.20%	20.36%	7.38%	4.74%	4.52%	3.70%	2.37%	0.60%
Men – Count	7058	218	857	398	873	1733	511	898	703	867
Men – Survey Proportion	100%	3.09%	12.14%	5.64%	12.37%	24.55%	7.24%	12.72%	9.96%	12.28%
Men – Country Weight	100%	28.47%	27.62%	20.69%	7.62%	4.44%	4.34%	3.83%	2.44%	0.56%
Age group [18:45[– Count	6397	193	761	449	433	1730	236	903	508	1184
Age group [18:45[– Survey Proportion	100%	3.02%	11.90%	7.02%	6.77%	27.04%	3.69%	14.12%	7.94%	18.51%
Age group [18:45[– Country Weight	100%	29.10%	27.52%	20.62%	7.18%	4.36%	4.51%	3.80%	2.31%	0.59%
Age group [45:65[– Count	5738	195	809	217	695	1322	272	776	553	899
Age group [45:65[– Survey Proportion	100%	3.40%	14.10%	3.78%	12.11%	23.04%	4.74%	13.52%	9.64%	15.67%
Age group [45:65[– Country Weight	100%	27.39%	28.04%	21.08%	7.80%	4.68%	4.28%	3.83%	2.34%	0.56%

Age group [65:80[– Count	3948	131	504	263	605	651	524	334	420	516
Age group [65:80[– Survey Proportion	100%	3.32%	12.77%	6.66%	15.32%	16.49%	13.27%	8.46%	10.64%	13.07%
Age group [65:80[– Country Weight	100%	28.07%	28.65%	19.14%	7.73%	5.01%	4.50%	3.54%	2.78%	0.60%
Women/Age group [18:45[– Count	3713	126	451	286	209	973	108	523	275	762
Women/Age group [18:45[– Survey Proportion	100%	3.39%	12.15%	7.70%	5.63%	26.21%	2.91%	14.09%	7.41%	20.52%
Women/Age group [18:45[– Country Weight	100%	29.06%	27.89%	20.42%	7.15%	4.46%	4.42%	3.76%	2.25%	0.57%
Women/Age group [45:65[– Count	3200	107	474	113	356	685	150	422	276	617
Women/Age group [45:65[– Survey Proportion	100%	3.34%	14.81%	3.53%	11.13%	21.41%	4.69%	13.19%	8.63%	19.28%
Women/Age group [45:65[– Country Weight	100%	27.34%	28.29%	20.88%	7.65%	4.84%	4.36%	3.75%	2.31%	0.58%
Women/Age group [65:80[– Count	2112	68	292	132	295	312	263	170	227	353
Women/Age group [65:80[– Survey Proportion	100%	3.22%	13.83%	6.25%	13.97%	14.77%	12.45%	8.05%	10.75%	16.71%
Women/Age group [65:80[– Country Weight	100%	27.43%	28.75%	19.22%	7.43%	5.20%	5.02%	3.49%	2.78%	0.70%
Men/Age group [18:45[– Count	2684	67	310	163	224	757	128	380	233	422
Men/Age group [18:45[– Survey Proportion	100%	2.50%	11.55%	6.07%	8.35%	28.20%	4.77%	14.16%	8.68%	15.72%
Men/Age group [18:45[– Country Weight	100%	29.14%	27.15%	20.82%	7.20%	4.27%	4.60%	3.84%	2.36%	0.60%
Men/Age group [45:65[– Count	2538	88	335	104	339	637	122	354	277	282
Men/Age group [45:65[– Survey Proportion	100%	3.47%	13.20%	4.10%	13.36%	25.10%	4.81%	13.95%	10.91%	11.11%

Men/Age group [45:65[– Country Weight	100%	27.43%	27.79%	21.29%	7.95%	4.51%	4.20%	3.92%	2.37%	0.54%
Men/Age group [65:80[– Count	1836	63	212	131	310	339	261	164	193	163
Men/Age group [65:80[– Survey Proportion	100%	3.43%	11.55%	7.14%	16.88%	18.46%	14.22%	8.93%	10.51%	8.88%
Men/Age group [65:80[– Country Weight	100%	28.80%	28.54%	19.05%	8.07%	4.79%	3.90%	3.59%	2.78%	0.48%

Table S8. Weighted proportions

	Sex		Age			Education	
**Country**	**Women**	**Men**	**18-44**	**45-64**	**65-80**	**Lower**	**Higher**
Europe	50.84	49.16	46.72	35.52	17.76	58.02	41.98
United Kingdom	50.56	49.44	46.89	34.92	18.18	n/a	n/a
France	51.34	48.66	46.32	36.16	17.52	56.65	43.35
Spain	50.39	49.61	48.87	34.86	16.27	58.24	41.76
The Netherlands	50.07	49.93	43.96	36.88	19.16	52.15	47.85
Portugal	52.48	47.52	44.43	36.28	19.30	77.29	22.71
Hungary	51.77	48.23	46.59	34.79	18.62	63.50	36.50
Switzerland	50.04	49.96	46.53	36.56	16.91	50.97	49.03
Finland	50.19	49.81	44.21	34.99	20.80	n/a	n/a
Estonia	53.01	46.99	47.88	34.50	17.62	43.66	56.34

Table S9. Sex-specific mean consumption of PHD food groups across countries (g/day).

Food group	Sex	UK		France		Spain		The Netherlands		Portugal		Hungary		Switzerland		Finland		Estonia	
		M	SD	M	SD	M	SD	M	SD	M	SD	M	SD	M	SD	M	SD	M	SD
Whole grains	Women	36.7	50.0	20.8	42.1	34.2	48.3	83.9	88.1	22.7	50.7	75.9	86.3	44.2	57.1	117.3	102.1	42.9	51.5
	Men	44.2	54.9	16.8	35.8	37.1	49.0	90.1	90.2	12.7	37.5	62.6	78.5	46.2	59.7	130.3	109.3	32.0	45.3
Tubers	Women	81.4	80.3	81.8	83.4	66.7	71.2	69.7	82.4	133.9	119.2	83.8	95.1	66.6	83.4	96.4	92.2	147.4	134.9
	Men	86.4	72.2	85.3	84.0	70.3	70.5	77.7	88.1	145.4	124.2	91.1	91.8	61.8	78.1	105.7	94.2	185.1	150.8
Vegetables	Women	245.9	194.7	319.1	223.1	228.5	173.8	251.8	239.0	270.2	175.8	246.2	158.4	281.7	190.4	283.5	171.8	279.4	211.1
	Men	184.6	171.2	240.3	178.1	191.9	144.2	189.4	161.8	218.4	149.0	220.2	135.0	197.4	148.9	203.3	145.4	217.2	168.7
Fruits	Women	176.5	175.6	277.9	211.3	319.6	236.2	210.4	187.9	317.6	246.7	216.7	192.5	255.9	227.9	262.4	233.5	429.5	399.3
	Men	124.5	165.1	217.5	193.3	265.8	215.4	148.8	148.9	245.9	217.5	132.1	212.2	167.0	192.4	154.9	168.0	245.0	302.9
Legumes	Women	48.3	72.1	21.1	38.0	49.7	60.3	29.2	55.3	11.8	28.9	25.1	45.4	21.9	50.0	16.3	35.7	2.3	13.4
	Men	45.4	76.5	17.8	35.8	55.3	66.2	30.1	55.2	14.0	32.4	21.9	41.4	15.8	39.3	12.8	32.9	2.3	12.0
Red meat	Women	83.6	76.8	107.3	75.2	93.4	85.7	73.5	70.5	99.8	103.2	107.9	74.5	73.7	75.5	88.5	74.7	124.1	98.4
	Men	99.7	94.0	119.0	80.9	119.9	81.8	89.3	68.7	140.0	119.0	137.9	76.0	99.2	82.0	135.8	93.2	165.9	102.1
Beef and lamb	Women	53.1	61.6	57.0	62.0	37.1	61.6	38.1	50.8	49.5	77.6	15.8	26.1	38.3	58.8	30.1	42.9	39.4	56.6
	Men	58.4	79.5	64.9	71.6	45.0	67.0	43.3	49.2	71.0	94.5	21.2	29.8	47.8	63.9	42.3	58.5	53.2	61.9
Pork	Women	30.5	41.3	50.3	49.2	56.2	56.9	35.4	51.8	50.2	74.3	92.1	69.1	35.5	50.9	58.4	62.9	84.7	87.3
	Men	41.4	53.3	54.1	48.9	74.8	65.6	46.0	52.9	69.0	82.8	116.6	73.7	51.4	57.3	93.6	77.2	112.7	92.5
Poultry	Women	84.1	98.5	44.4	55.4	69.2	82.2	30.7	48.7	82.6	107.7	75.6	81.1	29.6	53.9	47.8	66.0	53.6	86.2
	Men	91.1	102.8	38.2	49.6	65.0	85.6	34.4	52.1	84.6	105.3	71.3	86.5	35.8	64.9	45.7	66.1	52.1	83.4
Eggs	Women	40.2	50.6	22.5	34.6	26.0	33.5	25.5	40.0	22.9	40.9	26.1	36.2	18.4	36.3	27.6	44.7	35.8	46.8
	Men	41.6	55.3	20.0	30.0	29.3	38.3	20.3	32.5	20.2	39.1	26.9	36.6	16.3	32.7	23.9	36.9	33.3	45.6
Fish and seafood	Women	43.4	52.5	46.3	59.0	87.6	87.1	21.8	47.3	81.3	96.6	13.5	38.2	26.2	53.0	41.9	61.0	43.9	72.5
	Men	40.7	58.2	40.5	53.0	71.5	75.6	14.7	35.4	84.8	94.2	11.3	32.9	22.8	48.3	40.6	64.4	40.1	70.1
Dairy	Women	292.3	228.1	264.3	222.4	571.0	312.5	394.8	312.3	385.8	315.0	250.1	241.7	271.1	209.9	491.6	333.0	323.3	260.1
	Men	308.9	227.8	208.7	179.2	430.8	244.5	359.4	288.8	259.9	228.5	184.9	188.8	224.0	188.3	463.4	330.5	304.7	271.5
Nuts	Women	11.1	23.4	3.4	10.2	6.9	51.7	16.3	24.6	3.0	11.4	4.5	12.7	6.2	14.5	5.7	14.7	3.8	13.6
	Men	6.7	15.4	2.9	8.4	3.9	15.7	17.6	27.4	2.7	10.8	3.8	12.4	4.6	13.4	4.1	14.6	2.9	12.6
Unsaturated oils	Women	6.5	8.6	16.8	14.2	31.5	15.4	21.4	16.5	19.3	12.2	26.8	14.9	15.7	15.9	27.5	18.9	14.6	13.9
	Men	6.9	9.4	13.8	11.9	28.1	13.5	24.0	18.0	18.9	11.4	28.1	16.8	14.0	15.1	31.1	22.0	17.1	14.4
Saturated fats	Women	40.1	27.6	48.6	35.8	18.2	22.2	29.2	21.8	22.5	22.1	49.0	33.5	47.5	37.6	49.9	38.8	40.4	34.7
	Men	36.3	24.8	42.8	32.5	17.1	18.7	26.2	20.3	19.7	19.5	38.9	30.1	42.7	32.3	45.3	35.7	37.5	37.7
Added sugars	Women	82.2	65.9	72.7	62.5	57.6	39.4	58.3	43.9	44.7	38.1	57.8	44.0	86.6	90.5	68.6	50.0	71.1	58.5
	Men	77.4	53.0	70.0	57.0	55.4	37.9	60.1	49.0	43.5	38.0	48.5	41.9	86.8	93.6	65.9	56.1	65.9	55.7

Table S10. Age-specific mean consumption of PHD food groups across countries (g/day).

Food group	Age	UK		France		Spain		The Netherlands		Portugal		Hungary		Switzerland		Finland		Estonia	
		M	SD	M	SD	M	SD	M	SD	M	SD	M	SD	M	SD	M	SD	M	SD
Whole grain	[18:45[	35.0	47.5	15.5	33.2	36.0	45.6	84.4	92.2	17.3	41.6	68.6	91.2	43.1	60.2	111.2	107.8	34.0	47.1
	[45:65[	42.8	56.6	22.5	45.4	35.0	51.7	89.0	90.8	18.3	46.0	66.4	71.9	45.9	57.7	130.9	98.6	39.4	49.2
	[65:80[	50.0	55.6	19.9	39.3	35.5	51.0	89.2	78.6	18.7	51.2	77.4	80.2	49.7	54.8	138.4	110.9	44.8	52.8
Tubers	[18:45[	75.9	69.0	71.6	70.8	68.7	72.3	60.3	78.5	121.2	102.8	86.4	87.8	59.8	79.2	92.9	90.5	155.0	144.9
	[45:65[	86.3	84.2	87.1	86.7	64.3	65.3	79.8	88.4	150.3	130.6	84.1	97.3	62.8	79.9	98.3	92.1	175.4	148.1
	[65:80[	99.9	76.7	107.4	101.2	76.9	77.3	92.8	89.7	160.8	137.9	95.4	100.3	79.1	85.3	123.0	98.1	172.6	130.3
Vegetables	[18:45[	172.8	153.9	213.2	167.3	179.4	144.1	215.6	230.7	218.6	147.2	213.9	139.8	223.9	168.3	236.5	160.2	237.8	183.2
	[45:65[	244.7	201.4	319.6	213.8	243.2	173.4	222.7	193.0	258.2	164.6	247.4	157.5	244.7	180.2	249.2	161.1	262.2	207.6
	[65:80[	269.9	205.5	379.4	222.9	232.9	163.3	228.2	169.8	284.0	194.9	257.2	144.5	271.9	183.0	249.3	176.8	260.1	197.9
Fruits	[18:45[	108.6	138.3	200.7	174.1	225.1	194.0	156.6	148.2	229.2	206.2	119.2	123.6	168.9	186.5	169.6	192.7	321.3	373.5
	[45:65[	168.6	175.3	272.3	223.6	326.7	224.4	173.6	181.5	300.7	241.5	213.3	263.5	231.7	225.3	213.4	207.7	359.7	370.1
	[65:80[	225.6	211.3	325.8	208.2	424.0	254.0	244.1	189.4	376.4	255.3	248.1	213.8	285.1	241.1	284.5	229.7	368.2	351.0
Legumes	[18:45[	45.9	74.3	16.2	33.5	51.0	61.4	27.0	47.3	10.7	27.4	19.5	39.8	15.7	39.8	18.0	43.1	2.3	12.8
	[45:65[	51.0	76.8	21.7	39.9	50.9	64.4	31.1	62.1	14.8	33.9	25.4	42.5	22.0	51.3	11.5	23.5	2.6	13.0
	[65:80[	41.5	69.3	23.8	38.5	60.4	66.6	33.0	57.9	14.2	30.8	30.2	52.4	20.9	43.7	12.4	27.5	1.9	12.1
Red meat	[18:45[	104.7	92.1	110.1	78.0	106.8	81.5	71.1	65.9	128.5	115.0	117.8	73.8	85.6	79.6	112.6	89.1	142.7	104.3
	[45:65[	73.1	76.7	116.9	79.6	111.4	87.9	88.9	72.8	119.0	112.0	124.4	78.8	85.3	81.0	114.5	87.1	144.8	100.9
	[65:80[	93.3	81.0	112.5	75.8	95.2	87.1	90.7	70.8	96.5	105.7	130.0	79.3	91.6	77.7	106.8	85.4	144.7	99.6
Beef and lamb	[18:45[	64.3	76.8	59.0	67.9	38.8	60.4	35.5	45.0	65.2	88.9	18.7	25.8	42.7	62.1	41.7	56.4	48.3	58.8
	[45:65[	41.9	60.1	62.9	66.8	45.9	71.7	44.0	53.6	59.8	86.6	18.0	29.7	42.7	61.9	32.5	48.1	45.4	62.5
	[65:80[	59.9	71.2	61.5	64.6	37.3	59.3	46.0	53.0	46.9	80.3	18.4	30.3	44.7	59.7	30.5	45.5	40.5	55.0
Pork	[18:45[	40.3	55.1	51.1	49.1	68.0	61.2	35.5	50.2	63.3	79.2	99.1	70.0	42.8	54.2	70.9	69.4	94.4	92.9
	[45:65[	31.2	42.4	54.0	49.9	65.5	62.0	44.8	55.8	59.2	81.4	106.4	73.9	42.6	52.9	82.0	75.4	99.4	89.4
	[65:80[	33.4	35.6	51.0	47.2	57.9	64.1	44.6	50.7	49.5	73.3	111.6	74.7	46.9	60.0	76.2	73.6	104.2	88.1
Poultry	[18:45[	107.0	108.5	41.3	51.4	79.9	90.7	39.1	57.6	99.8	116.5	82.6	91.1	40.0	65.0	51.3	67.6	61.1	88.7
	[45:65[	76.9	91.0	43.7	55.3	57.2	76.5	29.0	44.7	76.7	99.5	67.7	77.0	30.7	59.0	48.0	67.1	46.9	80.8
	[65:80[	58.0	86.9	36.9	50.2	50.2	71.2	24.6	40.6	58.9	87.8	61.6	74.2	16.9	38.9	34.9	59.2	42.5	79.9
Eggs	[18:45[	38.4	48.7	20.6	32.5	27.5	34.4	23.8	37.6	23.3	43.4	28.7	40.3	17.6	36.8	28.7	42.7	35.5	47.9
	[45:65[	46.8	63.2	21.2	32.7	26.1	35.3	21.4	36.6	20.9	38.4	22.1	31.7	17.5	34.8	24.2	41.2	35.4	46.9
	[65:80[	35.8	39.4	23.1	32.0	31.6	41.5	23.7	33.9	19.2	34.9	29.2	33.7	16.5	26.9	22.1	36.5	30.8	40.0
Fish and seafood	[18:45[	35.6	52.8	36.0	49.2	71.4	77.5	14.8	33.6	72.6	86.8	10.5	31.9	22.7	45.7	37.8	57.4	32.1	59.5

	[45:65[	43.5	52.8	47.9	60.5	85.2	86.6	18.4	45.2	89.8	98.9	14.5	38.9	28.3	57.3	41.2	64.3	51.1	82.7
	[65:80[	56.0	63.8	54.1	61.6	92.3	82.3	26.1	50.9	94.0	105.2	13.4	38.4	21.4	48.3	48.6	70.1	51.6	73.8
Dairy	[18:45[	253.9	200.0	220.5	188.1	486.8	289.1	329.5	274.9	328.7	270.8	247.3	232.5	246.3	211.9	478.2	342.4	323.8	261.6
	[45:65[	308.2	224.3	244.8	211.9	499.7	264.7	402.0	319.6	313.3	283.6	192.2	212.9	246.2	190.8	481.0	316.2	294.2	258.3
	[65:80[	406.0	264.7	265.9	225.1	549.6	333.8	438.5	306.6	343.5	313.9	196.4	192.5	254.2	190.4	470.4	336.2	329.4	287.8
Nuts	[18:45[	9.2	19.7	2.3	7.6	2.3	6.4	15.5	23.5	2.9	11.2	4.1	13.1	4.8	13.4	5.6	16.1	3.6	12.7
	[45:65[	9.9	21.9	4.2	11.0	5.2	15.6	18.6	27.1	3.2	11.8	4.8	12.5	6.4	15.4	4.4	13.1	3.8	15.5
	[65:80[	6.4	16.3	3.3	9.8	15.3	91.1	17.0	29.1	2.1	9.6	3.2	11.3	5.1	12.2	4.5	14.0	1.9	8.6
Unsaturated oils	[18:45[	6.6	8.0	12.1	11.0	27.4	14.0	21.0	16.1	18.5	11.3	27.4	14.4	13.9	13.7	26.1	17.9	15.3	13.1
	[45:65[	6.9	9.6	17.3	14.1	32.0	14.7	23.6	18.1	19.3	12.0	28.0	17.8	15.3	16.8	31.0	21.4	16.4	14.9
	[65:80[	6.8	10.0	20.1	14.7	32.4	15.1	24.8	18.1	20.1	12.5	26.4	15.3	16.2	17.2	33.1	23.3	15.9	15.5
Saturated fats	[18:45[	33.4	20.7	52.1	36.2	19.6	21.3	27.8	20.7	24.4	20.8	44.8	30.4	40.5	29.7	46.1	38.7	40.7	36.8
	[45:65[	39.5	28.0	41.6	32.7	17.5	21.2	27.4	21.4	20.1	21.5	44.7	35.1	46.2	37.4	46.4	33.4	38.5	37.2
	[65:80[	48.1	32.4	37.7	29.1	12.3	15.4	28.0	21.4	15.5	18.8	41.6	31.6	55.1	41.1	52.8	40.1	35.3	32.0
Added sugars	[18:45[	78.2	60.6	85.4	64.9	68.0	41.7	63.3	49.3	54.3	41.6	59.0	46.1	94.7	101.4	66.5	51.9	72.9	59.3
	[45:65[	77.1	59.0	61.1	53.6	46.9	31.6	56.4	45.4	38.3	33.7	51.3	41.1	80.3	80.9	70.3	53.6	64.8	56.4
	[65:80[	89.2	59.5	55.6	49.4	42.2	32.0	55.1	41.0	31.5	30.5	42.6	37.3	78.6	85.8	63.6	54.9	64.8	52.2

Table S11. Dominance analysis of food group consumption (g/day) on the WISH index.

Food group	EU		UK		FR		Spain		The Netherlands		PT		HU		CH		FN		ES	
	D	Ranking	D	Ranking	D	Ranking	D	Ranking	D	Ranking	D	Ranking	D	Ranking	D	Ranking	D	Ranking	D	Ranking
Whole grains	0.08	5	0.11	4	0.06	6	0.04	9	0.13	2	0.05	7	0.18	2	0.09	5	0.15	2	0.12	3
Tubers	0.00	15	0.00	15	0.01	13	0.00	15	0.00	15	0.01	13	0.00	14	0.00	15	0.00	15	0.00	14
Vegetables	0.20	1	0.20	1	0.28	1	0.18	2	0.11	4	0.19	1	0.18	1	0.20	1	0.13	3	0.21	1
Fruits	0.18	2	0.17	2	0.16	2	0.13	3	0.16	1	0.17	2	0.14	4	0.18	2	0.16	1	0.19	2
Legumes	0.12	4	0.17	3	0.09	4	0.09	5	0.09	5	0.06	6	0.15	3	0.10	4	0.08	5	0.04	8
Beef and lamb	0.01	13	0.03	10	0.00	15	0.00	13	0.01	13	0.01	12	0.00	15	0.02	13	0.02	14	0.00	15
Pork	0.01	12	0.04	7	0.00	14	0.01	12	0.02	12	0.02	11	0.00	13	0.04	12	0.03	11	0.01	12
Poultry	0.07	6	0.10	5	0.08	5	0.05	8	0.05	9	0.05	8	0.02	9	0.06	6	0.05	8	0.06	7
Eggs	0.04	9	0.02	11	0.05	7	0.07	7	0.07	7	0.03	9	0.07	6	0.04	11	0.07	6	0.09	6
Fish and seafood	0.02	10	0.02	13	0.02	11	0.01	11	0.03	11	0.02	10	0.02	10	0.04	10	0.03	10	0.02	11
Nuts	0.02	11	0.05	6	0.04	10	0.00	14	0.05	10	0.01	14	0.02	11	0.05	8	0.03	12	0.03	10
Added sugars	0.06	8	0.03	9	0.04	9	0.12	4	0.08	6	0.17	3	0.11	5	0.04	9	0.07	7	0.09	5
Unsaturated oils	0.13	3	0.02	12	0.14	3	0.19	1	0.06	8	0.09	5	0.04	8	0.11	3	0.11	4	0.03	9
Saturated Fats	0.07	7	0.04	8	0.04	8	0.09	6	0.11	3	0.14	4	0.05	7	0.05	7	0.04	9	0.10	4
Dairy	0.00	14	0.00	14	0.01	12	0.02	10	0.01	14	0.00	15	0.01	12	0.00	14	0.02	13	0.00	13

Note: D = Standardized dominance.

Table S12. Dominance analysis of food group consumption (g/day) on the ELI index.

Food group	EU		UK		FR		Spain		The Netherlands		PT		HU		CH		FN		ES	
	D	Ranking	D	Ranking	D	Ranking	D	Ranking	D	Ranking	D	Ranking	D	Ranking	D	Ranking	D	Ranking	D	Ranking
Whole grains	0.06	8	0.08	6	0.05	8	0.04	11	0.09	6	0.05	7	0.11	4	0.06	7	0.08	6	0.09	5
Tubers	0.05	10	0.04	10	0.05	7	0.06	8	0.05	11	0.02	13	0.09	6	0.04	11	0.04	11	0.09	4
Vegetables	0.13	1	0.13	2	0.16	1	0.16	1	0.10	2	0.15	2	0.14	1	0.13	2	0.11	3	0.14	3
Fruits	0.12	3	0.10	3	0.12	3	0.11	3	0.12	1	0.15	1	0.12	3	0.13	1	0.12	2	0.15	2
Legumes	0.10	5	0.14	1	0.08	5	0.06	7	0.07	7	0.05	8	0.12	2	0.09	5	0.08	5	0.03	12
Beef and lamb	0.06	6	0.07	8	0.04	12	0.05	9	0.07	8	0.06	6	0.06	9	0.05	8	0.09	4	0.05	7
Pork	0.04	12	0.07	9	0.04	11	0.04	10	0.09	4	0.05	9	0.00	15	0.06	6	0.06	8	0.03	14
Poultry	0.05	9	0.08	7	0.03	14	0.03	12	0.02	13	0.04	10	0.03	11	0.03	14	0.03	13	0.03	13
Eggs	0.03	14	0.01	15	0.04	9	0.08	5	0.05	12	0.03	11	0.06	7	0.03	13	0.05	10	0.04	9
Fish and seafood	0.13	2	0.09	4	0.15	2	0.14	2	0.09	5	0.15	3	0.09	5	0.13	3	0.13	1	0.17	1
Nuts	0.03	13	0.09	5	0.04	10	0.01	15	0.10	3	0.01	15	0.04	10	0.05	9	0.04	12	0.05	8
Added sugars	0.06	7	0.04	11	0.05	6	0.07	6	0.06	9	0.10	4	0.06	8	0.05	10	0.07	7	0.06	6
Unsaturated oils	0.10	4	0.03	12	0.10	4	0.10	4	0.05	10	0.09	5	0.02	12	0.12	4	0.06	9	0.03	11
Saturated Fats	0.04	11	0.03	13	0.04	13	0.02	14	0.02	14	0.03	12	0.01	14	0.03	12	0.02	14	0.03	10
Dairy	0.01	15	0.01	14	0.00	15	0.02	13	0.02	15	0.02	14	0.02	13	0.01	15	0.02	15	0.02	15

Note: D = Standardized dominance.

Table S13. Dominance analysis of food group consumption (g/day) on the EDL-I index.

Food group	EU		UK		FR		Spain		The Netherlands		PT		HU		CH		FN		ES	
	**D**	**Ranking**	**D**	**Ranking**	**D**	**Ranking**	**D**	**Ranking**	**D**	**Ranking**	**D**	**Ranking**	**D**	**Ranking**	**D**	**Ranking**	**D**	**Ranking**	**D**	**Ranking**
Whole grains	0.01	12	0.02	11	0.00	12	0.00	14	0.01	11	0.01	11	0.00	11	0.01	10	0.01	11	0.01	10
Tubers	0.02	10	0.02	12	0.02	8	0.02	10	0.05	9	0.03	9	0.04	7	0.02	9	0.04	8	0.07	4
Vegetables	0.06	7	0.07	8	0.05	6	0.05	8	0.08	7	0.06	7	0.05	6	0.05	6	0.05	7	0.04	8
Fruits	0.19	1	0.13	2	0.16	3	0.27	1	0.18	1	0.26	1	0.28	1	0.18	3	0.21	2	0.38	1
Legumes	0.01	14	0.02	10	0.00	15	0.01	13	0.01	12	0.00	15	0.00	13	0.00	15	0.01	13	0.00	15
Beef and lamb	0.13	4	0.17	1	0.12	4	0.13	2	0.11	5	0.20	2	0.02	10	0.08	5	0.07	5	0.06	5
Pork	0.09	5	0.08	7	0.06	5	0.12	3	0.13	3	0.16	3	0.14	3	0.08	4	0.16	3	0.14	3
Poultry	0.04	9	0.06	9	0.01	11	0.04	9	0.01	10	0.04	8	0.04	9	0.01	11	0.01	10	0.01	11
Eggs	0.07	6	0.10	4	0.04	7	0.06	7	0.06	8	0.06	6	0.07	5	0.04	7	0.06	6	0.05	6
Fish and seafood	0.01	13	0.00	14	0.00	13	0.01	11	0.00	14	0.01	12	0.00	15	0.00	13	0.01	12	0.00	12
Nuts	0.05	8	0.09	6	0.02	9	0.10	4	0.10	6	0.01	10	0.04	8	0.03	8	0.04	9	0.02	9
Added sugars	0.13	3	0.10	5	0.22	2	0.07	6	0.11	4	0.06	5	0.08	4	0.26	1	0.08	4	0.05	7
Unsaturated oils	0.01	11	0.01	13	0.01	10	0.01	12	0.00	15	0.00	14	0.00	12	0.01	12	0.00	14	0.00	14
Saturated Fats	0.18	2	0.13	3	0.28	1	0.10	5	0.14	2	0.08	4	0.23	2	0.22	2	0.25	1	0.16	2
Dairy	0.00	15	0.00	15	0.00	14	0.00	15	0.01	13	0.00	13	0.00	14	0.00	14	0.00	15	0.00	13

Note: D = Standardized dominance.

Table S14. Multivariate regression analyses of sociodemographic factors associated with WISH, ELI, and ELD-I in the United Kingdom.

**United Kingdom**	**WISH**							**ELI**							**ELD-I**						
	**Coef**	**95%IC**		**p**	**η2**	**95%IC**		**Coef**	**95%CI**		**p**	**η2**	**95%IC**		**Coef**	**95%IC**		**p**	**η2**	**95%IC**	
Age																					
[45:65[	10.36	5.64	15.07	0.000	0.05	0.02	0.08	2.23	0.71	3.76	0.004	0.03	0.01	0.06	11.65	0.62	22.67	0.038	0.01	0.00	0.04
[65:80[	11.27	5.51	17.03	0.000				2.18	0.32	4.03	0.022				6.12	-7.34	19.57	0.372			
Sex																					
Men	-0.37	-4.74	4.01	0.869	0.02	0.00	0.04	-1.26	-2.67	0.15	0.080	0.02	0.00	0.05	-7.50	-17.72	2.71	0.150	0.01	0.00	0.04
Age#Sex																					
[45:65[#Men	-5.44	-12.13	1.26	0.111				-0.50	-2.66	1.66	0.651				-2.77	-18.41	12.87	0.728			
[65:80[#Men	-7.08	-15.36	1.20	0.093				-1.12	-3.79	1.55	0.411				-4.82	-24.16	14.53	0.625			

Note. Values are regression coefficients (Coef) from multivariate linear regression models with WISH, ELI, and ELD-I as dependent variables. Models are adjusted for age, sex, and their interaction (Age×Sex). 95% CI indicates 95% confidence interval; p values are two-sided. η^2 represents partial eta-squared, indicating effect size. Reference categories are age 18–44 years and women.

Table S15. Multivariate regression analyses of sociodemographic factors associated with WISH, ELI, and ELD-I in France.

France	WISH							ELI							ELD-I						
	**Coef**	**95%IC**		**p**	**η2**	**95%IC**		**Coef**	**95%CI**		**p**	**η2**	**95%IC**		**Coef**	**95%IC**		**p**	**η2**	**95%IC**	
Age																					
[45:65[	10.02	7.40	12.64	0.000	0.10	0.08	0.12	2.48	1.72	3.24	0.000	0.07	0.05	0.09	20.18	12.82	27.54	0.000	0.06	0.04	0.08
[65:80[	13.62	10.68	16.56	0.000				3.56	2.71	4.42	0.000				33.94	25.67	42.20	0.000			
Sex																					
Men	-3.05	-5.63	-0.48	0.020	0.01	0.00	0.02	-0.56	-1.31	0.19	0.141	0.01	0.00	0.02	3.58	-3.65	10.81	0.332	0.00	0.00	0.01
Education																					
Higher education	6.12	3.60	8.63	0.000	0.01	0.00	0.02	2.39	1.66	3.12	0.000	0.02	0.01	0.03	18.36	11.28	25.43	0.000	0.02	0.01	0.03
Age#Sex																					
[45:65[#Men	0.33	-3.32	3.99	0.858				0.10	-0.97	1.16	0.856				-2.28	-12.57	8.00	0.663			
[65:80[#Men	-0.01	-4.26	4.24	0.997				-0.39	-1.62	0.85	0.538				-10.04	-21.99	1.90	0.099			
Age#Education																					
[45:65[#Higher education	-4.27	-8.08	-0.47	0.028				-0.96	-2.06	0.15	0.090				-2.13	-12.82	8.57	0.697			
[65:80[#Higher education	-2.83	-8.04	2.38	0.287				-1.35	-2.87	0.16	0.080				-4.57	-19.20	10.07	0.541			
Sex#Education																					
Men#Higher education	-0.53	-4.09	3.04	0.772				-0.72	-1.76	0.31	0.170				-6.74	-16.75	3.28	0.187			
Age#Sex#Education																					
[45:65[#Men#Higher Education	-1.40	-6.84	4.03	0.612				-0.27	-1.85	1.31	0.739				-1.99	-17.26	13.28	0.798			
[65:80[#Men#Higher education	1.17	-6.30	8.64	0.759				0.86	-1.31	3.04	0.435				0.50	-20.49	21.49	0.962			

Note. Values are regression coefficients (Coef) from multivariate linear regression models with WISH, ELI, and ELD-I as dependent variables. Models are adjusted for age, sex, education and their interaction (Age×Sex×Education). 95% CI indicates 95% confidence interval; p values are two-sided. η^2 represents partial eta-squared, indicating effect size. Reference categories are age 18–44 years, women, and lower education level.

Table S16. Multivariate regression analyses of sociodemographic factors associated with WISH, ELI, and ELD-I in Spain.

Spain	WISH							ELI							ELD-I						
	**Coef**	**95%IC**		**p**	**η2**	**95%IC**		**Coef**	**95%CI**		**p**	**η2**	**95%IC**		**Coef**	**95%IC**		**p**	**η2**	**95%IC**	
Age																					
[45:65[	9.11	4.87	13.34	0.000	0.09	0.06	0.13	2.69	1.54	3.85	0.000	0.08	0.05	0.12	24.00	14.62	33.39	0.000	0.09	0.06	0.12
[65:80[	11.53	6.57	16.49	0.000				3.37	2.02	4.72	0.000				38.94	27.95	49.94	0.000			
Sex																					
Men	-0.62	-4.85	3.60	0.773	0.00	0.00	0.01	-0.65	-1.81	0.50	0.266	0.01	0.00	0.02	-1.51	-10.88	7.87	0.753	0.01	0.00	0.02
Education																					
Higher education	1.68	-2.54	5.90	0.435	0.00	0.00	0.02	0.52	-0.64	1.67	0.379	0.01	0.00	0.02	8.86	-0.50	18.22	0.063	0.00	0.00	0.01
Age#Sex																					
[45:65[#Men	2.32	-3.75	8.40	0.453				-0.21	-1.87	1.45	0.807				-8.81	-22.29	4.67	0.200			
[65:80[#Men	-0.07	-7.52	7.39	0.986				-0.54	-2.57	1.50	0.604				-16.32	-32.86	0.22	0.053			
Age#Education																					
[45:65[#Higher education	0.64	-6.23	7.51	0.855				0.43	-1.45	2.30	0.654				-12.22	-27.45	3.01	0.116			
[65:80[#Higher education	5.79	-3.81	15.40	0.237				0.27	-2.35	2.89	0.838				-14.60	-35.90	6.71	0.179			
Sex#Education																					
Men#Higher education	0.57	-5.34	6.48	0.850				0.50	-1.12	2.11	0.547				-10.64	-23.75	2.48	0.112			
Age#Sex#Education																					
[45:65[#Men#Higher Education	-4.62	-14.09	4.85	0.339				-1.63	-4.22	0.95	0.216				13.92	-7.09	34.94	0.194			
[65:80[#Men#Higher education	-5.65	-18.73	7.42	0.396				0.45	-3.12	4.02	0.804				31.51	2.51	60.51	0.033			

Note. Values are regression coefficients (Coef) from multivariate linear regression models with WISH, ELI, and ELD-I as dependent variables. Models are adjusted for age, sex, education and their interaction (Age×Sex×Education). 95% CI indicates 95% confidence interval; p values are two-sided. η^2 represents partial eta-squared, indicating effect size. Reference categories are age 18–44 years, women, and lower education level.

Table S17. Multivariate regression analyses of sociodemographic factors associated with WISH, ELI, and ELD-I in the Netherlands.

The Netherlands	WISH							ELI							ELD-I						
	Coef	95%IC		p	η2	95%IC		Coef	95%CI		p	η2	95%IC		Coef	95%IC		p	η2	95%IC	
Age																					
[45:65[	3.50	0.04	6.96	0.048	0.01	0.00	0.02	0.11	-0.84	1.06	0.815	0.00	0.00	0.01	0.06	-7.15	7.27	0.987	0.00	0.00	0.00
[65:80[	6.15	2.20	10.10	0.002				1.00	-0.09	2.08	0.071				8.18	-0.04	16.40	0.051			
Sex																					
Men	1.16	-2.39	4.71	0.522	0.01	0.00	0.01	-0.77	-1.74	0.21	0.122	0.02	0.01	0.03	-3.13	-10.52	4.26	0.406	0.02	0.01	0.03
Education																					
Higher education	11.75	8.37	15.13	0.000	0.03	0.02	0.05	3.01	2.09	3.94	0.000	0.05	0.03	0.07	19.10	12.07	26.14	0.000	0.04	0.03	0.06
Age#Sex																					
[45:65[#Men	-0.58	-5.52	4.37	0.818				0.42	-0.94	1.78	0.544				0.12	-10.18	10.43	0.981			
[65:80[#Men	-2.03	-7.85	3.79	0.494				0.18	-1.42	1.78	0.825				-10.55	-22.68	1.58	0.088			
Age#Education																					
[45:65[#Higher education	-3.22	-8.22	1.79	0.208				0.01	-1.36	1.39	0.985				1.68	-8.75	12.11	0.752			
[65:80[#Higher education	-2.49	-8.81	3.82	0.439				-0.28	-2.01	1.45	0.752				-0.67	-13.82	12.48	0.920			
Sex#Education																					
Men#Higher education	-5.59	-10.35	-0.82	0.022				-1.08	-2.39	0.23	0.105				-5.67	-15.60	4.26	0.263			
Age#Sex#Education																					
[45:65[#Men#Higher Education	-0.45	-7.50	6.61	0.901				-0.31	-2.25	1.62	0.752				-3.65	-18.35	11.05	0.626			
[65:80[#Men#Higher education	-0.74	-9.60	8.11	0.869				-0.61	-3.04	1.82	0.625				0.66	-17.78	19.11	0.944			

Note. Values are regression coefficients (Coef) from multivariate linear regression models with WISH, ELI, and ELD-I as dependent variables. Models are adjusted for age, sex, education and their interaction (Age×Sex×Education). 95% CI indicates 95% confidence interval; p values are two-sided. η^2 represents partial eta-squared, indicating effect size. Reference categories are age 18–44 years, women, and lower education level.

Table S18. Multivariate regression analyses of sociodemographic factors associated with WISH, ELI, and ELD-I in Portugal.

Portugal	WISH							ELI							ELD-I						
	Coef	95%IC		p	η2	95%IC		Coef	95%CI		p	η2	95%IC		Coef	95%IC		p	η2	95%IC	
Age																					
[45:65[	9.91	8.01	11.81	0.000	0.04	0.03	0.06	2.39	1.87	2.91	0.000	0.04	0.03	0.05	15.76	11.05	20.47	0.000	0.05	0.03	0.06
[65:80[	14.37	12.23	16.51	0.000				3.37	2.79	3.96	0.000				34.38	29.07	39.69	0.000			
Sex																					
Men	-0.28	-2.17	1.62	0.774	0.01	0.00	0.01	-0.39	-0.90	0.13	0.144	0.01	0.00	0.01	-11.40	-16.10	-6.70	0.000	0.02	0.01	0.03
Education																					
Higher education	4.65	2.42	6.87	0.000	0.00	0.00	0.00	1.56	0.95	2.17	0.000	0.00	0.00	0.01	12.72	7.21	18.23	0.000	0.00	0.00	0.01
Age#Sex																					
[45:65[#Men	-2.51	-5.18	0.17	0.066				-0.53	-1.26	0.20	0.158				3.46	-3.16	10.08	0.305			
[65:80[#Men	-1.52	-4.63	1.59	0.339				0.10	-0.75	0.95	0.815				0.08	-7.62	7.79	0.983			
Age#Education																					
[45:65[#Higher education	-3.93	-7.68	-0.18	0.040				-0.65	-1.67	0.38	0.217				1.60	-7.70	10.90	0.736			
[65:80[#Higher education	-1.21	-7.41	4.98	0.701				-0.42	-2.12	1.27	0.625				3.83	-11.51	19.18	0.624			
Sex#Education																					
Men#Higher education	-4.42	-7.74	-1.11	0.010				-0.94	-1.84	-0.03	0.043				-7.78	-15.99	0.42	0.063			
Age#Sex#Education																					
[45:65[#Men#Higher Education	1.43	-4.19	7.06	0.617				0.04	-1.50	1.58	0.957				-11.61	-25.55	2.33	0.103			
[65:80[#Men#Higher education	-1.86	-11.10	7.38	0.694				-0.95	-3.47	1.58	0.463				-5.66	-28.55	17.24	0.628			

Note. Values are regression coefficients (Coef) from multivariate linear regression models with WISH, ELI, and ELD-I as dependent variables. Models are adjusted for age, sex, education and their interaction (Age×Sex×Education). 95% CI indicates 95% confidence interval; p values are two-sided. η^2 represents partial eta-squared, indicating effect size. Reference categories are age 18–44 years, women, and lower education level.

Table S19. Multivariate regression analyses of sociodemographic factors associated with WISH, ELI, and ELD-I in Hungary.

Hungary	WISH							ELI							ELD-I						
	Coef	95%IC		p	η2	95%IC		Coef	95%CI		p	η2	95%IC		Coef	95%IC		p	η2	95%IC	
Age																					
[45:65[	3.60	-0.54	7.74	0.088	0.05	0.02	0.08	1.60	0.44	2.76	0.007	0.05	0.02	0.09	5.80	-2.89	14.48	0.191	0.04	0.02	0.07
[65:80[	8.13	3.12	13.14	0.001				2.20	0.80	3.60	0.002				18.68	8.17	29.20	0.001			
Sex																					
Men	-4.38	-8.52	-0.24	0.038	0.00	0.00	0.02	-0.95	-2.11	0.20	0.106	0.00	0.00	0.02	-9.85	-18.54	-1.15	0.026	0.00	0.00	0.02
Education																					
Higher education	0.25	-3.93	4.44	0.905	0.00	0.00	0.02	0.78	-0.39	1.95	0.191	0.00	0.00	0.01	3.60	-5.18	12.38	0.421	0.00	0.00	0.02
Age#Sex																					
[45:65[#Men	3.01	-2.98	9.01	0.324				0.47	-1.21	2.15	0.582				11.53	-1.06	24.12	0.073			
[65:80[#Men	0.72	-7.48	8.91	0.864				0.67	-1.63	2.96	0.568				-2.30	-19.51	14.90	0.793			
Age#Education																					
[45:65[#Higher education	4.07	-3.15	11.30	0.269				0.34	-1.68	2.36	0.743				5.45	-9.72	20.61	0.481			
[65:80[#Higher education	-1.37	-12.41	9.67	0.807				-1.22	-4.31	1.87	0.438				-7.17	-30.35	16.01	0.544			
Sex#Education																					
Men#Higher education	2.49	-3.65	8.63	0.426				-0.52	-2.24	1.19	0.549				2.49	-10.41	15.39	0.705			
Age#Sex#Education																					
[45:65[#Men#Higher Education	-3.76	-13.89	6.37	0.466				0.20	-2.63	3.03	0.890				-4.42	-25.69	16.84	0.683			
[65:80[#Men#Higher education	3.27	-12.65	19.19	0.687				1.26	-3.19	5.72	0.578				8.58	-24.85	42.01	0.614			

Note. Values are regression coefficients (Coef) from multivariate linear regression models with WISH, ELI, and ELD-I as dependent variables. Models are adjusted for age, sex, education and their interaction (Age×Sex×Education). 95% CI indicates 95% confidence interval; p values are two-sided. η^2 represents partial eta-squared, indicating effect size. Reference categories are age 18–44 years, women, and lower education level.

Table S20. Multivariate regression analyses of sociodemographic factors associated with WISH, ELI, and ELD-I in Switzerland.

Switzerland	WISH							ELI							ELD-I						
	Coef	95%IC		p	η^2	95%IC		Coef	95%CI		p	η^2	95%IC		Coef	95%IC		p	η^2	95%IC	
Age																					
[45:65[	5.17	2.16	8.18	0.001	0.02	0.01	0.03	0.97	0.08	1.86	0.032	0.01	0.00	0.02	1.73	-6.37	9.82	0.676	0.00	0.00	0.01
[65:80[	4.34	0.80	7.88	0.016				0.64	-0.40	1.69	0.226				0.23	-9.28	9.75	0.962			
Sex																					
Men	-6.42	-9.47	-3.37	0.000	0.03	0.01	0.04	-2.44	-3.34	-1.54	0.000	0.03	0.02	0.05	-15.26	-23.45	-7.06	0.000	0.02	0.01	0.03
Education																					
Higher education	1.73	-1.25	4.71	0.254	0.00	0.00	0.01	0.84	-0.04	1.71	0.061	0.00	0.00	0.01	3.87	-4.13	11.87	0.343	0.01	0.00	0.02
Age#Sex																					
[45:65[#Men	-0.84	-5.34	3.66	0.714				0.45	-0.87	1.78	0.503				5.54	-6.55	17.63	0.369			
[65:80[#Men	1.60	-4.00	7.20	0.575				1.01	-0.64	2.65	0.232				4.01	-11.03	19.06	0.601			
Age#Education																					
[45:65[#Higher education	-2.65	-7.20	1.90	0.253				-0.30	-1.64	1.04	0.656				9.75	-2.47	21.98	0.118			
[65:80[#Higher education	0.79	-5.25	6.83	0.798				0.21	-1.57	1.98	0.821				7.85	-8.38	24.07	0.343			
Sex#Education																					
Men#Higher education	-0.25	-4.43	3.94	0.908				0.09	-1.15	1.32	0.888				3.66	-7.60	14.92	0.524			
Age#Sex#Education																					
[45:65[#Men#Higher Education	2.11	-4.27	8.48	0.517				-0.13	-2.01	1.74	0.889				-11.94	-29.07	5.20	0.172			
[65:80[#Men#Higher education	-1.02	-9.43	7.39	0.812				-0.46	-2.94	2.02	0.717				-3.03	-25.64	19.58	0.793			

Note. Values are regression coefficients (Coef) from multivariate linear regression models with WISH, ELI, and ELD-I as dependent variables. Models are adjusted for age, sex, education and their interaction (Age×Sex×Education). 95% CI indicates 95% confidence interval; p values are two-sided. η^2 represents partial eta-squared, indicating effect size. Reference categories are age 18–44 years, women, and lower education level.

Table S21. Multivariate regression analyses of sociodemographic factors associated with WISH, ELI, and ELD-I in Finland.

Finland	WISH							ELI							ELD-I						
	Coef	95%IC		p	η2	95%IC		Coef	95%CI		p	η2	95%IC		Coef	95%IC		p	η2	95%IC	
Age																					
[45:65[	4.06	1.37	6.75	0.003	0.02	0.01	0.04	0.70	-0.09	1.49	0.082	0.02	0.01	0.03	5.34	-1.50	12.19	0.126	0.00	0.00	0.01
[65:80[	5.55	2.46	8.63	0.000				1.42	0.51	2.33	0.002				7.34	-0.52	15.20	0.067			
Sex																					
Men	-4.30	-6.81	-1.78	0.001	0.01	0.00	0.03	-1.70	-2.44	-0.96	0.000	0.02	0.01	0.04	-15.88	-22.29	-9.48	0.000	0.04	0.03	0.07
Age#Sex																					
[45:65[#Men	-0.63	-4.42	3.15	0.743				0.12	-0.99	1.24	0.828				-6.26	-15.89	3.37	0.203			
[65:80[#Men	1.12	-3.33	5.57	0.622				0.42	-0.90	1.73	0.533				-2.55	-13.89	8.79	0.659			

Note. Values are regression coefficients (Coef) from multivariate linear regression models with WISH, ELI, and ELD-I as dependent variables. Models are adjusted for age, sex, and their interaction (Age×Sex). 95% CI indicates 95% confidence interval; p values are two-sided. η^2 represents partial eta-squared, indicating effect size. Reference categories are age 18–44 years and women.

Table S22. Multivariate regression analyses of sociodemographic factors associated with WISH, ELI, and ELD-I in Estonia.

Estonia	WISH							ELI							ELD-I						
	Coef	95%IC		p	η2	95%IC		Coef	95%CI		p	η2	95%IC		Coef	95%IC		p	η2	95%IC	
Age																					
[45:65[	3.32	0.47	6.17	0.023	0.02	0.01	0.03	0.54	-0.31	1.39	0.211	0.02	0.01	0.03	1.72	-8.62	12.06	0.744	0.00	0.00	0.01
[65:80[	5.90	2.96	8.84	0.000				1.19	0.31	2.06	0.008				6.02	-4.65	16.69	0.269			
Sex																					
Men	-2.26	-4.62	0.11	0.062	0.01	0.01	0.02	-1.77	-2.47	-1.06	0.000	0.02	0.01	0.04	-18.95	-27.54	-10.36	0.000	0.05	0.03	0.06
Education																					
Higher education	3.27	0.96	5.58	0.006	0.00	0.00	0.01	0.92	0.24	1.61	0.009	0.01	0.00	0.02	11.46	3.08	19.85	0.007	0.01	0.00	0.02
Age#Sex																					
[45:65[#Men	0.07	-3.74	3.88	0.972				0.95	-0.19	2.08	0.102				-0.54	-14.35	13.27	0.939			
[65:80[#Men	-2.11	-6.53	2.31	0.350				0.11	-1.21	1.43	0.870				-2.78	-18.81	13.25	0.734			
Age#Education																					
[45:65[#Higher education	-1.52	-5.05	2.01	0.399				0.35	-0.70	1.40	0.516				3.74	-9.06	16.54	0.567			
[65:80[#Higher education	-0.75	-4.72	3.23	0.712				-0.14	-1.32	1.04	0.815				9.99	-4.42	24.39	0.174			
Sex#Education																					
Men#Higher education	-1.68	-4.86	1.50	0.299				0.21	-0.74	1.15	0.670				-3.05	-14.58	8.48	0.604			
Age#Sex#Education																					
[45:65[#Men#Higher Education	1.08	-3.88	6.04	0.669				-0.76	-2.24	0.72	0.314				0.32	-17.67	18.31	0.972			
[65:80[#Men#Higher education	0.20	-6.02	6.43	0.949				-0.20	-2.05	1.65	0.832				-18.93	-41.50	3.64	0.100			

Note. Values are regression coefficients (Coef) from multivariate linear regression models with WISH, ELI, and ELD-I as dependent variables. Models are adjusted for age, sex, education and their interaction (Age×Sex×Education). 95% CI indicates 95% confidence interval; p values are two-sided. η^2 represents partial eta-squared, indicating effect size. Reference categories are age 18–44 years, women, and lower education level.

Extended discussion: Heterogeneity in European food consumption

Marked heterogeneity in food consumption patterns across countries was found in the current study. For example, our findings reflect those of Price et al., who reported whole grain intakes ranging from 4.7 g/d in France to 63 g/d in Finland [1]. The high consumption in Finland is attributable to traditional foods such as rye bread and oatmeal, which are linked to improved health outcomes [2]. In contrast, nearly half of the French adults reported zero whole grain intake in previous research [3]. Regular whole grain intake is associated with improved health outcomes [4], supporting the value of interventions such as the French national promotion campaign of whole grains and legumes, which has shown particular benefits among lower socioeconomic groups [5].

Legume consumption was low in all countries, consistent with Hughes et al., who reported that no European country met the legume target, with more than one-third of countries reporting median intakes of less than 10 g/d [6]. Spain and the UK showed the highest legume intakes, likely reflecting the traditional dietary patterns [7]. Nuts were the least consumed food group, with a mean intake of 5.2 g/d, however the Netherlands reported more than double this average. These findings are consistent with those of the EPIC cohort [8] and recent data from Daas et al. [9], underscoring the importance of promoting nut intake, given its strong association with reduced risks of cardiovascular disease, cancer, and all-cause mortality [10].

Spain had the highest consumption of unsaturated oils and the lowest levels of saturated fats, whereas the UK and Switzerland exhibited low consumption of unsaturated oils and high levels of saturated fats. These patterns reflect regional differences, with Mediterranean countries favoring vegetable oils and Northern/Central Europe relying more on animal fats [11]. Replacing saturated fats with polyunsaturated fats reduces cardiovascular risk [12], and the environmental benefits of plant-based oils support the promotion of oilseed crops in dietary policies, considering ecological and economic factors [13].

Although fruit and vegetable consumption exceeded the global intake [14], they remained below the PHD targets [15]. The European vegetable intake (189.6 g/d) covered only 63% of the 300 g/d benchmark, and fruit intake (177.1 g/d) was also insufficient. The UK had the largest deficits, with previous estimates showing only 88 g of fruit and 140 g of vegetables consumed daily [16]. In Spain, low vegetable intake contrasted with

relatively adequate fruit consumption, reflecting patterns observed in the ENRICA cohort, where only 2.75% of adults met the recommendation of three daily servings of vegetables [17].

Spain and Portugal rank among the highest in Europe for fish consumption, showing substantial adherence to the PHD recommendations [18]. In contrast, Hungary and the Netherlands have the lowest intakes, below the PHD benchmark. Fish consumption is especially low in Central and Eastern Europe, less than half the European average, and lowest in Hungary, where only 20% of adults eat fish monthly [19]. Low intake in the Netherlands reflects traditional diets favoring meat and potatoes [20], while limited access to the sea also contributes to the low intake in Central and Eastern Europe [19].

Overconsumption of foods to limit was common across countries. The average red meat intake was 82.5 g/d—nearly six times the recommended 14 g/d—with Estonia and Hungary having particularly high intakes. In Estonia, pork alone exceeded the PHD threshold by over 1,100%, reflecting the cultural preferences and agricultural policies. Traditional diets in both countries emphasize red meat, potatoes, refined grains, and processed fats [21,22]. Reducing red meat consumption remains challenging due to entrenched cultural, economic, and political factors, including subsidies that lower meat prices relative to plant-based foods [23,24]. In 2020, European per capita meat consumption was more than double the global average and is expected to remain stable until 2030 [25]. Addressing this requires integrated policies that align public health, environmental, and agricultural goals, such as the Farm-to-Fork strategy, although fragmented national dietary guidelines hinder implementation [26].

Intake of added sugars (54.6 g/d) and saturated fats (28.2 g/d) exceeded the PHD targets across all countries. Switzerland and the UK showed particularly high added sugar intake, consistent with reports of total and added sugars two to three times above the recommended limits [27,28]. In the UK, soft drinks account for roughly one-third of sugar intake, compared to 12% in Portugal [29]. Conversely, in Switzerland, sweets and pastries are the main sources of sugar [30]. Nearly 90% of Portuguese adults meet the WHO guideline of <10% energy from added sugars, explaining their closer alignment with the PHD targets [29]. Despite regulatory efforts, interventions such as taxation, front-of-pack labeling, and marketing restrictions remain inconsistently implemented across Europe, highlighting persistent systemic challenges and the need for stronger public health policies [31-33].

Transforming Europe's food systems toward healthier and more sustainable diets requires coordinated cultural, structural, and behavioral changes, supported by multi-level and multi-sectoral governance with policy playing a central role. Coordinated action across all levels and sectors remains essential to overcome entrenched power imbalances and achieve meaningful change. Table S4 summarizes examples of actions that can support this transformation.

Table S23. Example multi-level and -sectoral actions that can promote healthier and more sustainable food choice architecture

	**Example Multi-Sectoral Actions**
**Micro-Level** (community)	Utilising participatory governance approaches where communities can actively shape their food environments based on principles of food sovereignty.<br>- Supporting Local Solidarity-based Partnerships for Agroecology and Community Supported Agriculture [34]<br>- Territorial markets [35]
**Meso-level** (regional/national government)	Public bodies at a regional level as well as Member States can implement context-sensitive initiatives and policies to support local, healthier and more sustainable food systems.<br>- Disincentivising farming practices that deliver agroecosystem disservices, such as the use of chemical pesticides<br>- Incentivising farmers to adopt environmentally friendly practices that deliver agri-environment-climate public goods [36], including through the use of innovative land tenure agreements [37]<br>- Implementing innovative/dynamic public procurement approaches in public facilities such as providing healthier and more sustainable school meals [38]<br>- Designing zoning laws and providing governments the legal tools to create healthier food environments, such as the 'No-Fry Zone' policy at a local level in Ireland [103]<br>- Policies to protect vulnerable population groups from the harmful impact of food marketing [39]<br>- Removing VAT on fruits and vegetables [40]

**Macro-level** (EU)	Promote fairness and transparency in policy-making processes that ensures a level playing field for all key food system stakeholders to prevent corporate/regulatory capture [41].<br>- Disincentivising and preventing land grabbing [42]<br>- Phasing out incentives for red meat and dairy production<br>- Phasing out and disincentivizing farming practices that deliver agroecosystem disservices, such as the use of chemical pesticides in agriculture [43]<br>- Incentivisation of farming practices that deliver agri-environment-climate public goods [36,37]<br>- Universal free school meals that are healthy/sustainable<br>- Regulations against harmful food products, especially ultraprocessed foods (analogous to regulations for trans fats) [44]

Supplementary References

1. Price EJ, Barrett EM, Batterham MJ, Beck EJ (2024) Exploring the reporting, intake and recommendations of primary food sources of whole grains globally: a scoping review. Br J Nutr. https://doi.org/10.1017/S0007114524002678
2. Tammi R, Männistö S, Maukonen M, Kaartinen NE (2024) Whole grain intake, diet quality and risk factors of chronic diseases: results from a population-based study in Finnish adults. Eur J Nutr. https://doi.org/10.1007/s00394-023-03272-z
3. Bellisle F, Hébel P, Colin J, Reyé B, Hopkins S (2014) Consumption of whole grains in French children, adolescents and adults. Br J Nutr. https://doi.org/10.1017/S0007114514002670
4. Langmann F et al (2025) Plant-based diets, legumes, and prevalence of cardiometabolic risk factors in the NutriNet-Santé cohort. Eur J Nutr. https://doi.org/10.1007/s00394-025-03722-w
5. Fassier P, Rabès A, Ducrot P, Serry AJ (2023) Impact of a French social marketing campaign promoting pulse and whole grain consumption: results from a longitudinal cohort study. Front Nutr. https://doi.org/10.3389/fnut.2023.1208824

6. Hughes J, Pearson E, Grafenauer S (2022) Legumes-A Comprehensive Exploration of Global Food-Based Dietary Guidelines and Consumption. Nutrients. https://doi.org/10.3390/nu14153080
7. Henn K, Goddyn H, Olsen SB, Bredie WL (2022) Identifying behavioral and attitudinal barriers and drivers to promote consumption of pulses: A quantitative survey across five European countries. Food Qual Prefer. https://doi.org/10.1016/j.foodqual.2021.104455
8. Jenab M et al (2006) Consumption and portion sizes of tree nuts, peanuts and seeds in the European Prospective Investigation into Cancer and Nutrition (EPIC) cohorts from 10 European countries. Br J Nutr. https://doi.org/10.1017/bjn20061859
9. Daas MC, van 't Veer P, Temme EHM, Kuijsten A, Gurinović M, Biesbroek S (2025) Diversity of dietary protein patterns across Europe - Impact on nutritional quality and environmental sustainability. Curr Res Food Sci. https://doi.org/10.1016/j.crfs.2025.101019
10. Balakrishna R, Bjørnerud T, Bemanian M, Aune D, Fadnes LT (2022) Consumption of Nuts and Seeds and Health Outcomes Including Cardiovascular Disease, Diabetes and Metabolic Disease, Cancer, and Mortality: An Umbrella Review. Adv Nutr. https://doi.org/10.1093/advances/nmac077
11. Bajželj B, Laguzzi F, Röös E (2021) The role of fats in the transition to sustainable diets. Lancet Planet Health. https://doi.org/10.1016/S2542-5196(21)00194-7
12. Maki KC, Dicklin MR, Kirkpatrick CF (2021) Saturated fats and cardiovascular health: Current evidence and controversies. J Clin Lipidol. https://doi.org/10.1016/j.jacl.2021.09.049
13. Meijaard E, Abrams JF, Slavin JL, Sheil D (2022) Dietary Fats, Human Nutrition and the Environment: Balance and Sustainability. Front Nutr. https://doi.org/10.3389/fnut.2022.878644
14. Micha R, Khatibzadeh S, Shi P, Andrews KG, Engell RE, Mozaffarian D (2015) Global, regional and national consumption of major food groups in 1990 and 2010: a systematic analysis including 266 country-specific nutrition surveys worldwide. BMJ Open. https://doi.org/10.1136/bmjopen-2015-008705
15. Willett W et al (2019) Food in the Anthropocene: the EAT–Lancet Commission on healthy diets from sustainable food systems. Lancet. https://doi.org/10.1016/s0140-6736(18)31788-4

16. Eustachio Colombo P et al (2021) Pathways to "5-a-day": modeling the health impacts and environmental footprints of meeting the target for fruit and vegetable intake in the United Kingdom. Am J Clin Nutr. https://doi.org/10.1093/ajcn/nqab076
17. Vega-Cabello V et al (2025) Adherence to the healthy and sustainable dietary recommendations for the Spanish population and all-cause mortality. Rev Esp Cardiol. https://doi.org/10.1016/j.rec.2024.11.008
18. Miller V et al (2022) Global, regional, and national consumption of animal-source foods between 1990 and 2018: findings from the Global Dietary Database. Lancet Planet Health. https://doi.org/10.1016/S2542-5196(21)00352-1
19. Shivarov A (2023) Fish and Seafood Markets in Central and Eastern Europe. Izvestia J Union Scientists-Varna Econ Sci Series. https://doi.org/10.56065/IJUSV-ESS/2023.12.1.123
20. Waijers PM et al (2006) Dietary patterns and survival in older Dutch women. Am J Clin Nutr. https://doi.org/10.1093/ajcn/83.5.1170
21. Mõttus R, Realo A, Allik J, Deary IJ, Esko T, Metspalu A (2012) Personality traits and eating habits in a large sample of Estonians. Health Psychol. https://doi.org/10.1037/a0027041
22. Bárdos H et al (2022) Diet quality as assessed by Healthy Eating Index-2015 among Hungarian Roma living in settlements of Northeast Hungary. Sci Rep. https://doi.org/10.1038/s41598-022-23670-3
23. Rust NA et al (2020) How to transition to reduced-meat diets that benefit people and the planet. Sci Total Environ. https://doi.org/10.1016/j.scitotenv.2020.137208
24. Gu X, Bui LP, Wang F, Wang DD, Springmann M, Willett WC (2024) Global adherence to a healthy and sustainable diet and potential reduction in premature death. Proc Natl Acad Sci U S A. https://doi.org/10.1073/pnas.2319008121
25. OECD, FAO (2021) OECD-FAO Agricultural Outlook 2021–2030. OECD Publishing, Paris. https://doi.org/10.1787/19428846-en
26. Cué Rio M et al (2022) The elephant in the room is really a cow: using consumption corridors to define sustainable meat consumption in the European Union. Sustain Sci. https://doi.org/10.1007/s11625-022-01235-7

27. Watson F, Modi M (2018) Fresh start: A framework for healthy and sustainable diets in the UK-Situational analysis. UK Health Forum 2018. https://ukhealthforum.org.uk/wp-content/uploads/2019/01/UKHF_situational_analysis_FINAL.pdf
28. Linzmajer M, Eggenschwiler M, Bally L (2022) Der Schweizer Ernährungsatlas – Eine Schätzmethodik des Ernährungsverhaltens der Schweizer Bevölkerung basierend auf Einkaufsdaten. St.Gallen: Forschungszentrum für Handelsmanagement, Universität St.Gallen. https://www.alexandria.unisg.ch/server/api/core/bitstreams/b1adb76a-5d60-4755-bf70-ad214f19d4df/content
29. Marinho AR et al (2020) Total, added and free sugar intakes, dietary sources and determinants of consumption in Portugal: the National Food, Nutrition and Physical Activity Survey (IAN-AF 2015-2016). Public Health Nutr. https://doi.org/10.1017/S1368980019002519
30. Chatelan A, Gaillard P, Kruseman M, Keller A (2019) Total, Added, and Free Sugar Consumption and Adherence to Guidelines in Switzerland: Results from the First National Nutrition Survey menuCH. Nutrients. https://doi.org/10.3390/nu11051117
31. Bottari F, Mark-Herbert C (2022) Development of uniform food information -the case of front of package nutrition labels in the EU. Arch Public Health. https://doi.org/10.1186/s13690-022-00915-1
32. Leibinger A et al (2025) The impact of tiered soft drink taxes in Europe on mean sales-weighted sugar content of soft drinks: a quasi-experimental study. BMC Public Health. https://doi.org/10.1186/s12889-025-23331-w
33. Royo-Bordonada MÁ, Capellán LM, Junquera-Abaitua C, López JV, Gómez SF (2023) Spain facing the challenge of regulating unhealthy food advertising. Lancet. https://doi.org/10.1016/S0140-6736(23)00724-9
34. Stapleton SC (2019) Urgenci: international Network of community supported agriculture (urgenci.Net). J Agric Food Inf. 20, 196-205. https://doi.org/10.1080/10496505.2019.1630788
35. IPES-Food (2024) Food from somewhere: Building food security and resilience through territorial markets. Brussels: International panel of experts on sustainable food systems. https://ipes-food.org/wp-content/uploads/2024/06/FoodFromSomewhere.pdf. Accessed 24 September 2025.

36. Kam H, Potter C (2024) Who should deliver agri-environmental public goods in the UK? New land managers and their future role as public good providers. Land Use Policy, 139, 107072. https://doi.org/10.1016/j.landusepol.2024.107072
37. European Commission (2019) Contracts 2.0. Co-Design of Novel Contract Models for Innovative Agri-Environment Climate Measures and Valorisation of Environmental Public Goods. https://cordis.europa.eu/article/id/442659-incentivising-farmers-to-adopt-greener-agriculture. Accessed 24 September 2025.
38. Springmann M et al (2025) The health, environmental, and cost implications of providing healthy and sustainable school meals for every child by 2030: a global modelling study. Lancet Planet Health. https://doi.org/10.1016/j.lanplh.2025.06.002.
39. No Fry Zone 4 Kids (2018) https://data.oireachtas.ie/ie/oireachtas/committee/dail/32/joint_committee_on_children_and_youth_affairs/submissions/2018/2018-08-22_submission-no-fry-zone-4-kids-committee_en.pdf. Accessed 24 September 2025.
40. World Health Organization (2023) Launch of the WHO guideline on Policies to protect children from the harmful impact of food marketing. https://www.who.int/news-room/events/detail/2023/07/03/default-calendar/launch-of-the-who-guideline-on-policies-to-protect-children-from-the-harmful-impact-of-food-marketing. Accessed 24 September 2025.
41. Springmann M et al (2025) A reform of value-added taxes on foods can have health, environmental and economic benefits in Europe. Nat Food, 6(2):161-169. https://doi.org/10.1038/s43016-024-01097-5.
42. Kay S, Peuch J, Franco J (2015) Extent of Farmland Grabbing in the EU. European Parliament. https://www.europarl.europa.eu/RegData/etudes/STUD/2015/540369/IPOL_STU(2015)540369_EN.pdf. Accessed 24 September 2025.
43. Liu et al (2022) Agroecosystem services: A review of concepts, indicators, assessment methods and future research perspectives. Ecological Indicators, 142:109218. https://doi.org/10.1016/j.ecolind.2022.109218

44. European Commission (2019) Commission Regulation (EU) 2019/649 of 24 April 2019 amending Annex III to Regulation (EC) No 1925/2006 of the European Parliament and of the Council as regards trans fat, other than trans fat naturally occurring in fat of animal origin. https://eur-lex.europa.eu/legal-content/EN/TXT/?uri=CELEX:32019R0649. Accessed 24 September 2025
